# Selectively counteracting cerebellar adaptations to chronic alcohol exposure reduces acute alcohol withdrawal severity in C57BL6/N mice

**DOI:** 10.1016/j.neuropharm.2025.110595

**Published:** 2025-07-19

**Authors:** Nadia A. McLean, Samantha N. Shippell Stiles, Aspen E. Harder, Chloe M. Erikson, Gloria J. Lee, Dominik Schnalzer, Margot Ernst, Marko D. Mihovilovic, Giuseppe Giannotti, David J. Rossi

**Affiliations:** a Department of Integrative Physiology and Neuroscience, 1815 Ferdinands Lane, Washington State University, Pullman, WA, 99164-7620, USA; b Institute of Applied Synthetic Chemistry, TU Wien, Getreidemarkt 9/163, 1060, Vienna, Austria; c Medical University of Vienna, Division of Pathobiology of the Nervous System, Spitalgasse 4, A-1090, Vienna, Austria

**Keywords:** Cerebellum, Alcohol, EtOH, Withdrawal, GABA, Neuroadaptation, Negative affect, Motor impairment

## Abstract

A critical component of Alcohol Use Disorder (AUD) is alcohol (EtOH) withdrawal and consequent aversive withdrawal symptoms that generate negative reinforcement for renewed EtOH consumption to alleviate such symptoms. Here, we simulated human binge EtOH consumption and subsequent acute withdrawal by exposing male and female C57BL6/N mice to EtOH vapor for varying durations (24–72 h). During acute withdrawal, starting 4 h after removal from EtOH vapor, we used patch-clamp recording in cerebellar slices, combined with behavioral analysis of aversive somatic/motor (performance on the accelerating rotorod) and affective/emotional (ultrasonic vocalizations and blood corticosterone) withdrawal symptoms. We found that cerebellar granule cells (GCs) exhibit a homeostatic downregulation of sIPSC frequency that parallels development of motor discoordination and negative emotional affect, all starting at ~48–72 h of EtOH vapor exposure. Fitting with the negative reinforcement component of the AUD cycle, re-exposure to EtOH during withdrawal reduced somatic and affective withdrawal symptoms. Importantly, selective chemogenetic inhibition of GCs during withdrawal improved motor coordination, likely via actions at the GC axon terminals, and selective pharmacological inhibition of GCs via enhancement of the tonic GABA_A_R current, using PZ-II-029 (Compound 6), significantly improved negative emotional affect. Collectively, these results indicate that cerebellar homeostatic adaptations mediate aspects of both somatic and affective aversive EtOH withdrawal symptoms, and that restoration of cerebellar adaptations can effectively treat such symptoms. Moreover, they highlight the cerebellum as a promising selective target for treating aversive EtOH withdrawal symptoms, a critical component of the AUD cycle.

## 1. Introduction

Alcohol Use Disorder (AUD) is the leading substance use disorder in the United States (SAMHSA, 2021), resulting in widespread biomedical and socioeconomic harm (Hendriks, 2020; Sacks et al., 2015). Neurological adaptations to chronic alcohol (EtOH) exposure are critical for the development and maintenance of AUD, driving the transition from initial recreational EtOH consumption, motivated by EtOH’s rewarding properties, to chronic use, negatively reinforced by alleviation of withdrawal symptoms (Koob, 2013; Solomon and Corbit, 1974). Therefore, identifying neuroadaptations to chronic EtOH exposure and their role in aversive withdrawal symptoms could reveal pharmacological targets for breaking the AUD cycle.

Efforts to identify neurological mechanisms of EtOH withdrawal have primarily focused on how EtOH consumption alters established reward circuitry, in which initial exposure to EtOH enhances GABAergic inhibition and suppresses glutamatergic excitation, and chronic EtOH triggers compensatory adaptations, leading to reduced GABAergic inhibition and increased glutamatergic excitation (Alasmari et al., 2018; Lovinger and Roberto, 2023). This homeostatic transition leads to neuronal hyperexcitability and aversive withdrawal symptoms (Agoglia and Herman, 2018; Jesse et al., 2017). However, current treatments are not adequately addressing AUD relapse rates (Pareaud et al., 2021), suggesting a need for improved understanding of brain regions and neuronal mechanisms mediating EtOH withdrawal.

The cerebellum is highly sensitive to EtOH, responding to concentrations as low as 9 mM (Carta et al., 2004; Rossi and Richardson, 2018), and a primary mediator of motor impairment during acute EtOH exposure (Dar, 2015; Hanchar et al., 2005). While it is established that the cerebellum adapts to chronic EtOH exposure (Kumar et al., 2009; Marutha Ravindran et al., 2007; Mhatre and Ticku, 1992) and that motor impairment occurs during withdrawal (Metten and Crabbe, 2005; Philibin et al., 2008; Welsh et al., 2011), it is not known how cerebellar synaptic physiology is affected during withdrawal, nor if any such changes mediate EtOH withdrawal-induced motor impairment. Moreover, beyond its long-established role in motor coordination, the cerebellum is also involved in cognitive/emotional functions via projections to reward/addiction related pathways (Baek et al., 2022; Carta Ilaria et al., 2019; Jung et al., 2022; Kelly and Strick, 2003; Moreno-Rius, 2018; Schmahmann, 2019; Schmahmann and Pandya, 1997; Watson et al., 2014). Therefore, cerebellar adaptations during EtOH withdrawal likely contribute to motor and cognitive/emotional withdrawal symptoms.

Importantly, cerebellar responses to EtOH are opposite in high and low EtOH consuming rodents, which likely results in different homeostatic adaptations to chronic EtOH (Erikson et al., 2022; J. Kaplan et al., 2013). Most preclinical EtOH studies focus on high EtOH-consuming rodent genotypes that model human genetic risk for AUD, primarily the C57BL6/J mouse (Thiele et al., 2014). However, EtOH consumption is only 50–60 % genetically determined, indicating that environmental and adaptive factors that influence non-genetically predisposed subjects is critical when developing therapeutic strategies for AUD (Prescott and Kendler, 1999; Reilly et al., 2017).

To determine how the cerebellum contributes to EtOH withdrawal symptoms in a low EtOH consuming mouse genotype, we exposed male and female C57BL6/**N** mice to varying durations of chronic EtOH vapor (24–72 h). Four hours after removal from EtOH vapor, we used patch-clamp electrophysiology to characterize EtOH withdrawal-induced changes in granule cell (GC) inhibition, given that acute EtOH enhances GC GABA_A_R-mediated synaptic/phasic and extrasynaptic/tonic currents to varying degrees depending on rodent genotype (Carta et al., 2004; Erikson et al., 2022; J. Kaplan et al., 2013), which combine to decrease downstream Purkinje cell excitation (Kaplan et al., 2016) with consequent motor discoordination (Dar, 2015; Hanchar et al., 2005). We compared changes in GC GABAergic physiology to somatic/motor and affective/emotional manifestations of withdrawal, assessed via the accelerating rotarod and ultrasonic vocalizations (USVs), respectively. We found that EtOH withdrawal induced a downregulation of GC sIPSC frequency which paralleled the onset of aversive somatic and affective EtOH withdrawal symptoms. Finally, using GC-selective chemogenetics and pharmacology, we found that selective restoration of GC inhibitory tone reduced withdrawal symptoms, directly linking GC GABAergic dysregulation to behavioral manifestations of withdrawal.

## 2. Materials and methods

### 2.1. Subjects

Adult male and female C57BL6/N (B6N) mice (Jackson Laboratory-West) were given at least a week after arrival to acclimate before experimentation. GC-specific Gi-coupled Designer Receptors Exclusively Activated by Designer Drugs (GiDs) mice were bred in-house by crossing floxed Gi-coupled DREADD mice (bred on a B6N background) and *Gabra6*-Cre mice, a gene that is only expressed in cerebellar GCs (Aller et al., 2003). All GiD mice used in this study were homozygous for the Gi-coupled DREADD and either heterozygous (−) or homozygous (+) for the *Gabra6*-Cre. Homozygous *Gabra6*-Cre is required for expression, and thus membrane insertion, of functional DREADD receptors which is denoted in the figures using + or −. All mice (7+ weeks old) were single-housed with a 12:12 reverse light cycle and *ad libitum* access to food and water. All experiments were approved by Washington State University Institutional Animal Care and Use Committee.

### 2.2. 24/48/72-Hour EtOH vapor exposure

To induce EtOH withdrawal, we adapted the standardized 72-h vapor model (Terdal and Crabbe, 1994). Mice were primed with 1.5 g/kg EtOH (i.p.) and 68.1 mg/kg (i.p.) pyrazole (Sigma), or just pyrazole for air controls, daily before being placed in the passive vapor chambers (La Jolla Alcohol Research Inc.). Mice underwent 24, 48, or 72 h of air or EtOH vapor exposure. Blood samples (40–50 μL) were collected daily via tail snips and centrifuged at 10,000 rpm at 4 °C for 15 min to isolate plasma. Blood EtOH concentrations (BECs) were measured with the Analox GL5 (Analox Instruments). Vapor flow rate was adjusted daily to maintain BECs at ~40 mM (~175 mg/dl) (Metten and Crabbe, 2005). All withdrawal assessments were conducted 4 h after removal from the EtOH vapor chamber when EtOH is fully metabolized, corresponding to peak withdrawal (Finn and Crabbe, 1999).

### 2.3. Preparation of brain slices

Four hours after removal from vapor, mice were anesthetized using isoflurane, transcardially perfused with a chilled protective aCSF/sucrose solution containing (in mM): 26 NaHCO_3_, 1.25 NaH_2_PO_4_, 2 KCl, 5 MgCl_2_, 2 Na Pyruvate, 1 Ascorbic Acid, 10 D-glucose, 220 Sucrose, 0.5 CaCl_2_. After decapitation, the cerebellum was removed and the vermis was sliced sagittally at 225 μm in the protective aCSF/sucrose solution which was chilled to 1 °C. Slices were then incubated in standard aCSF (33 ± 1 °C) containing (in mM): 124 NaCl, 26 NaHCO_3_, 1 NaH_2_PO_4_, 2.5 KCl, 2.5 CaCl_2_, 2 MgCl_2_, 10 D-glucose, and bubbled with 95 %O_2_/5 % CO_2_ (pH 7.4) for 1 h after, and then held at room temperature until used. Broad glutamate receptor antagonist, kynurenic acid (1 mM) was included in the perfusion, dissection, incubation, and holding solutions, but was excluded from the experimental slice perfusion solutions.

### 2.4. Brain slice electrophysiology

Sagittal cerebellar vermis slices were placed in a submersion chamber on an upright microscope and viewed with an Olympus 60x (0.9 NA) water immersion objective. For GC recordings, in roughly equal proportions across lobules 2–8, GCs were visually identified and recorded from with whole cell voltage-clamp (V_h_ = −60 mV). Recordings were made with glass pipettes (5–10 MΩ) filled with a Cs-based internal solution containing (in mM): 130 CsCl, 4 NaCl, 0.5 CaCl_2_, 10 HEPES, 5 EGTA, 4 MgATP, 0.5 Na_2_GTP, 5 QX-314 Cl, in which $E_{Cl}^{-}=0\;mV$, except for the GiD recordings in which a K-gluconate internal was used containing (in mM): 132.3 K-gluconate, 7.7 KCl, 4 NaCl, 0.5 CaCl_2_, 10 HEPES, 5 EGTA, 4 MgATP, 0.5 Na_2_GTP with an $E_{Cl}^{-}=-60\;mV$. For Purkinje cell (PC) recordings, glass pipettes for cell-attached recordings were filled with aCSF. PCs were visually identified, and action potentials were recorded in voltage-clamp (0 mV) mode. Electrode resistance was 1–4 MΩ. For mossy fiber (MF) afferent stimulation experiments, a glass capillary stimulating electrode was placed in the white matter, and trains of stimuli (10 stims over 20 ms repeated every 30 s) were administered at intensities ranging from 30 to 500 mA (adjusted based on elicitation of stable PC action potential responses). All recordings took place under constant flow of bubbled carbogenated (5 %CO_2_/95 % O_2_) aCSF (32–35 °C, ~7 mL/min) in which GABAzine (10 μM, GBZ), Clozapine N-Oxide (10 μM, CNO), and PZ-II-029 (1.5–5 μM, Compound 6; C6) were diluted into aCSF, and bath applied. Recordings were acquired at 20 kHz and filtered at 10 kHz for holding current analysis and 2 kHz for sIPSC quantification, using pClamp software (Molecular Devices).

### 2.5. Accelerating rotorod

Mice were placed on a rotorod (San Diego Instruments) that accelerates from 5 to 40 rpm over the course of 260 s. Seven trials were conducted in each session, with 10-min breaks between each trial. Initial training sessions occurred daily for six days whereupon a steady baseline performance was reached. Then, mice underwent passive vapor exposure (air or EtOH). After removal from the vapor chamber, mice had rotorod sessions during intoxication (immediately upon removal from the vapor chamber), peak withdrawal (starting 4 h after removal from the vapor chamber), and recovery (24 h after vapor removal). Despite achieving a stable level of performance after 6 days of training, mice consistently exhibited improved performance over the first 3 trials of each session, and so the first 3 trials of each session were averaged together and considered rotorod learning, and the last 4 trials of each session were averaged together and considered rotorod performance. EtOH (1.5 g/kg), CNO (1 mg/kg), or Compound 6 (5 mg/kg) were injected intraperitoneally (i.p.) immediately before the first trial of the withdrawal session.

### 2.6. Ultrasonic vocalizations (USVs)

Four hours after removal from vapor, mice were placed in a restraint tube for 10-min, which acted as a mild stressor used to elicit calls (stress reactivity) (Chabout et al., 2012; Portfors, 2007). In insulated soundproof chambers, an Avisoft UltraSoundGate 116 microphone (Avisoft Bioacoustics) was used to record USVs with a sampling frequency of 250 kHz. DeepSqueak software was used to analyze the call frequencies. EtOH (1.5 g/kg), CNO (1 mg/kg), or Compound 6 (5 mg/kg) were injected intraperitoneally 30 min before their USV session.

### 2.7. Corticosterone (CORT) measurements

Blood samples were collected as described above, 24 h before being placed in the vapor chambers as a baseline value. Blood was taken twice during withdrawal, immediately upon placement in a restraint tube and after 15 min of restraint to measure basal stress levels and stress reactivity respectively (Zimprich et al., 2014). Plasma CORT levels were analyzed using the enzyme-linked immunosorbent assay (ELISA) plasma CORT test kit (Arbor Assays).

### 2.8. Statistical analyses

For the sake of readability of the text, but full conveyance of statistics, normality tests and key statistical information is provided within the text and/or figure legends, but the full statistical analytical details are reported in the supplementary table (Table S1). Statical analyses were conducted using SigmaPlot and SPSS with statistical significance defined as *p* < 0.05. For electrophysiology experiments, two-way analyses of variance (ANOVAs) were used to compare differences in sex, vapor/treatment condition, and the dependent variable unless otherwise specified. For behavioral experiments, mixed factorial ANOVAs were used to compare differences between sex, vapor/treatment condition, experimental trial, and the dependent variable unless otherwise specified. Interactions and main effects are listed in figure legends. Post hoc Sidak pairwise multiple comparisons are stated via individual p-values or asterisks. Non-parametric data was analyzed using ANOVAs on ranks or Mann-Whitney Rank Sum tests. Cells were dropped from the sIPSC frequency analysis if their basal frequency exceeded two standard deviations from the mean. Cells were dropped from sIPSC amplitude, basal holding current, and tonic GABA_A_R current analyses if the access resistance deviated by more than 20 % across the recording period. All data are presented as mean ± SEM.

### 2.9. Reagents

All chemicals used in electrophysiology solutions were purchased from Sigma except SR95531 hydrobromide (GABAzine) which was purchased from Abcam. CNO (HelloBio) was dissolved in ultrapure water and diluted in aCSF for bath application during electrophysiology recordings (10 μM) or sterile 0.9 % saline for i.p. injections (1 mg/kg). Compound 6 was provided by the Research Group Bioorganic Synthetic Chemistry at TU Wien (Vienna, Austria). For electrophysiology, Compound 6 was dissolved in DMSO or TWEEN-80 and diluted with aCSF for bath application (1–5 μM). For *in vivo* studies, Compound 6 was diluted in TWEEN-80 and combined with Cremophor-EL (Sigma), and sterile 0.9 % saline in a 7:20:73 ratio (5 mg/kg). To avoid precipitation, the solution was warmed to 33 ± 1 °C before combining all the components.

## 3. Results

### 3.1. Cerebellar phasic but not tonic GABA_A_R inhibition downregulates after 48 h of EtOH vapor exposure

**Fig. 1.**
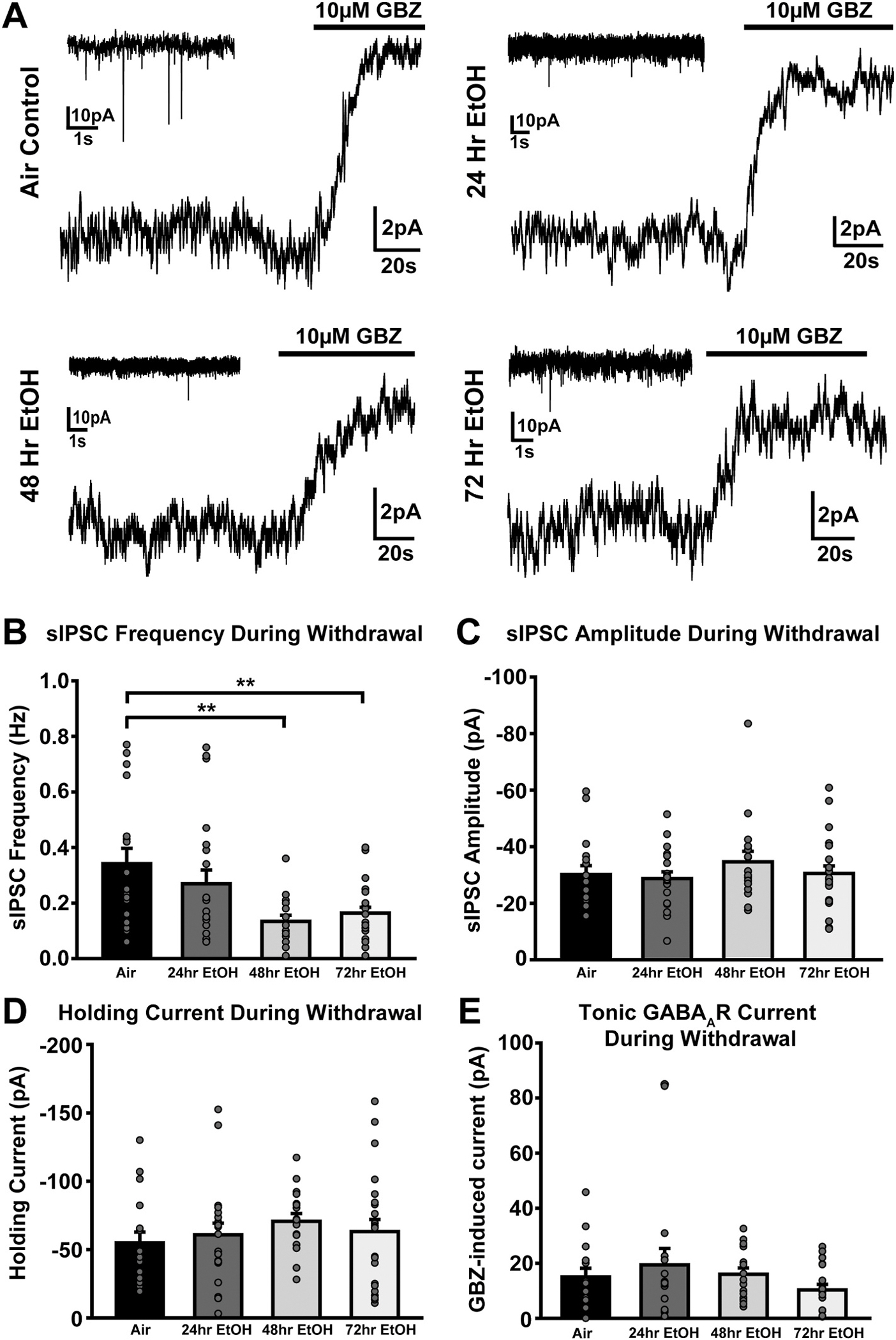
Cerebellar phasic but not tonic GABA_A_R inhibition downregulates after 48 h of EtOH vapor exposure. **A.** Representative traces of recordings from voltage-clamped (V_h_ = −60 mV, $E_{Cl}^{-}=0\;mV$) cerebellar granule cells highlighting tonic GABA_A_R current (bottom) and sIPSC frequency and amplitude (insets) during acute withdrawal from 72 h of air exposure (top left) or 24 (top right), 48 (bottom left), or 72 h (bottom right) of EtOH exposure. Magnitude of tonic GABA_A_R current is quantified by the change in holding current before and after application of the GABA_A_R antagonist GABAzine (GBZ; 10 μM). **B-E.** Bar charts show mean sIPSC frequency (**B**), sIPSC amplitude (**C**), basal holding current (**D**), and tonic GABA_A_R current (**E**) across different groups during withdrawal±standard error. Data were analyzed with Kruskal-Wallis ANOVAs on Ranks with Dunn’s post-hoc comparisons and Two-Way ANOVAs with Sidak post-hoc comparisons. **B.** EtOH Duration × Sex: F_(3,70)_ = 0.735, *p* = 0.735; EtOH Duration: F_(3,70)_ = 5.101, *p* = 0.003, H(3) = 12.538, p = 0.006; Sex: F_(1,70)_ = 0.787, *p* = 0.378. **C.** EtOH Duration × Sex: F_(3,69)_ = 0.645, *p* = 0.589; EtOH Duration**:** F_(3,69)_ = 0.685, *p* = 0.564, H(3) = 1.698, p = 0.637; Sex: F_(1,69)_ = 1.485, *p* = 0.227 **D.** EtOH Duration × Sex: F_(3,69)_ = 0.838, *p* = 0.478; EtOH Duration**:** F_(3,69)_ = 0.817, *p* = 0.489, H(3) = 3.684, p = 0.298; Sex: F_(1,69)_ = 3.142, *p* = 0.081 **E.** EtOH Duration × Sex: F_(3,62)_ = 1.231, *p* = 0.306; EtOH Duration**:** F_(3,62)_ = 1.182, *p* = 0.324, H(3) = 2.970, p = 0.396; Sex: F_(1,62)_ = 4.074, *p* = 0.048, Mann-Whitney U = 483, n1 = 33, n2 = 37, p = 0.135. (*p = 0.05, **p = 0.005, ***p < 0.001).

To determine how and when the cerebellum adapts to chronic EtOH exposure, we performed voltage-clamp recordings from cerebellar granule cells (GCs; V_h_ = −60 mV, $E_{Cl}^{-}=0\;mV$) during acute withdrawal, starting 4 h after removal from vapor (Fig. 1). No significant differences were found between the anterior (2–5) and posterior lobules (6–8) across the different EtOH durations in terms of sIPSC frequency (Kruskal-Wallis ANOVA on Ranks, H(7) = 11.421, p = 0.121), sIPSC amplitude (Two-way ANOVA, F(66,1) = 0.374, p = 0.543), or tonic GABA_A_R current magnitude (Kruskal-Wallis ANOVA on Ranks, H(7) = 5.983, p = 0.542), therefore the data from all lobules were combined (see Table S1 for statistical details). We observed a significant decrease in sIPSC frequency following withdrawal from 48- (*p* = 0.001) and 72-h (*p* = 0.002) EtOH exposure, but not after 24-h (*p* = 0.178), compared to 72-h air controls (Fig. 1A and B). There were no significant differences in sIPSC amplitude, basal holding current, or tonic GABA_A_R current between the different EtOH durations and air controls (Fig. 1C–E). Together, our data show that the cerebellum begins downregulating inhibitory tone, in the form of reduced sIPSC frequency, beginning as early as after 48-h of EtOH exposure.

### 3.2. The development of withdrawal-induced motor impairment coincides with cerebellar adaptations at 48- and 72-h of EtOH exposure

**Fig. 2.**
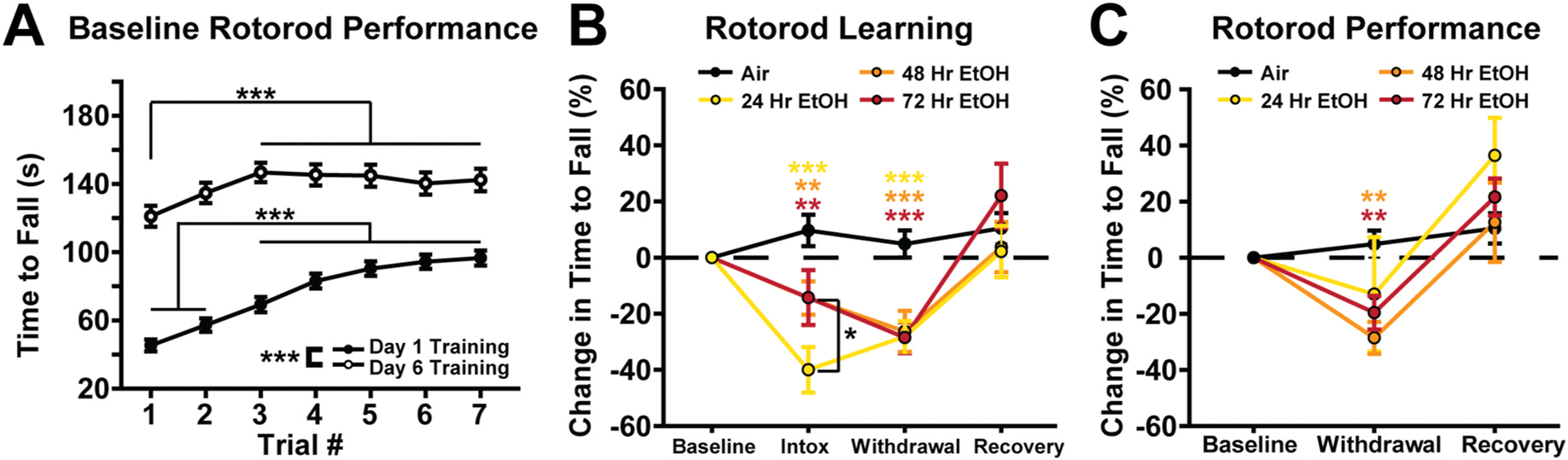
The development of withdrawal-induced motor impairment coincides with cerebellar adaptations at 48- and 72-h of EtOH exposure. **A.** Differences in latency to fall within and between the first and sixth day of rotorod training (baseline). **B.** Comparison in rotorod learning (defined as the first three trials per session with each condition normalized to baseline performance) during intoxication, withdrawal, and recovery across all EtOH durations and air controls. Color coded asterisks indicate a difference compared to air controls unless otherwise noted. **C.** Comparison in rotorod performance (defined as the last four trials per session with each condition normalized to baseline performance) during withdrawal and recovery across all EtOH conditions and air controls. Color coded asterisks indicate a difference compared to air controls. Note, because blood EtOH is metabolized rapidly by mice, motor performance (the last 4 trials, which occur >30 min after removal from vapor chambers) cannot be quantified during intoxication. Data were analyzed using Mixed Factorial ANOVAs with Sidak post hoc comparisons. **A.** Training Day × Trial × Sex: F_(6,324)_ = 0.942, *p* = 0.465, Training Day × Sex: F_(1,324)_ = 0.372, *p* = 0.545, Trial × Sex: F_(6,324)_ = 1.014, *p* = 0.410, Training Day × Trial: F_(6,324)_ = 9.589, *p* < 0.001, Training Day: F_(1,324)_ = 179.953, *p* < 0.001, Trial: F_(6, 324)_ = 29.724, *p* < 0.001, Sex: F_(1,54)_ = 4.898, *p* = 0.001. **B.** EtOH Duration × Sex × Session: F_(9,144)_ = 0.809, *p* = 0.593, EtOH Duration × Sex: F_(3,48)_ = 0.427, *p* = 0.073, EtOH Duration × Session: F_(3,144)_ = 6.231, *p* < 0.001, EtOH Duration: F_(3,48)_ = 5.072, *p* = 0.004, Session: F_(3,144)_ = 24.809, *p* < 0.001, Sex: F_(1,48)_ = 3.471, *p* = 0.069. **C.** EtOH Duration × Sex × Session: F_(9,96)_ = 0.861, *p* = 0.507, EtOH Duration × Sex: F_(3,48)_ = 0.705, *p* = 0.553, EtOH Duration × Session: F_(3,96)_ = 0.861, *p* < 0.001, EtOH Duration: F_(3,48)_ = 1.254, *p* = 0.301, Session: F_(2,96)_ = 37.344, *p* < 0.001, Sex: F_(1,48)_ = 0.602, *p* = 0.442. (*p < 0.05, **p < 0.005, ***p < 0.001).

To assess motor performance, we assessed the time to fall from the accelerating rotorod at baseline (pre-vapor exposure) and during three different withdrawal time points: intoxication (immediately following removal from the vapor chamber), acute withdrawal (beginning 4-h post-exposure), and recovery (24-h after vapor cessation). These assessments were performed in mice exposed to chronic EtOH or air for varying durations (24, 48 and 72 h; Fig. 2). All mice significantly improve their rotorod performance from day 1 of training to day 6 (Fig. 2A). Within-session improvements were also observed across training days. In particular, on training day 1, performance improved significantly across trials, with trials 1–2 showing shorter latencies to fall compared to trials 4–7 (Fig. 2A, see Table S1). Notably, even after mice reach overall steady state performance by day 6 of training, a significant within session improvement persisted across trials, with trial 1 having a shorter latency to fall compared to trials 3–7 (Fig. 2A, see Table S1). Therefore, rotorod experiments are broken into learning, defined as the first three trials of each session, and performance, defined as the last four trials of each session. Importantly, there are no differences in learning (p = 0.818) or performance (p = 0.725) between mice exposed to 24, 48 and 72 h of air vapor. Therefore, all air controls are collapsed for subsequent comparisons (Fig. 2B and C).

When assessing motor learning, we found that depending on the duration, EtOH exposure caused tolerance to motor impairment during intoxication and increased motor impairment during withdrawal (Fig. 2B and C). All EtOH exposed animals, regardless of their EtOH exposure duration, perform significantly worse than air controls during intoxication (24-h EtOH, *p* < 0.001; 48-h EtOH, *p* = 0.005; 72-h EtOH, *p* = 0.005; Fig. 2B). However, 24-h EtOH exposed mice not only perform worse than air controls, but they also perform worse than the 48- (p = 0.011) and 72-h (p = 0.011) EtOH exposed mice during intoxication (Fig. 2B). This indicates that once the cerebellum has adapted to chronic EtOH, there is an increased tolerance to impairment of motor learning while intoxicated. Additionally, mice in all EtOH durations have impaired motor learning during withdrawal (starting 4 h after vapor removal) compared to air controls (24-h EtOH, *p* < 0.001; 48-h EtOH, *p* < 0.001; 72-h EtOH, *p* < 0.001; Fig. 2B).

When assessing motor performance, we found that EtOH reduced rotorod performance during withdrawal, but, only after 48- (*p* = 0.001) and 72-h (*p* = 0.004), not 24-h of EtOH exposure, compared to air controls (Fig. 2C). Mice could not be assessed for motor performance during intoxication because EtOH is rapidly metabolized during the 3 trials of the learning phase (~35 min from removal from vapor chambers). Taken together, all EtOH durations impair motor learning during both intoxication (although less so after longer durations, indicative of tolerance) and withdrawal, but motor performance during withdrawal is only impaired after 48- and 72-h of EtOH exposure.

### 3.3. The development of negative emotionality during withdrawal coincides with cerebellar adaptations at 72-h of EtOH exposure

**Fig. 3.**
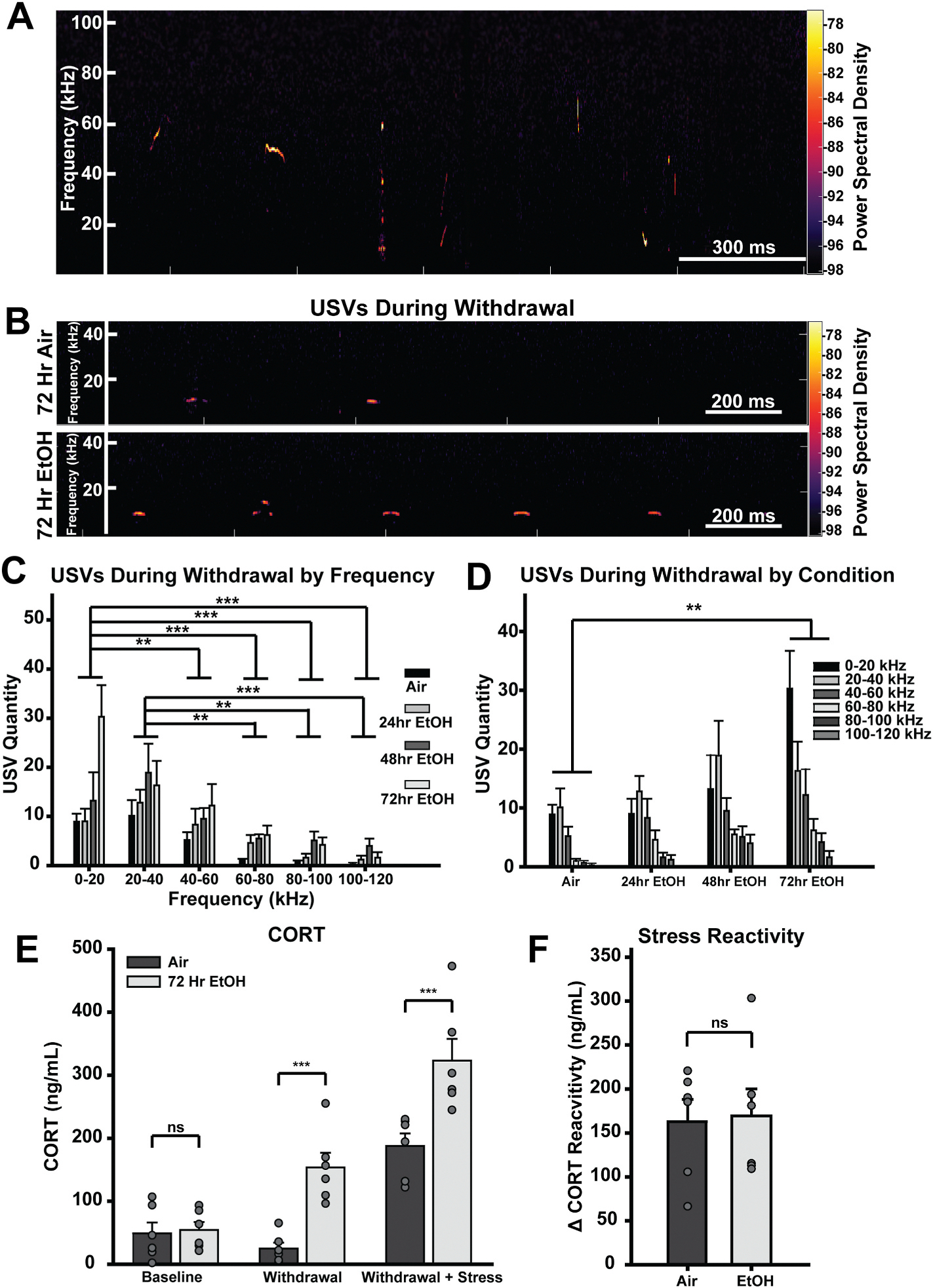
The development of negative emotionality during withdrawal coincides with cerebellar adaptations at 72-h of EtOH exposure. **A.** Example USV trace showing the variety of calls of different frequencies over time. **B.** Examples of USVs in the 0–40 kHz range for air (top) and EtOH (bottom) exposed mice during withdrawal. **C-D.** Mean USV quantity clustered by frequency (kHz) (**C**) and EtOH duration (**D**) ± standard error. **E.** Mean CORT levels±SE during baseline, onset of withdrawal, and after 15 min of restraint stress during withdrawal. **F.** Mean stress reactivity quantified as the difference in CORT before and after restraint stress during withdrawal. Data were analyzed using Mixed Factorial ANOVAs with Sidak post hoc comparisons. **C-D.** EtOH Duration × Sex × Frequency: F_(15,160)_ = 0.892, *p* = 0.511, EtOH Duration × Frequency: (F_(15,160)_ = 2.466, *p* = 0.029), EtOH Duration × Sex: F_(1,32)_ = 1.834, *p* = 0.161, Sex × Frequency: F_(5,160)_ = 0.248, *p* = 0.795, EtOH Duration: F_(3,32)_ = 2.740, *p* = 0.059, Frequency: F_(5,160)_ = 23.454, *p* < 0.001, Sex: F_(1,32)_ = 0.036, *p* = 0.852. **E.** Vapor Treatment × Sex × Session: F_(2,16)_ = 0.376, *p* = 0.004, Vapor Treatment × Session: F_(1,16)_ = 20.249, *p* = 0.001, Vapor Treatment × Sex: F_(1,8)_ = 1.970, *p* = 0.198, Sex × Session: F_(2,16)_ = 3.373, *p* = 0.060, Vapor Treatment: F_(1,8)_ = 27.496, *p* = 0.001, Session: F_(2,16)_ = 4.951, *p* < 0.001, Sex: F_(1,8)_ = 3.610, *p* = 0.094. (*p < 0.05, **p < 0.005, ***p < 0.001).

To assess emotional affect with as little motoric contribution as possible, we measured ultrasonic vocalizations (USVs) during withdrawal, 4 h after removal from EtOH vapor (Fig. 3A–D). Although not well established in mice, studies in rats indicate that low-frequency USVs reflect negative affect, whereas high-frequency calls typically indicate positive affect (Simola and Granon, 2019). In all conditions, mice elicited the majority of their calls in the low 0–40 kHz range (see Table S1, Fig. 3C). However, during withdrawal from 72-h EtOH, mice elicited significantly more low frequency calls compared to air controls (*p* = 0.002; Fig. 3B–D). This suggests that negative emotionality occurs during withdrawal from 72 h of vapor exposure.

To confirm that similar to rats, low frequency USVs in mice serve as a measurement of negative affect during withdrawal, in a separate cohort, we measured blood corticosterone (CORT) levels in mice withdrawn from 72 h of EtOH vapor compared to air controls (Fig. 3E and F). Blood was collected prior to vapor exposure, during acute withdrawal (4 h after removal from vapor), and after 15-min of restraint stress during withdrawal. We found that there was no difference in CORT at baseline prior to vapor exposure (*p* = 0.856; Fig. 3E). However, EtOH exposed mice had significantly higher CORT levels at withdrawal onset compared to air controls (*p* < 0.001; Fig. 3E). This increase in CORT levels was further exacerbated by 15 min of restraint stress during withdrawal (*p* < 0.001; Fig. 3E). However, when stress reactivity was assessed as the change in CORT levels (ΔCORT) before and after restraint stress, no differences were observed between air and EtOH withdrawn mice (*t*-test, t(10) = −0.165, *p* = 0.872; Fig. 3F), suggesting that differences in CORT levels reflect elevated basal stress during withdrawal rather than a heightened sensitivity to an acute stressor. Thus, the increase in low frequency USVs from EtOH withdrawn mice coincides with elevated physiological markers of stress, supportive of it reflecting negative emotional affect.

### 3.4. EtOH administration during withdrawal improves behavioral withdrawal symptoms

**Fig. 4.**
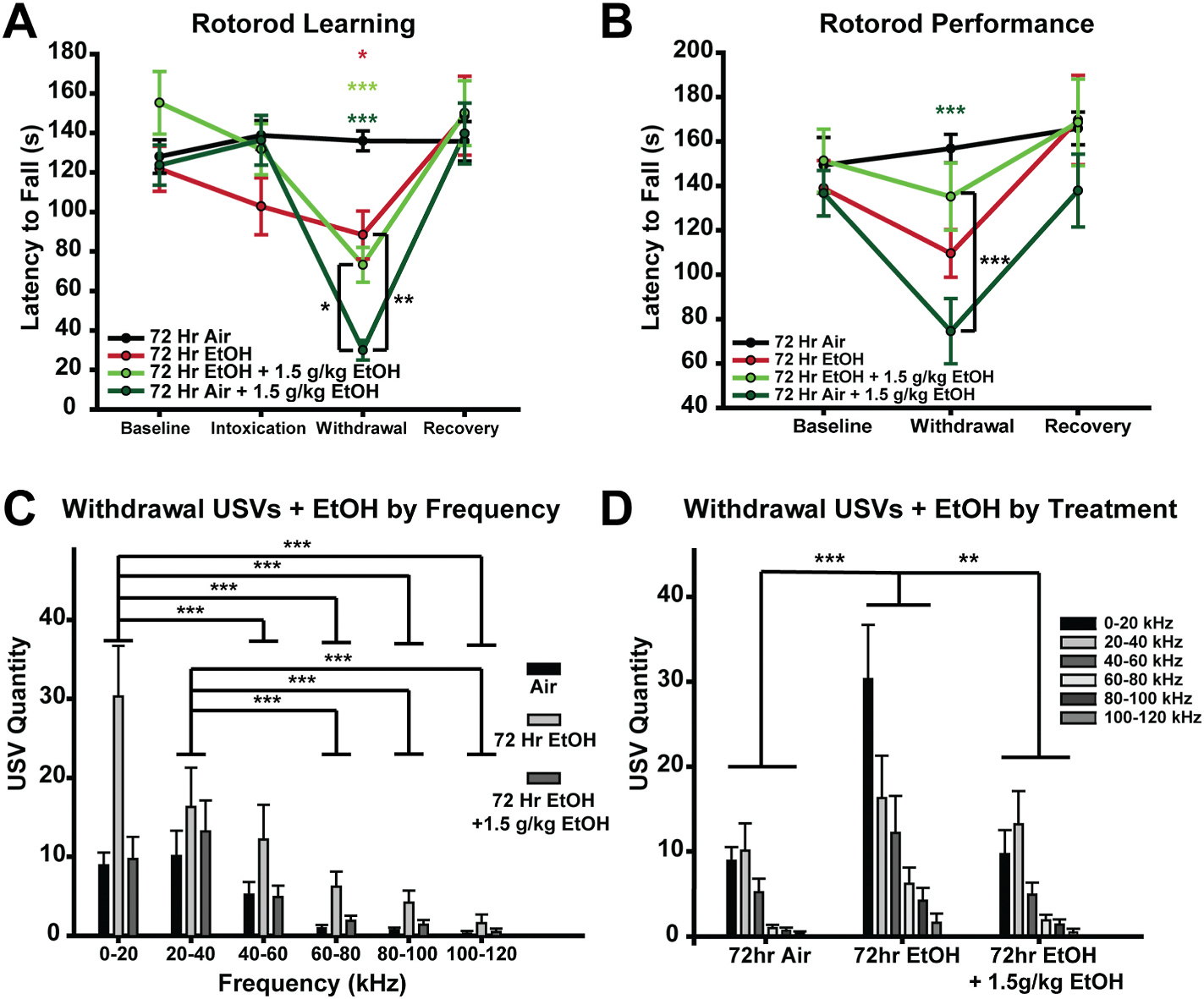
EtOH administration during withdrawal improves behavioral withdrawal symptoms. **A.** Comparison in rotorod learning (first three trials per session standardized to baseline performance) during intoxication, during withdrawal with or without i.p. EtOH (1.5 g/kg), and recovery. **B.** Comparison in rotorod performance (last four trials per session standardized to baseline performance) during withdrawal with or without i.p. EtOH (1.5 g/kg), and recovery. Color coded asterisks above the line indicate a difference compared to air controls. **C&D.** Mean USV quantity clustered by frequency (kHz) (**C**) and EtOH Treatment (**D**) ± standard error. Data were analyzed using Mixed Factorial ANOVAs with Sidak post hoc comparisons. **A.** EtOH Treatment × Sex × Session: F_(9,90)_ = 8.652, *p* < 0.001, EtOH Treatment × Session: F_(9,90)_ = 8.491, *p* < 0.001, EtOH Treatment × Sex: F_(3,30)_ = 1.435, *p* = 0.252, Sex × Session: F_(3,90)_ = 0.596, *p* = 0.619, EtOH Treatment: F_(3,30)_ = 1.465, *p* = 0.244, Session: F_(3,90)_ = 40.523, *p* < 0.001, Sex: F_(1,30)_ = 1.680, *p* = 0.205. **B.** EtOH Treatment × Sex × Session: F_(6,60)_ = 2.373, *p* = 0.040, EtOH Treatment × Session F_(6,60)_ = 3.802, *p* = 0.003, EtOH Treatment × Sex: F_(3,30)_ = 0.932, *p* = 0.438, Sex × Session: F_(2,60)_ = 0.790, *p* = 0.459, EtOH Treatment: F_(3,30)_ = 0.2039, *p* = 0.130, Session F_(2,60)_ = 25.117, *p* < 0.001, Sex: F_(1,30)_ = 0.307, *p* = 0.584. **C-D.** EtOH Treatment × Sex × Frequency: F_(10,120)_ = 0.919, *p* = 0.469, EtOH Treatment × Frequency: F_(10,120)_ = 3.691, *p* = 0.007, EtOH Treatment × Sex: F_(2,24)_ = 2.430, *p* = 0.109, Sex × Frequency: F_(5,120)_ = 0.115, *p* = 0.115, EtOH Treatment: F_(2,24)_ = 4.928, *p* = 0.016, Sex: F_(1,24)_ = 1.022, *p* = 0.322, Frequency: F_(5,120)_ = 25.942, *p* < 0.001. (*p < 0.05, **p < 0.005, ***p < 0.001).

EtOH withdrawal symptoms play a critical role in the negative reinforcement aspect of the AUD cycle, where homeostatic adaptations to chronic EtOH and the consequent aversive withdrawal symptoms are relieved by renewed EtOH consumption (Koob and Volkow, 2010). In this context, it is well established that acute EtOH application increases cerebellar GC inhibition (Carta et al., 2004; Erikson et al., 2022; J. Kaplan et al., 2013), and here we show that prolonged EtOH exposure results in a compensatory reduction of GC inhibitory tone (Fig. 1). Thus, we predicted that EtOH administration during withdrawal would reduce the magnitude of cerebellar-dependent EtOH withdrawal symptoms. To determine whether acute EtOH reduces motor impairment during withdrawal from 72 h of EtOH vapor, we repeated rotorod experiments (as in Fig. 2) but intraperitoneally injected mice with EtOH (1.5 g/kg) prior to testing during withdrawal (Fig. 4). This dose was chosen as it achieves blood EtOH concentration of ~30 mM (32.47±4.75 mM, N = 14, data not shown) which is close to the concentration experienced during EtOH vapor exposure. I.p. administration of 1.5 g/kg EtOH did not restore the motor learning impairment in EtOH withdrawn mice (Fig. 4A). However, EtOH withdrawn mice injected with EtOH performed significantly better then air exposed controls injected with the same concentration of EtOH, indicating that tolerance to EtOH-mediated impairment of motor learning developed. Specifically, EtOH withdrawn mice that were treated with EtOH were not significantly different than untreated EtOH withdrawn mice (*p* = 0.380), where both groups had similar significantly shorter latencies to fall when compared to untreated air controls (EtOH treated EtOH withdrawn mice, *p* < 0.001; untreated EtOH withdrawn mice, *p* = 0.007; Fig. 4A). In contrast, EtOH administration to air withdrawn mice not only makes them worse than untreated air controls (*p* < 0.001), but it also impairs rotorod learning when compared to EtOH withdrawn untreated (*p* = 0.002) and EtOH withdrawn mice treated with 1.5 g/kg EtOH (*p* = 0.020). Thus, while EtOH administration does not improve or impair rotorod learning in EtOH withdrawn mice, it does significantly reduce EtOH-induced impairment in rotorod learning compared to air control mice.

When assessing the impact of EtOH administration on motor performance, i.p. administration of 1.5 g/kg EtOH to EtOH withdrawn mice eliminates motor deficits in rotorod performance during withdrawal, making them no different than untreated air controls (*p* = 0.266; Fig. 4B). Importantly, EtOH administration in air withdrawn mice significantly impairs motor performance compared to untreated air controls (*p* < 0.001) and EtOH treated EtOH withdrawn mice (*p* = 0.004). This demonstrates that EtOH administration improves rotorod performance in EtOH withdrawn mice but impairs rotorod performance in air withdrawn mice.

Next, to determine whether the EtOH-induced restoration of cerebellar inhibitory tone improves negative emotional affect during withdrawal from 72 h of EtOH vapor, we collected USVs during withdrawal, but again administered 1.5 g/kg EtOH immediately prior to USV recording (Fig. 4C and D). We found that EtOH administration significantly reduced the quantity of USVs compared to untreated EtOH withdrawn mice (*p* = 0.003; Fig. 4C and D), making them no different than untreated air controls (*p* = 0.585). Together, these data indicate that renewed intoxication during EtOH withdrawal reduces both the motor performance impairments and negative emotional affect induced by EtOH withdrawal.

### 3.5. Selective chemogenetic inhibition of cerebellar granule cells during withdrawal reduces motoric but not emotional symptoms during EtOH withdrawal

**Fig. 5.**
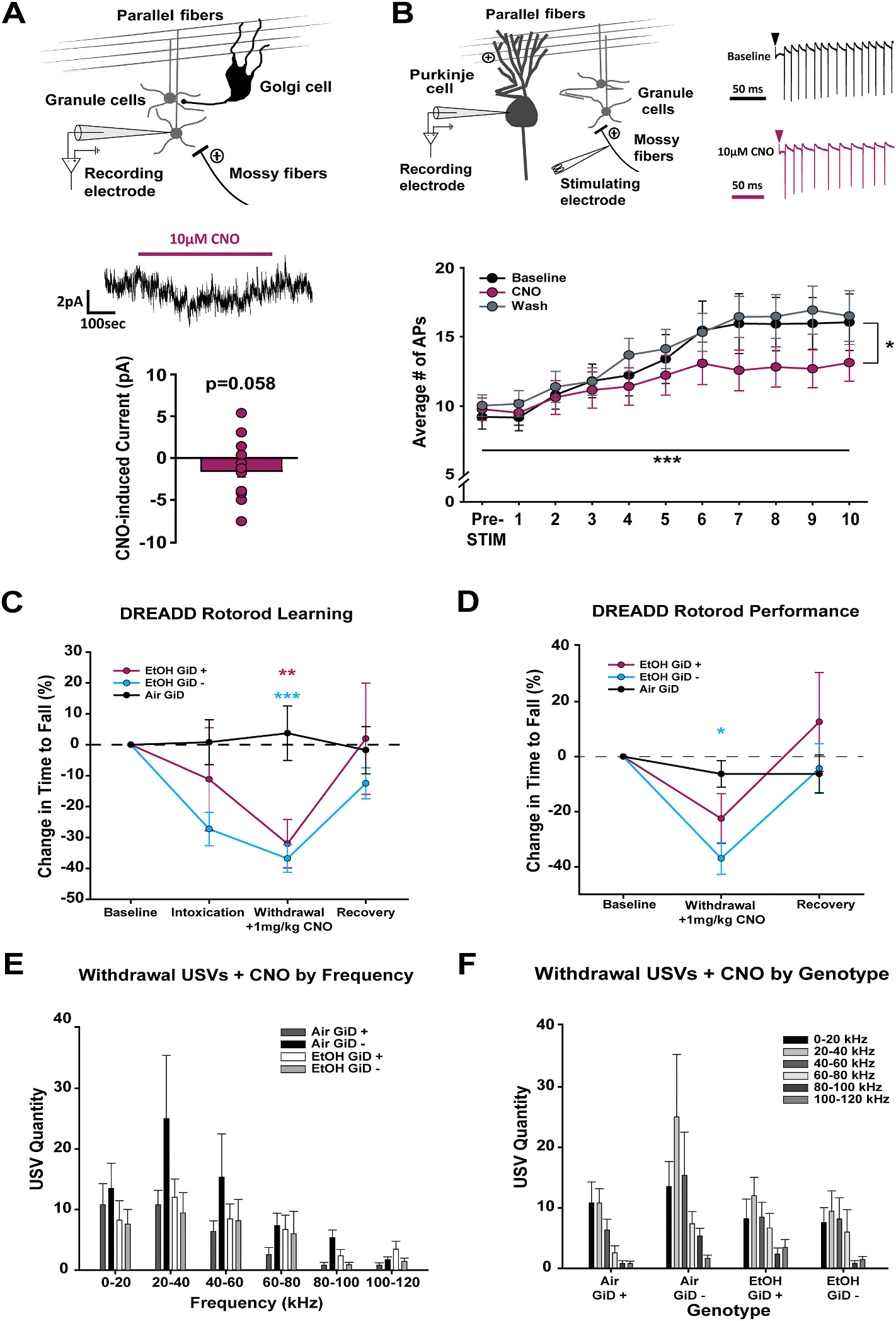
Selective chemogenetic inhibition of cerebellar granule cells during withdrawal reduces motoric but not emotional symptoms during EtOH withdrawal. **A.** Diagram showing cerebellar circuitry and electrophysiology for whole cell recordings from GCs (**Top**) and Mean change in current in response to CNO application (**bottom**). **B.** Diagram and electrophysiology showing cell attached action potential recordings from Purkinje Cells (PCs) with mossy fiber (MF) stimulation (**Top**) and mean # of PC action potentials evoked by trains of MF stimulation (**Bottom**). **C.** Comparison in rotorod learning (first three trials per session standardized to baseline performance) during intoxication, during withdrawal + 1 mg/kg CNO, and recovery in GiD positive or negative mice. Color coded asterisks indicate a difference compared to air controls. Air GiD + and – mice were not significantly different and collapsed. **D.** Comparison in rotorod performance (last four trials per session standardized to baseline performance) during withdrawal + 1 mg/kg CNO and recovery in GiD positive or negative mice. **E&F.** Mean USV quantity clustered by frequency (kHz) (**E**) and Genotype (**F**) ± standard error. Functional GiD expressing mice denoted by + and −. Data in **B** were analyzed using a Two-Way RM ANOVA and Sidak post hoc comparisons. Drug × Stimulation Number: F_(20,260)_ = 2.502, *p* < 0.001, Stimulation Number: F_(10,260)_ = 17.448, *p* < 0.001, Drug: F_(13,260)_ = 3.116, *p* = 0.061. Data in **C-F** were analyzed using Mixed Factorial ANOVAs with Sidak post hoc comparisons. **C.** Genotype × Sex × Session: F_(6,102)_ = 1.034, *p* = 0.057, Genotype × Sex: F_(2,34)_ = 0.027, *p* = 0.973, Genotype × Session: F_(6,102)_ = 3.176, *p* = 0.013, Sex × Session: F_(3,102)_ = 0.558, *p* = 0.602, Genotype: F_(2,34)_ = 2.264, *p* = 0.119, Session: F_(3,102)_ = 6.188, *p* = 0.002, Sex: F_(1,34)_ = 0.055, *p* = 0.816. **D.** Genotype × Sex × Session: F_(2,68)_ = 1.018, *p* = 0.056, Genotype × Sex: F_(2,34)_ = 0.525, *p* = 0.596, Genotype × Session: F_(2,68)_ = 3.347, *p* = 0.028, Sex × Session: F_(2,68)_ = 0.038, *p* = 0.920, Genotype: F_(2,34)_ = 0.902, *p* = 0.415, Session: F_(2,68)_ = 12.070, *p* < 0.001, Sex: F_(1,34)_ = 0.062, *p* = 0.804. **E-F.** Genotype × Sex × Frequency: F_(15,115)_ = 0.338, *p* = 0.934, Genotype × Sex: F_(3,24)_ = 0.515, *p* = 0.676, Genotype × Frequency: F_(15,115)_ = 0.725, *p* = 0.653, Sex × Frequency: F_(5,115)_ = 0.418, *p* = 0.692, Genotype: F_(3,23)_ = 1.480, *p* = 0.246, Frequency: F_(5,115)_ = 10.019, *p* < 0.001, Sex: F_(1,23)_ = 0.000, *p* = 0.989. (*p < 0.05, **p < 0.005, ***p < 0.001).

To determine whether the observed homeostatic downregulation of GC inhibitory tone (Fig. 1) is necessary for somatic and/or affective withdrawal symptoms, we utilized GC specific inhibitory DREADD mice (GiD), generated by crossing floxed Gi-DREADD mice with *Gabra6-Cre mice*, which express Cre-recombinase under the GABA_A_R α6 subunit promoter, selectively expressed by cerebellar GCs (Aller et al., 2003; Kato, 1990) (Fig. 5). To validate the functionality of GiDs, we performed whole-cell patch-clamp recordings from cerebellar GCs of GiD mice (Fig. 5A). Contrary to expectations, when using a K-Gluconate based internal solution, on average bath application of 10 μM CNO elicited a small but insignificant inward current (Fig. 5A, bottom; paired *t*-test, t (14) = 2.066, *p* = 0.058). Given this unexpected outcome and the possibility that GiDs are primarily expressed on GC axon terminals (Campbell and Marchant, 2018), we performed cell-attached recordings from downstream cerebellar Purkinje cells (PCs; Fig. 5B). Cerebellar GCs form excitatory connections onto PCs through parallel fibers, making PCs a suitable target to assess functional changes in GC signaling, via cerebellar cortical afferent mossy fiber (MF) stimulation. MF stimulation significantly increased PC firing (Fig. 5B, bottom). Importantly, bath application of 10 μM CNO significantly reduced the number of evoked action potentials compared to baseline (Fig. 5B, bottom; *p* = 0.038). Together, these results demonstrate that CNO application in GiD mice inhibits cerebellar GC activity as evidenced by reduced number of MF-evoked action potentials in downstream PCs. This effect is likely mediated by inhibition of parallel fiber axons and/or their vesicular release of glutamate onto PCs rather than at the GC soma.

To determine whether chemogenetic inhibition of cerebellar GCs reduces the severity of EtOH withdrawal symptoms, we administered CNO (1 mg/kg, i.p.) during withdrawal from 72 h of EtOH or air vapor and assessed motor coordination (rotorod; Fig. 5C and D) and negative emotional affect (USVs; Fig. 5E and F). There were no significant interactions between rotorod session and genotype (GiD + or −) in air exposed GiD mice in terms of rotorod learning (Two-way RM ANOVA, F_(3,54)_ = 0.154, *p* = 0.119) or performance (Mixed Factorial ANOVA, F_(2,36)_ = 0.617, *p* = 0.545). Thus, air exposed groups (GiD + and −) were collapsed for subsequent comparisons to EtOH withdrawn mice of each genotype. During withdrawal, CNO administration did not significantly improve rotorod learning, with both GiD-positive (GiD +, *p* = 0.003) and GiD-negative (GiD −, *p* < 0.001) EtOH exposed mice showing similar impairments relative to air exposed mice of both genotypes (Fig. 5C). However, when comparing rotorod performance between EtOH exposed GiD mice and air controls, selective inhibition of GCs significantly improved motor performance during withdrawal, with CNO treated GiD-positive (*p* = 0.106), but not GiD negative mice (*p* = 0.003), performing as well as air exposed mice of both genotypes (Fig. 5D).

Unexpectedly, there were no significant differences among USV frequency vocalizations across the different GiD genotypes (Fig. 5E and F). Moreover, EtOH withdrawn GiD negative mice (i.e. controls) did not vocalize more than air exposed controls (*p* = 0.329), in contrast to our observations in non-genetically modified B6N mice (Fig. 3). This suggests the genetic modification associated with GiD expression itself may have altered USV responses to EtOH withdrawal, complicating the interpretation of our findings. Together, this suggests that GiD activation during withdrawal improves motor impairment but that the genetic modification has affected USVs or emotional affect in a manner that prevents accurate testing during EtOH withdrawal.

### 3.6. PZ-II-029, compound 6, is a novel GABA_A_R positive allosteric modulator that is selective for GC GABA_A_α6Rs

**Fig. 6.**
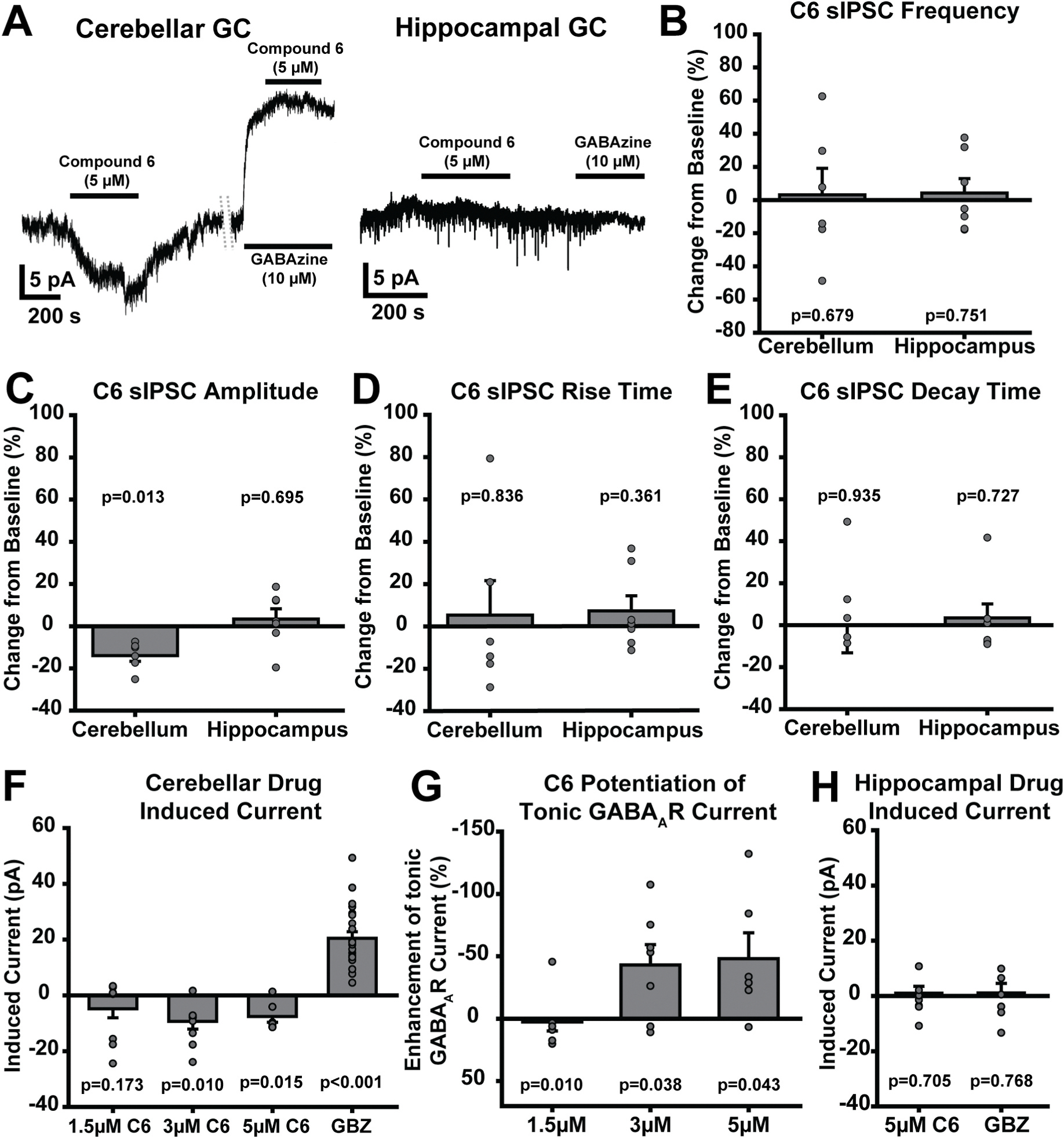
PZ-II-029, Compound 6, is a novel GABA_A_R positive allosteric modulator that is selective for GC GABA_A_45;6Rs. A. Example traces of cerebellar (left) and hippocampal (right) GC responses to 5 μM PZ-II-029 (Compound 6; C6) and 10 μM GABAzine (GBZ). **B-E.** Mean percent change in sIPSC frequency (**B**), amplitude (**C**), rise time (**D**), and decay time (**E**) from baseline after 5 μM C6 application in cerebellar (left) and hippocampal (right) GCs. **F.** Mean drug induced current from 1.5 μM, 3 μM, and 5 μM C6 as well as 10 μM GABAzine in cerebellar GCs. Note, reapplication of C6 in the presence of GBZ does not induce any detectable current, demonstrating that the current induced by C6 is a potentiation of the GC tonic GABA_A_R current generated by their extrasynaptic a6/d subunit-containing GABA_A_Rs **G.** Mean percent enhancement of the GC tonic GABA_A_R current by C6, quantified as the C6-induced current divided by the GBZ-induced current in each cell. **H.** Average drug-induced current from 5 μM C6 and 10 μM GBZ in hippocampal GCs. Data were analyzed using One Sample t-tests. **B.** Cerebellum: t(5) = 0.439, *p* = 0.679, Hippocampus: t(6) = −0.332, *p* = 0.751. **C.** Cerebellum: t(6) = −3.791, *p* = 0.013, Hippocampus: t(7) = 0.412, *p* = 0.695. **D.** Cerebellum: t(6) = −0.218, *p* = 0.836, Hippocampus: t(7) = −0.989, *p* = 0.361. **E.** Cerebellum: t(6) = 0.085, *p* = 0.935; Hippocampus: t(7) = −0.366, *p* = 0.726. **F.** 1.5 μM: t(9) = 1.482, *p* = 0.173, 3 μM: t(8) = 3.380, *p* = 0.010, 5 μM: t(6) = 3.628, *p* = 0.015, GBZ: t(25) = −8.746, *p* < 0.001. **H.** 5 μM t(7) = 0.396, *p* = 0.705, GBZ: t(7) = 0.311, *p* = 0.768. **G.** 1.5 μM: t(8) = −0.819, *p* = 0.436, Mann-Whitney Rank Sum: T(8,8) = 92, p = 0.010, 3 μM: t(8) = 3.380, *p* = 0.038, 5 μM: t(6) = 3.628, *p* = 0.043.

To further assess selectively targeting cerebellar GCs as a treatment for EtOH withdrawal symptoms, we tested PZ-II-029 (Compound 6; C6), a novel GABA_A_α6-selective positive allosteric modulator (Divović Matović et al., 2022; Varagic et al., 2013). The GABA_A_α6 receptor is exclusively found in cerebellar GCs (Kato, 1990) enabling us to selectively target cerebellar GCs without directly altering off target brain regions, and without the need for genetic modification, making it promising for translational studies. Before using this novel compound *in vivo*, we confirmed its functional effects and selectivity *ex vivo*, by examining responses in cerebellar GCs and in hippocampal GCs respectively, the latter of which express evolutionarily and pharmacologically similar GABA_A_α4 receptor subunits but, lack GABA_A_α6 subunits (Olsen and Sieghart, 2009; Sun et al., 2004) (Fig. 6). Bath application of Compound 6 (up to 5 mM) did not affect sIPSC frequency in the cerebellum (*p* = 0.679) or the hippocampus (*p* = 0.751) (Fig. 6B), nor did it affect sIPSC rise time (Fig. 6D; cerebellum: *p* = 0.836; hippocampus: *p* = 0.361) or decay time (Fig. 6E; cerebellum: *p* = 0.935; hippocampus: *p* = 0.726) in either brain region. Compound 6 did cause a small, but significant decrease in the amplitude of sIPSCs in cerebellar GCs (*p* = 0.013) but not hippocampal GCs (*p* = 0.695) (Fig. 6C). Importantly, Compound 6 significantly enhanced the magnitude of the cerebellar GC tonic GABA_A_R current at 3 μM (*p* = 0.010) and 5 μM (*p* = 0.015; Fig. 6A and F&G). This current enhancement was absent in the presence of the GABA_A_R antagonist GABAzine (GBZ, 10 mM; Fig. 6A), confirming that the current induced under control conditions is an enhancement of the tonic GABA_A_R current. Overall, Compound 6 increased cerebellar tonic inhibition by approximately 50 % based on the ratio of the magnitude of the Compound 6- and GBZ-induced current (3 μM: *p* = 0.038; 5 μM: *p* = 0.043; Fig. 6G). In contrast, hippocampal GCs showed no measurable current response to Compound 6 (*p* = 0.705; Fig. 6A and H). Thus, our results are consistent with its selective profile previously established in recombinant GABA_A_R subunits (Divović Matović et al., 2022; Varagic et al., 2013).

### 3.7. Compound 6 administration during withdrawal recues emotional but not motoric symptoms of EtOH withdrawal

**Fig. 7.**
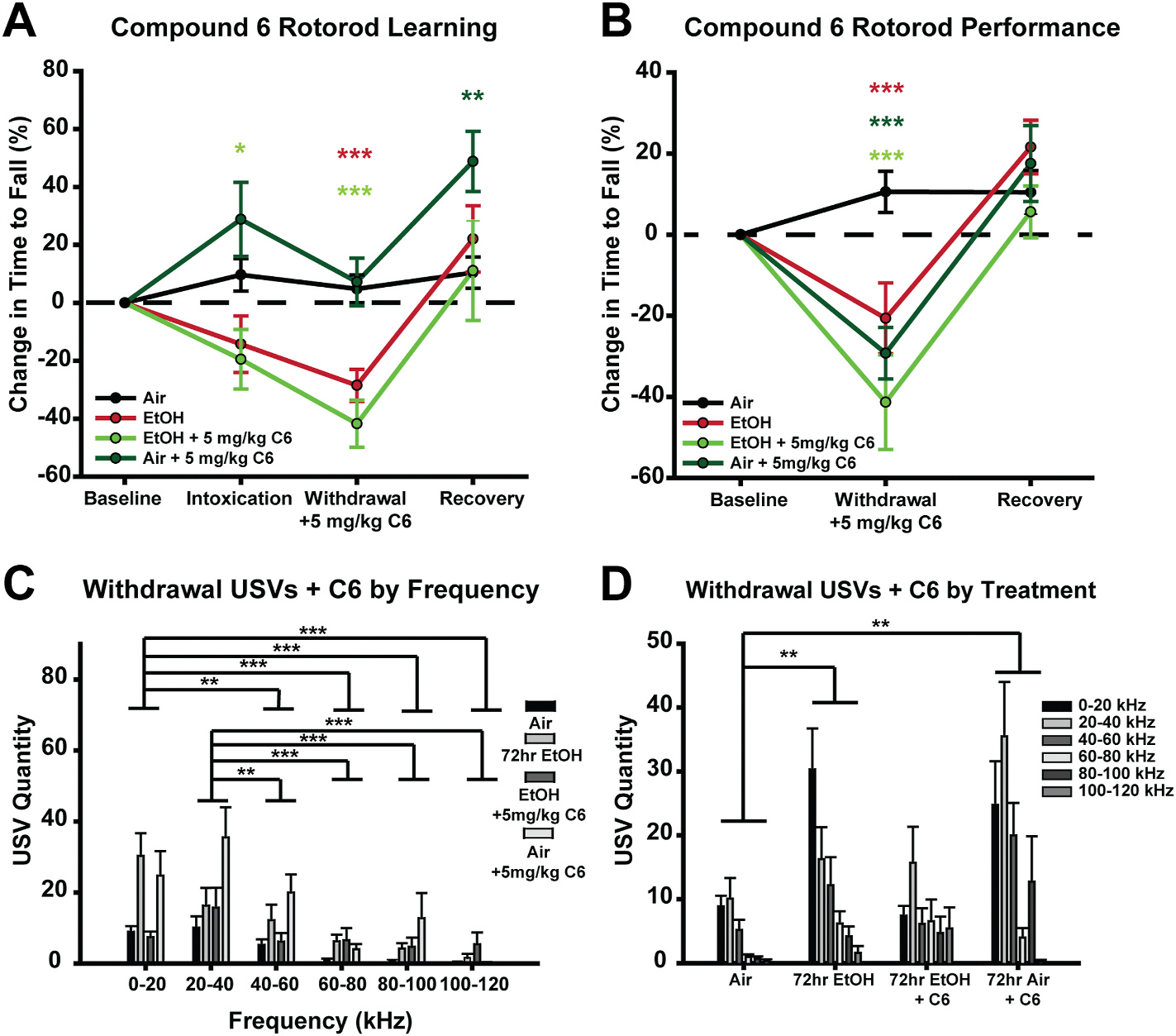
Compound 6 administration during withdrawal recues emotional but not motoric symptoms of EtOH withdrawal. **A.** Comparison in rotorod learning (first three trials per session standardized to baseline performance) during intoxication, withdrawal with or without 5 mg/kg C6, and recovery. **B.** Comparison in rotorod performance (last four trials per session standardized to baseline performance) during withdrawal with or without 5 mg/kg C6 and recovery. Color coded asterisks indicate a difference compared to untreated air controls. **C&D.** Mean USV quantity clustered by frequency (kHz) (**C**) and treatment condition (**D**) ± standard error. Data were analyzed using Mixed Factorial ANOVAs with Sidak post hoc comparisons. **A.** Treatment × Sex × Session: F_(9,72)_ = 1.166, *p* = 0.338, Treatment × Sex: F_(3,24)_ = 1.737, *p* = 0.186, Treatment × Session: F_(9,72)_ = 4.532, *p* < 0.001, Sex × Session: F_(3,72)_ = 1.000, *p* = 0.382, Treatment: F_(3,24)_ = 5.158, *p* = 0.007, Session: F_(3,72)_ = 17.654, *p* < 0.001, Sex: F_(1,24)_ = 0.011, *p* = 0.917. **B.** Treatment × Sex × Session: F_(6,48)_ = 1.262, *p* = 0.292, Treatment × Sex: F_(3,24)_ = 1.321, *p* = 0.291, Treatment × Session: F_(6,48)_ = 5.549, *p* < 0.001, Sex × Session: F_(2,48)_ = 2.111, *p* = 0.132, Treatment: F_(3,34)_ = 3.251, *p* = 0.039, Session: F_(3,24)_ = 40.214, *p* < 0.001, Sex: F_(1,44)_ = 3.279, *p* = 0.083. **G-H.** Treatment × Sex × Frequency: F_(15,115)_ = 1.063, *p* = 0.400, Treatment × Sex: F_(3,23)_ = 2.043, *p* = 0.136, Treatment × Frequency: F_(15,115)_ = 2.815, *p* = 0.013, Sex × Frequency: F_(5,115)_ = 0.564, *p* = 0.604, Treatment: F_(15,115)_ = 2.308, *p* = 0.103, Frequency: F_(5,115)_ = 16.378, *p* < 0.001, Sex: F_(1,23)_ = 6.818, *p* = 0.016. (*p < 0.05, **p < 0.005, ***p < 0.001). (*p < 0.05, **p < 0.005, ***p < 0.001).

Having validated Compound 6 as a cerebellar GC selective pharmacotherapeutic, we next tested its effectiveness in alleviating motor impairment and negative emotional affect during withdrawal from 72 h of EtOH/air vapor (Fig. 7). Surprisingly, Compound 6 did not improve motor impairment during withdrawal, with both untreated EtOH withdrawn mice and EtOH withdrawn mice treated with 5 mg/kg Compound 6 having significantly worse rotorod learning (*p* < 0.001) and performance (*p* < 0.001) compared to air controls during withdrawal (Fig. 7A and B). Thus, selectively enhancing cerebellar GC tonic GABA_A_R current does not improve motor discoordination during withdrawal (Fig. 7A and B). In contrast, Compound 6 significantly reduced low frequency USVs during withdrawal (Fig. 7C and D). Specifically, Compound 6 significantly reduced the quantity of USVs in EtOH withdrawn mice making them no different than untreated air controls (*p* = 0.137; Fig. 7C and D). Interestingly, Compound 6 administration in air withdrawn mice significantly increases the total number of USVs compared to untreated air controls (*p* = 0.001) suggesting that Compound 6 may be aversive when not used to treat withdrawal. Together, the data suggest that selective enhancement of cerebellar GC tonic GABA_A_R current with Compound 6 reduces negative emotional affect associated with EtOH withdrawal but does not alleviate withdrawal-induced motor impairment.

## 4. Discussion

Despite existing psychological and pharmacological treatment options for AUD, less than 20 % of individuals with AUD successfully maintain abstinence (SAMHSA, 2021). Therefore, it is a biomedical priority to identify novel treatment strategies to treat AUD symptoms. The cerebellum is an understudied structure for understanding the acquisition and maintenance of AUD, despite its clear involvement in acute responses to low concentrations of EtOH (Erikson et al., 2022; Otis et al., 2005; Roberto et al., 2006), and likely contributions to motoric/somatic actions of acute EtOH and EtOH withdrawal (Dar, 2015; Otis et al., 2005; Philibin et al., 2008). Moreover, increasing literature suggests that the cerebellum plays critical roles in cognitive, emotional, and reward processing (Carta Ilaria et al., 2019; Middleton and Strick, 1994; Schmahmann and Pandya, 1997; Stoodley et al., 2012), which would therefore also be affected by acute and withdrawal-induced changes in cerebellar physiology. Thus, the cerebellum is an understudied but clearly important structure to characterize and target in the context of AUD related neurological mechanisms.

In this study, we investigated the role of cerebellar GCs in mediating EtOH withdrawal-induced motor impairment and negative affect, critical drivers of the AUD cycle. We found that the cerebellum exhibits a homeostatic downregulation of GC inhibitory tone, via a decrease in sIPSC frequency that first emerges after 48 h of EtOH exposure (Fig. 1). We emphasize that this is a homeostatic adaptation, given extensive past work showing that the acute actions of EtOH on cerebellar GCs is to increase GABAergic sIPSC frequency and the magnitude of their tonic GABA_A_R current in varying proportions depending on species (Carta et al., 2004; Erikson et al., 2022; Jin et al., 2021; J. Kaplan et al., 2013). Importantly, the observed reduction in sIPSC frequency coincides with the onset of withdrawal-induced motor impairment (assayed with rotorod learning and performance) and negative emotional affect (assayed with USVs and blood CORT levels) as aversive withdrawal symptoms (Figs. 2 and 3). While the rotorod assay is commonly used to assess motor coordination impairments (Lalonde et al., 1995) and is established as an aversive EtOH withdrawal symptom (Philibin et al., 2008), USVs are less established as a readout of emotional affect in mice. However, while there are numerous alternative assays of negative affect, many of them rely heavily on intact motor performance (Gencturk and Unal, 2024). To circumvent this confound, we focused on USVs, a less motor dependent measure of negative affect, by adapting protocols from EtOH withdrawal in rats (Barker et al., 2015) and other aversive states in mice (Lahvis et al., 2011; Premoli et al., 2021). We found that EtOH withdrawn mice elicit significantly more low-frequency USVs compared to their air withdrawn counterparts, consistent with the aforementioned studies of negative emotionality in rats and mice. To confirm that these vocalizations reflect a stressful, negatively affective experience, we used CORT measurements as a physiological readout of stress (Fig. 3E). Strikingly, the rise in aversive low frequency USVs coincided with an increase in CORT levels during EtOH withdrawal, thereby temporally linking altered USVs with clear physiological stress during EtOH withdrawal.

To determine whether the correlation between adapted cerebellar synaptic physiology and behavioral withdrawal symptoms is causative, we used GC-selective chemogenetics and pharmacology to counteract cerebellar GC loss of inhibition during withdrawal. Specifically, activation of cerebellar GC-specific GiDs improved rotorod performance (Fig. 5D), but not USVs (Fig. 5E and F). In contrast, increasing cerebellar GC inhibitory tone with systemic Compound 6 treatment, which selectively enhances GC tonic GABA_A_R currents (Fig. 6), improved USVs (Fig. 7C and D), but failed to improve rotorod learning or performance during EtOH withdrawal (Fig. 7A and B). This discrepancy is consistent with previous studies showing that phasic and tonic inhibition of cerebellar GCs have divergent impacts on transmission through the cerebellar cortex (Crowley et al., 2009; Galliano et al., 2013; Hamann et al., 2002; Mapelli et al., 2014). Specifically, tonic inhibition, mediated predominantly by extrasynaptic GABA_A_Rs, constitute the dominant inhibitory mechanism regulating GC excitability, significantly exceeding the inhibitory mechanisms provided by phasic (synaptic) currents even at high-frequency stimulation (Hamann et al., 2002). However, phasic sIPSCs may play a more important role in timing and/or threshold, while tonic GABA_A_R currents modulate gain (Crowley et al., 2009; Mapelli et al., 2014). Thus, selectively enhancing tonic inhibition might preferentially modulate affective withdrawal symptoms that require less temporal precision, without alleviating motor deficits that are more dependent on precise timing. The converse may be true for GiD inhibition of GC axonal output (Campbell and Marchant, 2018), highlighting the complexity and specificity of cerebellar inhibitory control during EtOH withdrawal. Collectively, our findings indicate that increasing GC inhibitory tone alleviates both somatic (motor) and affective (emotional) aversive withdrawal symptoms, but that the potential for pharmacological recovery involves separable aspects of GC physiology.

Having established the role of the cerebellum in both somatic and affective withdrawal symptoms, we wanted to determine if this role could contribute to the well-established negative reinforcement drive for renewed EtOH consumption. Indeed, restoring GC inhibitory tone through acute EtOH administration during withdrawal *in vivo* improved both motor coordination and negative affect (Fig. 4). Although the restoration of rotorod performance was incomplete, when comparing motor performance after acute EtOH injection, performance is significantly better in EtOH withdrawn mice compared to air exposed controls (Fig. 4B), consistent with the development of functional tolerance, which along with alleviation of aversive withdrawal symptoms, is an important component of the AUD cycle (Abrahao et al., 2017; Koob and Volkow, 2010).

There are a couple of aspects to our study that are potential limitations when compared to current dominant methodologies. First, we utilize an uncommonly used low EtOH consuming mouse strain for our AUD studies. However, AUD is only considered to be 50–60 % genetically driven (Hill et al., 2010). Thus, the low EtOH consuming B6N mouse strain studied here is likely reflective of the remaining 40–50 % of the AUD population that is not genetically predisposed. Indeed, escalation of EtOH consumption does develop in another low EtOH consuming rodent genotype (DBA/2J mice) when access is repeatedly given during peak withdrawal from chronic forced EtOH consumption (Fidler et al., 2011). In this context, we have established that such low EtOH consuming B6N mice exhibit two of the hallmarks of EtOH dependence: aversive withdrawal symptoms and tolerance to EtOH, which are generally accepted as components of negative reinforcement for renewed EtOH consumption.

Secondly, while the 72-h chronic vapor model is well established to induce homeostatic molecular adaptations that contribute to withdrawal and likely dependence, it could be argued that 72 h straight of high EtOH vapor exposure is not reflective of the human experience, and thus a concern, even if it does effectively model withdrawal. In this regard, we’ve established that most of the adaptations that occur in the cerebellum of B6N mice at 72 h, already occur after only 48 h, making it less conceptually complicated. But most importantly, a survey of college students indicates that it is common among a subset of students to drink heavily from Thursday through Sunday, including 25 % who consume 4–5 units per day, ~5 % that report being stumbling drunk per day, and ~4 % that report being black out drunk per day (Maggs et al., 2011). Combining that documented behavior with the much slower metabolism of EtOH by humans compared to mice suggests that the 48–72 h vapor model in mice may actually be reflective of a subset of people, presumably not all of whom are genetically predisposed to develop AUD, but do begin to learn the negatively reinforcing value of EtOH.

While cerebellar involvement in motor aspects of EtOH withdrawal is not surprising, it is worth emphasizing that both acute EtOH and cerebellar specific positive allosteric modulator, Compound 6, fully rescue USVs indicative of negative affect (Figs. 4D and 7D, respectively), suggesting a dominant role for the cerebellum in EtOH-withdrawal induced negative affect as well. However, numerous previous studies have demonstrated similarly critical roles of disparate brain regions in withdrawal-induced negative affect, including to name just a few, extended amygdala (Centanni et al., 2019), prefrontal cortex (Patel et al., 2022), and striatum (Borrego et al., 2022; Lepreux et al., 2024), which raises the question of how these disparate nuclei interact, and/or whether the relative importance of each brain region is dependent on exposure model and strain of mammal used.

In summary, our data establish that the cerebellum plays a strong role in mediating somatic and affective withdrawal symptoms in an acute withdrawal paradigm that models early binge like EtOH consumption by humans that do not have genetic risk for AUD, which represents a significant proportion of the human population that develop AUD (Boness et al., 2016; Esser et al., 2014; Swift and Davidson, 1998). However, it will require further research to determine if the cerebellar adaptations we report here, and their role in tolerance and aversive withdrawal symptoms, also occur in high EtOH consuming genotypes and other models of EtOH withdrawal.

## Supplementary Material

1

## Data Availability

Data will be made available on request.

## Appendix A. Supplementary data

Supplementary data to this article can be found online at https://doi.org/10.1016/j.neuropharm.2025.110595.
