# Supplementary material for: Selectively counteracting cerebellar adaptations to chronic alcohol exposure reduces acute alcohol withdrawal severity in C57BL6/N mice
Source: Neuropharmacology. Author manuscript; Available in PMC 2026 Apr 22. (PMC13102386; doi:10.1016/j.neuropharm.2025.110595)
Supplement: 1 [file NIHMS2157666-supplement-1.pdf]

Updated 6/30/25

Bold indicates stats directly stated in the manuscript/figure legends

	Figure #	Figure Letter	Animal #	Cell #	normality	Test Type	DF	F- / T- / Z-Value	Critical Level	P-value	Significant?	Comments
Anterior vs Posterior Lobule Breakdown						Anterior vs Posterior Lobule Breakdown						
			23	71	No	2-Way ANOVA	3,68	F = 4.963	0.05	0.004	Yes	sIPSC Frequency by EtOH Duration-The difference in the mean values among the different levels of EtOH Duration is greater than would be expected by chance after allowing for effects of differences in Lobule Location
						2-Way ANOVA	1,68	F = 0.109	0.05	0.742	No	sIPSC Frequency by Lobule Location-NO MAIN EFFECT
						2-Way ANOVA	3,68	F = 1.110	0.05	0.352	No	sIPSC Frequency by EtOH Duration x Lobule Location- NO INTERACTION
						**Kruskal-Wallis Analysis on Ranks**	**7**	**H = 11.421**	**0.05**	**0.121**	**No**	**sIPSC Frequency by EtOH Duration-NO CHANGE**
			23	71	Yes	2-Way ANOVA	3,66	F = 1.051	0.05	0.377	No	sIPSC Amplitude by EtOH Duration-NO MAIN EFFECT
						**2-Way ANOVA**	**1,66**	**F = 0.374**	**0.05**	**0.543**	**No**	**sIPSC Amplitude by Lobule Location-NO MAIN EFFECT**
						2-Way ANOVA	3,66	F = 1.036	0.05	0.383	No	sIPSC Amplitude by EtOH Duration x Lobule Location- NO INTERACTION
			23	71	No	2-Way ANOVA	3,61	F = 1.077	0.05	0.367	No	Tonic GABAAR Current by EtOH Duration-NO MAIN EFFECT
						2-Way ANOVA	1,61	F = 0.303	0.05	0.585	No	Tonic GABAAR Current by Lobule Location-NO MAIN EFFECT
						2-Way ANOVA	3,61	F = 0.354	0.05	0.786	No	Tonic GABAAR Current by EtOH Duration x Lobule Location- NO INTERACTION
						**Kruskal-Wallis Analysis on Ranks**	**7**	**H = 5.983**	**0.05**	**0.542**	**No**	**Tonic GABAAR Current by EtOH Duration-NO CHANGE**
sIPSC Frequency	1	B	23	78	No	2-way (2 x 3) ANOVA	1, 70	F = 0.787	0.050	0.378	No	Sex - NO MAIN EFFECT
						2-way (2 x 3) ANOVA	3, 70	F = 5.101	0.050	0.003	Yes	EtOH Duration - MAIN EFFECT: The difference in the mean values among the different levels of EtOH Duration is greater
						2-way (2 x 3) ANOVA	3, 70	F = 0.735	0.050	0.735	No	Sex v EtOH Duration - NO INTERACTION
						**All Pairwise Multiple Comparison Procedures (Holm-Sidak method)**	**-**	**t = 3.366**	**0.009**	**0.001**	**Yes**	**Air vs 48 Hour EtOH in EtOH Duration**
						**All Pairwise Multiple Comparison Procedures (Holm-Sidak method)**	**-**	**t = 3.145**	**0.010**	**0.002**	**Yes**	**Air vs 72 Hour EtOH in EtOH Duration**
						All Pairwise Multiple Comparison Procedures (Holm-Sidak method)	-	t = 2.164	0.013	0.034	No	24 Hr EtOH vs 48 Hour EtOH in EtOH Duration
						All Pairwise Multiple Comparison Procedures (Holm-Sidak method)	-	t = 1.823	0.017	0.073	No	24 Hr EtOH vs 72 Hour EtOH in EtOH Duration
						**All Pairwise Multiple Comparison Procedures (Holm-Sidak method)**	**-**	**t = 1.360**	**0.025**	**0.178**	**No**	**Air vs 24 Hour EtOH in EtOH Duration**
						All Pairwise Multiple Comparison Procedures (Holm-Sidak method)	-	t = 0.523	0.050	0.603	No	72 Hr EtOH vs 48 Hour EtOH in EtOH Duration
						**Kruskal-Wallis Analysis on Ranks**	**3**	**H = 12.538**	**0.05**	**0.006**	**Yes**	**sIPSC Frequency: SIGNIFICANT EFFECT**
							Diff of Ranks		Q		P<0.05	
						**Dunn's Comparison**	**25.191**		**3.235**		**Yes**	**Air vs 48 hr etoh**
						**Dunn's Comparison**	**19.037**		**2.670**		**Yes**	**Air vs 72 hr etoh**
						**Dunn's Comparison**	**9.996**		**1.373**		**No**	**Air vs 24 hr etoh**
						Dunn's Comparison	15.195		2.021		No	24 hr etoh vs 48 hr etoh
						Dunn's Comparison	9.041		1.322		Do Not Test	24 hr etoh vs 72 hr etoh
						Dunn's Comparison	6.154		0.834		Do Not Test	72 hr etoh vs 48 hr etoh
sIPSC Amplitude	1	C	23	77	No	2-way (2 x 3) ANOVA	1, 69	F = 1.485	0.050	0.227	No	Sex - NO MAIN EFFECT
						**2-way (2 x 3) ANOVA**	**3, 69**	**F = 0.685**	**0.050**	**0.564**	**No**	**EtOH Duration - NO MAIN EFFECT**
						2-way (2 x 3) ANOVA	3, 69	F = 0.645	0.050	0.589	No	Sex v EtOH Duration - NO INTERACTION
						**Kruskal-Wallis Analysis on Ranks**	**3**	**H = 1.698**	**0.05**	**0.637**	**No**	**sIPSC Amplitude: NO CHANGE**
Basal Holding Current	1	D	23	77	No	2-way (2 x 3) ANOVA	1, 69	F = 3.142	0.050	0.081	No	Sex - NO MAIN EFFECT
						**2-way (2 x 3) ANOVA**	**3, 69**	**F = 0.817**	**0.050**	**0.489**	**No**	**EtOH Duration - NO MAIN EFFECT**
						2-way (2 x 3) ANOVA	3, 69	F = 0.838	0.050	0.478	No	Sex v EtOH Duration - NO INTERACTION
						**Kruskal-Wallis Analysis on Ranks**	**3**	**H = 3.684**	**0.05**	**0.298**	**No**	**Basal Holding Current: NO CHANGE**

Tonic $GABA_A$ Current	1	D	23	70	No	**2-way (2 x 3) ANOVA**	**1, 62**	**F = 4.074**	**0.050**	**0.048**	**Yes**	**Sex - MAIN EFFECT: The difference in the mean values among the different levels of Sex is greater than would be expected by chance after allowing for effects of differences in EtOH Duration.**
						2-way (2 x 3) ANOVA	3, 62	F = 1.182	0.050	0.324	No	EtOH Duration - NO MAIN EFFECT
						**2-way (2 x 3) ANOVA**	**3, 62**	**F = 1.231**	**0.050**	**0.306**	**No**	**Sex v EtOH Duration - NO INTERACTION**
						**All Pairwise Multiple Comparison Procedures (Holm-Sidak method)**	**-**	**t = 2.018**	**0.050**	**0.048**	**Yes**	**Male vs Female in Sex**
						**Kruskal-Wallis Analysis on Ranks**	**3**	**H = 2.970**	**0.05**	**0.396**	**No**	**Tonic GABAAR Current by EtOH Duration: NO CHANGE**
						**Mann-Whitney U Stat**	**33;37**	**T= 1299**	**0.05**	**0.135**	**No**	**Tonic GABAAR Current by Sex: NO CHANGE**

	Figure #	Figure Letter	Animal #	Cell #	normality	Test Type	DF	F- / T- / Z-Value	Critical Level	P-value	Significant?	Comments
						Mixed Factorial ANOVA	1, 54	F = 4.898	0.050	0.031	Yes	Sex -MAIN EFFECT: Main effects cannot be properly interpreted if significant interaction is determined. See post hoc comparisons.
						**Mixed Factorial ANOVA**	**6, 324**	**F = 29.724**	**0.050**	**< 0.001**	**Yes**	**Trial - MAIN EFFECT: Main effects cannot be properly interpreted if significant interaction is determined. See post hoc comparisons.**
						**Mixed Factorial ANOVA**	**1, 324**	**F = 179.953**	**0.050**	**< 0.001**	**Yes**	**Training Day - MAIN EFFECT: Main effects cannot be properly interpreted if significant interaction is determined. See post hoc comparisons.**
						Mixed Factorial ANOVA	6, 324	F = 1.014	0.050	0.410	No	Trial vs Sex - NO INTERACTION
						Mixed Factorial ANOVA	1, 324	F = 0.372	0.050	0.545	No	Training Day vs Sex - NO INTERACTION
						Mixed Factorial ANOVA	6, 324	F = 9.589	0.050	< 0.001	Yes	Trial vs Training Day - INTERACTION: The effect of different levels of Trial depends on what level of Session is present. There is a statistically significant interaction between Trial and Training Day.
						Mixed Factorial ANOVA	6, 324	F = 0.942	0.050	0.465	No	Trail vs Training Day vs Sex - NO INTERACTION
						All Pairwise Multiple Comparison Procedures (Holm-Sidak method)	-	t = 11.952	0.050	< 0.001	Yes	Training Day 1 vs Training Day 6 in Trial 1
						All Pairwise Multiple Comparison Procedures (Holm-Sidak method)	-	t = 12.229	0.050	< 0.001	Yes	Training Day 1 vs Training Day 6 in Trial 2
						All Pairwise Multiple Comparison Procedures (Holm-Sidak method)	-	t = 12.235	0.050	< 0.001	Yes	Training Day 1 vs Training Day 6 in Trial 3
						All Pairwise Multiple Comparison Procedures (Holm-Sidak method)	-	t = 9.825	0.050	< 0.001	Yes	Training Day 1 vs Training Day 6 in Trial 4
						All Pairwise Multiple Comparison Procedures (Holm-Sidak method)	-	t = 8.615	0.050	< 0.001	Yes	Training Day 1 vs Training Day 6 in Trial 5
						All Pairwise Multiple Comparison Procedures (Holm-Sidak method)	-	t = 7.241	0.050	< 0.001	Yes	Training Day 1 vs Training Day 6 in Trial 6
						All Pairwise Multiple Comparison Procedures (Holm-Sidak method)	-	t = 7.219	0.050	< 0.001	Yes	Training Day 1 vs Training Day 6 in Trial 7
						All Pairwise Multiple Comparison Procedures (Holm-Sidak method)	-	t = 10.635	0.002	< 0.001	Yes	Trial 1 vs Trial 7 in Training Day 1
						All Pairwise Multiple Comparison Procedures (Holm-Sidak method)	-	t = 10.176	0.003	< 0.001	Yes	Trial 1 vs Trial 6 in Training Day 1
						All Pairwise Multiple Comparison Procedures (Holm-Sidak method)	-	t = 9.335	0.003	< 0.001	Yes	Trial 1 vs Trial 5 in Training Day 1
						All Pairwise Multiple Comparison Procedures (Holm-Sidak method)	-	t = 8.162	0.003	< 0.001	Yes	Trial 2 vs Trial 7 in Training Day 1
						All Pairwise Multiple Comparison Procedures (Holm-Sidak method)	-	t = 7.827	0.003	< 0.001	Yes	Trial 1 vs Trial 4 in Training Day 1
						All Pairwise Multiple Comparison Procedures (Holm-Sidak method)	-	t = 7.703	0.003	< 0.001	Yes	Trial 2 vs Trial 6 in Training Day 1
						All Pairwise Multiple Comparison Procedures (Holm-Sidak method)	-	t = 6.862	0.003	< 0.001	Yes	Trial 2 vs Trial 5 in Training Day 1
						All Pairwise Multiple Comparison Procedures (Holm-Sidak method)	-	t = 5.671	0.004	< 0.001	Yes	Trial 3 vs Trial 7 in Training Day 1

Baseline Rotorod Performance	2	A		56		Assumed N≥30	All Pairwise Multiple Comparison Procedures (Holm-Sidak method)	-	t = 5.354	0.004	< 0.001	Yes	Trial 2 vs Trial 4 in Training Day 1
							All Pairwise Multiple Comparison Procedures (Holm-Sidak method)	-	t = 5.213	0.004	< 0.001	Yes	Trial 3 vs Trial 6 in Training Day 1
							All Pairwise Multiple Comparison Procedures (Holm-Sidak method)	-	t = 4.963	0.005	< 0.001	Yes	Trial 1 vs Trial 3 in Training Day 1
							All Pairwise Multiple Comparison Procedures (Holm-Sidak method)		t = 4.372	0.005	< 0.001	Yes	Trial 3 vs Trial 5 in Training Day 1
							All Pairwise Multiple Comparison Procedures (Holm-Sidak method)		t = 2.864	0.006	0.004	Yes	Trial 3 vs Trial 4 in Training Day 1
							All Pairwise Multiple Comparison Procedures (Holm-Sidak method)		t = 2.807	0.006	0.005	Yes	Trial 4 vs Trial 7 in Training Day 1
							All Pairwise Multiple Comparison Procedures (Holm-Sidak method)		t = 2.490	0.007	0.013	No	Trial 2 vs Trial 3 in Training Day 1
							All Pairwise Multiple Comparison Procedures (Holm-Sidak method)		t = 2.473	0.009	0.014	No	Trial 1 vs Trial 2 in Training Day 1
							All Pairwise Multiple Comparison Procedures (Holm-Sidak method)		t = 2.349	0.010	0.019	No	Trial 4 vs Trial 6 in Training Day 1
							All Pairwise Multiple Comparison Procedures (Holm-Sidak method)		t = 1.508	0.013	0.132	No	Trial 4 vs Trial 5 in Training Day 1
							All Pairwise Multiple Comparison Procedures (Holm-Sidak method)		t = 1.300	0.017	0.194	No	Trial 5 vs Trial 7 in Training Day 1
							All Pairwise Multiple Comparison Procedures (Holm-Sidak method)		t = 0.841	0.025	0.401	No	Trial 5 vs Trial 6 in Training Day 1
							All Pairwise Multiple Comparison Procedures (Holm-Sidak method)		t = 0.459	0.05	0.647	No	Trial 6 vs Trial 7 in Training Day 1
							All Pairwise Multiple Comparison Procedures (Holm-Sidak method)	-	t = 5.336	0.002	< 0.001	Yes	Trial 1 vs Trial 3 in Training Day 6
							All Pairwise Multiple Comparison Procedures (Holm-Sidak method)	-	t = 5.033	0.003	< 0.001	Yes	Trial 1 vs Trial 4 in Training Day 6
							All Pairwise Multiple Comparison Procedures (Holm-Sidak method)	-	t = 4.951	0.003	< 0.001	Yes	Trial 1 vs Trial 5 in Training Day 6
							All Pairwise Multiple Comparison Procedures (Holm-Sidak method)	-	t = 4.417	0.003	< 0.001	Yes	Trial 1 vs Trial 7 in Training Day 6
							All Pairwise Multiple Comparison Procedures (Holm-Sidak method)	-	t = 3.987	0.003	< 0.001	Yes	Trial 1 vs Trial 6 in Training Day 6
							All Pairwise Multiple Comparison Procedures (Holm-Sidak method)	-	t = 2.838	0.003	0.005	No	Trial 1 vs Trial 2 in Training Day 6
							All Pairwise Multiple Comparison Procedures (Holm-Sidak method)	-	t = 2.497	0.003	0.013	No	Trial 2 vs Trial 3 in Training Day 6
							All Pairwise Multiple Comparison Procedures (Holm-Sidak method)	-	t = 2.195	0.004	0.029	No	Trial 2 vs Trial 4 in Training Day 6
							All Pairwise Multiple Comparison Procedures (Holm-Sidak method)	-	t = 2.113	0.004	0.035	No	Trial 2 vs Trial 5 in Training Day 6
							All Pairwise Multiple Comparison Procedures (Holm-Sidak method)	-	t = 1.579	0.004	0.115	No	Trial 2 vs Trial 7 in Training Day 6
							All Pairwise Multiple Comparison Procedures (Holm-Sidak method)	-	t = 1.348	0.005	0.178	No	Trial 3 vs Trial 6 in Training Day 6
							All Pairwise Multiple Comparison Procedures (Holm-Sidak method)	-	t = 1.149	0.005	0.251	No	Trial 2 vs Trial 6 in Training Day 6
							All Pairwise Multiple Comparison Procedures (Holm-Sidak method)	-	t = 1.046	0.006	0.296	No	Trial 4 vs Trial 6 in Training Day 6
							All Pairwise Multiple Comparison Procedures (Holm-Sidak method)	-	t = 0.964	0.006	0.336	No	Trial 5 vs Trial 6 in Training Day 6
							All Pairwise Multiple Comparison Procedures (Holm-Sidak method)	-	t = 0.919	0.007	0.358	No	Trial 3 vs Trial 7 in Training Day 6
							All Pairwise Multiple Comparison Procedures (Holm-Sidak method)	-	t = 0.616	0.009	0.538	No	Trial 4 vs Trial 7 in Training Day 6

					All Pairwise Multiple Comparison Procedures (Holm-Sidak method)	-	t = 0.534	0.010	0.593	No	Trial 5 vs Trial 7 in Training Day 6
					All Pairwise Multiple Comparison Procedures (Holm-Sidak method)	-	t = 0.429	0.013	0.668	No	Trial 6 vs Trial 7 in Training Day 6
					All Pairwise Multiple Comparison Procedures (Holm-Sidak method)	-	t = 0.384	0.017	0.701	No	Trial 3 vs Trial 5 in Training Day 6
					All Pairwise Multiple Comparison Procedures (Holm-Sidak method)	-	t = 0.303	0.025	0.762	No	Trial 3 vs Trial 4 in Training Day 6
					All Pairwise Multiple Comparison Procedures (Holm-Sidak method)	-	t = 0.0819	0.05	0.935	No	Trial 4 vs Trial 5 in Training Day 6
**Figure #**	**Figure Letter**	**Animal #**	**Cell #**	**normality**	**Test Type**	**DF**	**F- / T- / Z-Value**	**Critical Level**	**P-value**	**Significant?**	**Comments**
					24, 48, 72 Hour Air Control-Rotorod Learning						
					2-Way RM ANOVA	2	F = 0.387	0.05	0.683	No	Air Duration- NO MAIN EFFECT
					2-Way RM ANOVA	3	F = 1.498	0.05	0.223	No	Trial-NO MAIN EFFECT
					**2-Way RM ANOVA**	**6**	**F = 0.484**	**0.05**	**0.818**	**No**	**Air Duration x Trial- NO INTERACTION**
					**Mixed Factor RM ANOVA**	**3,48**	**F = 5.072**	**0.050**	**0.004**	**Yes**	**EtOH Duration-MAIN EFFECT: Main effects cannot be properly interpreted if significant interaction is determined. See post hoc comparisons.**
					**Mixed Factor RM ANOVA**	**3,144**	**F = 24.809**	**0.050**	**< 0.001**	**Yes**	**Trial-MAIN EFFECT: Main effects cannot be properly interpreted if significant interaction is determined. See post hoc comparisons.**
					**Mixed Factor RM ANOVA**	**1,48**	**F = 3.471**	**0.050**	**0.069**	**No**	**Sex-NO MAIN EFFECT**
					Mixed Factor RM ANOVA	3,144	F = 1.195	0.050	0.312	No	Trial x Sex- NO INTERACTION
					**Mixed Factor RM ANOVA**	**3,144**	**F = 6.231**	**0.050**	**< 0.001**	**Yes**	**Trial x EtOH Duration-INTERACTION: The effect of different levels of Trial depends on what level of EtOH Duration is present. There is a statistically significant interaction between EtOH Duration and Trial**
					Mixed Factor RM ANOVA	3,48	F = 0.427	0.050	0.073	No	EtOH Duration x Sex-NO INTERACTION
					Mixed Factor RM ANOVA	9,144	F = 0.809	0.050	0.593	No	Trial x Sex x EtOH Duration- NO INTERACTION
					All Pairwise Multiple Comparison Procedures (Holm-Sidak method)	-	t = 5.479	0.009	< 0.001	Yes	Withdrawal vs Recovery in 72 Hr EtOH
					All Pairwise Multiple Comparison Procedures (Holm-Sidak method)	-	t = 3.936	0.010	< 0.001	Yes	Intoxication vs Recovery in 72 Hr EtOH
					All Pairwise Multiple Comparison Procedures (Holm-Sidak method)	-	t = 3.089	0.013	0.002	Yes	Baseline vs Withdrawal in 72 Hr EtOH
					All Pairwise Multiple Comparison Procedures (Holm-Sidak method)	-	t = 2.390	0.017	0.018	No	Baseline vs Recovery in 72 Hr EtOH
					All Pairwise Multiple Comparison Procedures (Holm-Sidak method)	-	t = 1.546	0.025	0.124	No	Baseline vs Intoxication in 72 Hr EtOH
					All Pairwise Multiple Comparison Procedures (Holm-Sidak method)	-	t = 1.543	0.050	0.125	No	Intoxication vs Withdrawal in 72 Hr EtOH
					All Pairwise Multiple Comparison Procedures (Holm-Sidak method)	-	t = 1.642	0.009	0.103	No	Baseline vs Intoxication in Air
					All Pairwise Multiple Comparison Procedures (Holm-Sidak method)	-	t = 1.256	0.010	0.211	No	Intoxication vs Withdrawal in Air
					All Pairwise Multiple Comparison Procedures (Holm-Sidak method)	-	t = 1.233	0.013	0.219	No	Baseline vs Recovery in Air
					All Pairwise Multiple Comparison Procedures (Holm-Sidak method)	-	t = 0.847	0.017	0.398	No	Wihtdrawal vs Recovery in Air
					All Pairwise Multiple Comparison Procedures (Holm-Sidak method)	-	t = 0.409	0.025	0.683	No	Intoxication vs Recovery in Air
					All Pairwise Multiple Comparison Procedures (Holm-Sidak method)	-	t = 0.386	0.050	0.700	No	Baseline vs Withdrawal in Air

Rotorod Learning	2	B	56		Assumed N≥30	All Pairwise Multiple Comparison Procedures (Holm-Sidak method)	-	t = 3.258	0.009	0.001	Yes	Withdrawal vs Recovery in 48 Hr EtOH
						All Pairwise Multiple Comparison Procedures (Holm-Sidak method)	-	t = 2.854	0.010	0.005	Yes	Baseline vs Withdrawal in 48 Hr EtOH
						All Pairwise Multiple Comparison Procedures (Holm-Sidak method)	-	t = 1.966	0.013	0.051	No	Intoxication vs Recovery in 48 Hr EtOH
						All Pairwise Multiple Comparison Procedures (Holm-Sidak method)	-	t = 1.562	0.017	0.120	No	Baseline vs Intoxication in 48 Hr EtOH
						All Pairwise Multiple Comparison Procedures (Holm-Sidak method)	-	t = 1.292	0.025	0.198	No	Intoxication vs Withdrawal in 48 Hr EtOH
						All Pairwise Multiple Comparison Procedures (Holm-Sidak method)	-	t = 0.404	0.050	0.686	No	Baseline vs Recovery in 48 Hr EtOH
						All Pairwise Multiple Comparison Procedures (Holm-Sidak method)	-	t = 4.566	0.009	< 0.001	Yes	Intoxication vs Recovery in 24 Hr EtOH
						All Pairwise Multiple Comparison Procedures (Holm-Sidak method)	-	t = 4.335	0.010	< 0.001	Yes	Baseline vs Intoxication in 24 Hr EtOH
						All Pairwise Multiple Comparison Procedures (Holm-Sidak method)	-	t = 3.282	0.013	0.001	Yes	Withdrawal vs Recovery in 24 Hr EtOH
						All Pairwise Multiple Comparison Procedures (Holm-Sidak method)	-	t = 3.051	0.017	0.003	Yes	Baseline vs Withdrawal in 24 Hr EtOH
						All Pairwise Multiple Comparison Procedures (Holm-Sidak method)	-	t = 1.284	0.025	0.201	No	Intoxication vs Withdrawal in 24 Hr EtOH
						All Pairwise Multiple Comparison Procedures (Holm-	-	t = 0.231	0.050	0.818	No	Baseline vs Recovery in 24 Hr EtOH
						**All Pairwise Multiple Comparison Procedures (Holm-Sidak method)**	**-**	**t = 5.927**	**0.009**	**< 0.001**	**Yes**	**Air vs 24 Hr EtOH in Intoxication**
						**All Pairwise Multiple Comparison Procedures (Holm-Sidak method)**	**-**	**t = 2.857**	**0.010**	**0.005**	**Yes**	**Air vs 48 Hr EtOH in Intoxication**
						**All Pairwise Multiple Comparison Procedures (Holm-Sidak method)**	**-**	**t = 2.840**	**0.013**	**0.005**	**Yes**	**Air vs 72 Hr EtOH in Intoxication**
						**All Pairwise Multiple Comparison Procedures (Holm-Sidak method)**	**-**	**t = 2.569**	**0.017**	**0.011**	**Yes**	**72 Hr EtOH vs 24 Hr EtOH in Intoxication**
						**All Pairwise Multiple Comparison Procedures (Holm-Sidak method)**	**-**	**t = 2.555**	**0.025**	**0.011**	**Yes**	**48 Hr EtOH vs 24 Hr EtOH in Intoxication**
						All Pairwise Multiple Comparison Procedures (Holm-Sidak method)	-	t = 0.0143	0.050	0.989	No	72 Hr EtOH vs 48 Hr EtOH in Intoxication
						**All Pairwise Multiple Comparison Procedures (Holm-Sidak method)**	**-**	**t = 3.686**	**0.009**	**< 0.001**	**Yes**	**Air vs 72 Hr EtOH in Withdrawal**
						**All Pairwise Multiple Comparison Procedures (Holm-Sidak method)**	**-**	**t = 3.643**	**0.010**	**< 0.001**	**Yes**	**Air vs 24 Hr EtOH in Withdrawal**
						**All Pairwise Multiple Comparison Procedures (Holm-Sidak method)**	**-**	**t = 3.425**	**0.013**	**< 0.001**	**Yes**	**Air vs 48 Hr EtOH in Withdrawal**
						All Pairwise Multiple Comparison Procedures (Holm-Sidak method)	-	t = 0.217	0.017	0.829	No	48 Hr EtOH vs 72 Hr EtOH in Withdrawal
						All Pairwise Multiple Comparison Procedures (Holm-Sidak method)	-	t = 0.181	0.025	0.856	No	48 Hr EtOH vs 24 Hr EtOH in Withdrawal
						All Pairwise Multiple Comparison Procedures (Holm-Sidak method)	-	t = 0.0355	0.050	0.972	No	24 Hr EtOH vs 72 Hr EtOH in Withdrawal
						All Pairwise Multiple Comparison Procedures (Holm-Sidak method)	-	t = 1.989	0.009	0.048	No	Air vs 72 Hr EtOH in Recovery
						All Pairwise Multiple Comparison Procedures (Holm-Sidak method)	-	t = 1.830	0.010	0.069	No	Air vs 24 Hr EtOH in Recovery
						All Pairwise Multiple Comparison Procedures (Holm-Sidak method)	-	t = 1.800	0.013	0.073	No	Air vs 48 Hr EtOH in Recovery
						All Pairwise Multiple Comparison Procedures (Holm-Sidak method)	-	t = 0.591	0.017	0.555	No	48 Hr EtOH vs 72 Hr EtOH in Recovery

						Test Type	DF	F- / T- / Z-Value	Critical Level	P-value	Significant?	Comments
						All Pairwise Multiple Comparison Procedures (Holm-Sidak method)	-	t = 0.399	0.025	0.691	No	48 Hr EtOH vs 24 Hr EtOH in Recovery
						All Pairwise Multiple Comparison Procedures (Holm-Sidak method)	-	t = 0.160	0.050	0.873	No	72 Hr EtOH vs 24 Hr EtOH in Recovery
	**Figure #**	**Figure Letter**	**Animal #**	**Cell #**	**normality**	**Test Type**	**DF**	**F- / T- / Z-Value**	**Critical Level**	**P-value**	**Significant?**	**Comments**
						24, 48, 72 Hour Air Control-Rotorod Perfomance						
Rotorod Performance	2	C	56		Assumed N≥30	2-Way RM ANOVA	2	F = 0.614	0.05	0.55	No	Air Duration- NO MAIN EFFECT
						2-Way RM ANOVA	3	F = 2.280	0.05	0.114	No	Trial-NO MAIN EFFECT
						**2-Way RM ANOVA**	**6**	**F = 0.515**	**0.05**	**0.725**	**No**	**Air Duration x Trial- NO INTERACTION**
						**Mixed Factor RM ANOVA**	**3,48**	**F = 1.254**	**0.050**	**0.301**	**No**	**EtOH Duration- NO MAIN EFFECT**
						**Mixed Factor RM ANOVA**	**2, 96**	**F = 37.344**	**0.050**	**< 0.001**	**Yes**	**Trial-MAIN EFFECT: Main effects cannot be properly interpreted if significant interaction is determined. See post hoc comparisons.**
						**Mixed Factor RM ANOVA**	**1, 48**	**F = 0.602**	**0.050**	**0.442**	**No**	**Sex-NO MAIN EFFECT**
						Mixed Factor RM ANOVA	2,96	F = 0.475	0.050	0.580	No	Trial x Sex- NO INTERACTION
						**Mixed Factor RM ANOVA**	**3,96**	**F = 0.861**	**0.050**	**< 0.001**	**Yes**	**Trial x EtOH Duration- INTERACTION: The effect of different levels of Trial depends on what level of EtOH Duration is present. There is a statistically significant interaction between EtOH Duration and Trial**
						Mixed Factor RM ANOVA	3,48	F = 0.705	0.050	0.553	No	EtOH Duration x Sex-NO INTERACTION
						Mixed Factor RM ANOVA	9,96	F = 0.861	0.050	0.507	No	Trial x Sex x Group- NO INTERACTION
						All Pairwise Multiple Comparison Procedures (Holm-Sidak method)	-	t = 1.931	0.017	0.058	No	Baseline vs Recovery in Air
						All Pairwise Multiple Comparison Procedures (Holm-Sidak method)	-	t = 1.228	0.025	0.224	No	Baseline vs Withdrawal in Air
						All Pairwise Multiple Comparison Procedures (Holm-Sidak method)	-	t = 0.703	0.050	0.484	No	Withdrawal vs Recovery in Air
						All Pairwise Multiple Comparison Procedures (Holm-Sidak method)	-	t = 4.879	0.017	< 0.001	Yes	Withdrawal vs Recovery in 72 Hr EtOH
						All Pairwise Multiple Comparison Procedures (Holm-Sidak method)	-	t = 2.558	0.025	0.013	Yes	Baseline vs Recovery in 72 Hr EtOH
						All Pairwise Multiple Comparison Procedures (Holm-Sidak method)	-	t = 2.321	0.050	0.023	Yes	Baseline vs Withdrawal in 72 Hr EtOH
						All Pairwise Multiple Comparison Procedures (Holm-Sidak method)	-	t = 5.201	0.017	< 0.001	Yes	Withdrawal vs Recovery in 24 Hr EtOH
						All Pairwise Multiple Comparison Procedures (Holm-Sidak method)	-	t = 3.847	0.025	< 0.001	Yes	Baseline vs Recovery in 24 Hr EtOH
						All Pairwise Multiple Comparison Procedures (Holm-Sidak method)	-	t = 1.354	0.050	0.180	No	Baseline vs Withdrawal in 24 Hr EtOH
						All Pairwise Multiple Comparison Procedures (Holm-Sidak method)	-	t = 3.397	0.017	0.001	Yes	Withdrawal vs Recovery in 48 Hr EtOH
						All Pairwise Multiple Comparison Procedures (Holm-Sidak method)	-	t = 2.752	0.025	0.008	Yes	Baseline vs Withdrawal in 48 Hr EtOH
						All Pairwise Multiple Comparison Procedures (Holm-Sidak method)	-	t = 0.644	0.050	0.522	No	Baseline vs Recovery in 48 Hr EtOH
						**All Pairwise Multiple Comparison Procedures (Holm-Sidak method)**	**-**	**t = 3.388**	**0.009**	**0.001**	**Yes**	**Air vs 48 Hr EtOH in Withdrawal**
						**All Pairwise Multiple Comparison Procedures**	**-**	**t = 2.962**	**0.010**	**0.004**	**Yes**	**Air vs 72 Hr EtOH in Withdrawal**
						All Pairwise Multiple Comparison Procedures (Holm-Sidak method)	-	t = 2.158	0.013	0.034	No	Air vs 24 Hr EtOH in Withdrawal
						All Pairwise Multiple Comparison Procedures (Holm-Sidak method)	-	t = 1.167	0.017	0.247	No	24 Hr EtOH vs 48 Hr EtOH in Withdrawal
						All Pairwise Multiple Comparison Procedures (Holm-Sidak method)	-	t = 0.635	0.025	0.527	No	24 Hr EtOH vs 72 Hr EtOH in Withdrawal

						All Pairwise Multiple Comparison Procedures (Holm-Sidak method)	-	t = 0.595	0.050	0.553	No	72 Hr EtOH vs 48 Hr EtOH in Withdrawal
						All Pairwise Multiple Comparison Procedures (Holm-Sidak method)	-	t = 2.673	0.009	0.009	No	24 Hr EtOH vs 48 Hr EtOH in Recovery
						All Pairwise Multiple Comparison Procedures (Holm-Sidak method)	-	t = 1.865	0.010	0.066	No	Air vs 24 Hr EtOH in Recovery
						All Pairwise Multiple Comparison Procedures (Holm-Sidak method)	-	t = 1.446	0.013	0.152	No	72 Hr EtOH vs 48 Hr EtOH in Recovery
						All Pairwise Multiple Comparison Procedures (Holm-Sidak method)	-	t = 1.372	0.017	0.174	No	24 Hr EtOH vs 72 Hr EtOH in Recovery
						All Pairwise Multiple Comparison Procedures (Holm-Sidak method)	-	t = 0.953	0.025	0.344	No	Air vs 48 Hr EtOH in Recovery
						All Pairwise Multiple Comparison Procedures (Holm-Sidak method)	-	t = 0.523	0.050	0.602	No	Air vs 72 Hr EtOH in Recovery

	Figure #	Figure Letter	Animal #	Cell #	normality	Test Type	DF	F- / T- / Z-Value	Critical Level	P-value	Significant?	Comments
USVs During Withdrawal	3	C-D	40		Assumed N≥30	**Mixed Factorial ANOVA**	**3,32**	**F = 2.740**	**0.050**	**0.059**	**No**	**EtOH Duration - NO MAIN EFFECT: The difference in the mean values among the different levels of EtOH Duration is greater than would be expected by chance after allowing for effects of differences in Frequency.**
						**Mixed Factorial ANOVA**	**5,160**	**F= 23.454**	**0.050**	**< 0.001**	**Yes**	**Frequency - MAIN EFFECT: The difference in the mean values among the different levels of Frequesncy is greater than would be expected by chance after allowing for effects of differences in EtOH Duration.**
						**Mixed Factorial ANOVA**	**1, 32**	**F= 0.036**	**0.050**	**0.852**	**No**	**Sex- NO MAIN EFFECT**
						Mixed Factorial ANOVA	**5,160**	**F = 0.248**	**0.050**	**0.795**	**No**	**Frequency x Sex - NO INTERACTION**
						Mixed Factorial ANOVA	**3,32**	**F = 1.834**	**0.050**	**0.161**	**No**	**Sex x EtOH Duration- NO INTERACTION**
						**Mixed Factorial ANOVA**	**15,160**	**F = 2.466**	**0.050**	**0.029**	**Yes**	**EtOH Duration vs Frequency - INTERACTION: The effect of different levels of EtOH Duration depends on what level of Frequency is present.**
						Mixed Factorial ANOVA	**15,160**	**F = 0.892**	**0.050**	**0.511**	**No**	**Sex x Frequency x EtOH Duration**
						**All Pairwise Multiple Comparison Procedures (Holm-Sidak method)**	**-**	**t = 5.335**	**0.003**	**< 0.001**	**Yes**	**0-20kHz vs 100-120kHz**
						**All Pairwise Multiple Comparison Procedures (Holm-Sidak method)**	**-**	**t = 4.924**	**0.004**	**< 0.001**	**Yes**	**0-20kHz vs 180-100kHz**
						**All Pairwise Multiple Comparison Procedures (Holm-Sidak method)**	**-**	**t = 4.686**	**0.004**	**< 0.001**	**Yes**	**0-20kHz vs 60-80kHz**
						**All Pairwise Multiple Comparison Procedures (Holm-Sidak method)**	**-**	**t = 3.578**	**0.004**	**< 0.001**	**Yes**	**20-40kHz vs 100-120kHz**
						**All Pairwise Multiple Comparison Procedures (Holm-Sidak method)**	**-**	**t = 3.182**	**0.005**	**0.002**	**Yes**	**0-20kHz vs 40-60kHz**
						**All Pairwise Multiple Comparison Procedures (Holm-Sidak method)**	**-**	**t = 3.166**	**0.005**	**0.002**	**Yes**	**20-40kHz vs 80-100kHz**
						**All Pairwise Multiple Comparison Procedures (Holm-Sidak method)**	**-**	**t = 2.929**	**0.006**	**0.004**	**Yes**	**20-40kHz vs 60-80kHz**
						All Pairwise Multiple Comparison Procedures (Holm-Sidak method)	-	t = 2.153	0.006	0.034	No	40-60kHz vs 100-120kHz
						All Pairwise Multiple Comparison Procedures (Holm-	-	t = 1.757	0.007	0.082	No	0-20kHz vs 20-40kHz
						All Pairwise Multiple Comparison Procedures (Holm-Sidak method)	-	t = 1.741	0.009	0.084	No	40-60kHz vs 80-100kHz
						All Pairwise Multiple Comparison Procedures (Holm-Sidak method)	-	t = 1.504	0.010	0.135	No	40-60kHz vs 60-80kHz

						All Pairwise Multiple Comparison Procedures (Holm-Sidak method)	-	t = 1.425	0.013	0.157	No	20-40kHz vs 40-60kHz
						All Pairwise Multiple Comparison Procedures (Holm-Sidak method)	-	t = 0.649	0.017	0.518	No	60-80kHz vs 100-120kHz
						All Pairwise Multiple Comparison Procedures (Holm-Sidak method)	-	t = 0.412	0.025	0.681	No	80-100kHz vs 100-120kHz
						All Pairwise Multiple Comparison Procedures (Holm-Sidak method)	-	t = 0.237	0.050	0.813	No	60-80kHz vs 80-100kHz
						**All Pairwise Multiple Comparison Procedures (Holm-Sidak method)**	**-**	**t = 3.254**	**0.050**	**0.002**	**Yes**	**72 Hour EtOH vs Air**
CORT	3	E	12		Yes	**Mixed Factorial ANOVA**	**1,8**	**F = 3.610**	**0.050**	**0.094**	**No**	**Sex - NO MAIN EFFECT**
						**Mixed Factorial ANOVA**	**1,8**	**F = 27.496**	**0.050**	**0.001**	**Yes**	**EtOH Treatment- MAIN EFFECT: Main effects cannot be properly interpreted if significant interaction is determined. See post hoc comparisons.**
						**Mixed Factorial ANOVA**	**2,16**	**F = 4.951**	**0.050**	**< 0.001**	**Yes**	**Trial - MAIN EFFECT: Main effects cannot be properly interpreted if significant interaction is determined. See post hoc comparisons.**
						**Mixed Factorial ANOVA**	**1,16**	**F = 20.249**	**0.050**	**0.001**	**Yes**	**EtOH Duration vs Trial - INTERACTION: The effect of different levels of EtOH Duration depends on what level of Trial is present. There is a statistically significant interaction between EtOH Duration and Trial.**
						**Mixed Factorial ANOVA**	**1,8**	**F = 1.970**	**0.050**	**0.198**	**No**	**Sex vs EtOH Duration - NO INTERACTION**
						**Mixed Factorial ANOVA**	**2,16**	**F = 3.373**	**0.050**	**0.060**	**No**	**Sex vs Trial - NO INTERACTION**
						**Mixed Factorial ANOVA**	**2,16**	**F = 0.376**	**0.050**	**0.004**	**Yes**	**Sex vs Trial vs EtOH Duration - NO INTERACTION**
						All Pairwise Multiple Comparison Procedures (Holm-Sidak method)	-	t = 5.997	0.017	< 0.001	Yes	Withdrawal + Stress vs Withdrawal in Air
						All Pairwise Multiple Comparison Procedures (Holm-Sidak method)	-	t = 5.114	0.025	< 0.001	Yes	Withdrawal + Stress vs Baseline in Air
						All Pairwise Multiple Comparison Procedures (Holm-	-	t = 0.884	0.050	0.387	No	Baseline vs Withdrawal in Air
						All Pairwise Multiple Comparison Procedures (Holm-Sidak method)	-	t = 9.898	0.017	< 0.001	Yes	Withdrawal + Stress vs Baseline in 72 Hr EtOH
						All Pairwise Multiple Comparison Procedures (Holm-Sidak method)	-	t = 6.239	0.025	< 0.001	Yes	Withdrawal + Stress vs Withdrawal in 72 Hr EtOH
						All Pairwise Multiple Comparison Procedures (Holm-Sidak method)	-	t = 3.659	0.050	0.002	Yes	Withdrawal vs Baseline in 72 Hr EtOH
						**All Pairwise Multiple Comparison Procedures (Holm-Sidak method)**	**-**	**t = 0.184**	**0.050**	**0.856**	**No**	**Air vs 72 Hr EtOH in Baseline**
						**All Pairwise Multiple Comparison Procedures (Holm-Sidak method)**	**-**	**t = 4.317**	**0.050**	**< 0.001**	**Yes**	**Air vs 72 Hr EtOH in Withdrawal**
						**All Pairwise Multiple Comparison Procedures (Holm-Sidak method)**	**-**	**t = 4.537**	**0.050**	**< 0.001**	**Yes**	**Air vs 72 Hr EtOH in Withdrawal + Stress**
CORT Stress Reactivity	3	F	12		Yes	**t-test**	**10**	**t= -0.165**	**0.05**	**0.872**	**No**	**The difference in the mean values of stress reactiviry between Air and 72 Hr EtOH is not great enough to reject the possibility that the difference is due to random sampling variability.**
	**Figure #**	**Figure Letter**	**Animal #**	**Cell #**	**normality**	**Test Type**	**DF**	**F- / T- / Z-Value**	**Critical Level**	**P-value**	**Significant?**	**Comments**
						**Mixed Factorial ANOVA**	**3,30**	**F = 1.465**	**0.050**	**0.244**	**No**	**EtOH Treatment - NO MAIN EFFECT**
						**Mixed Factorial ANOVA**	**3, 90**	**F = 40.523**	**0.05**	**< 0.001**	**Yes**	**Trial - MAIN EFFECT: Main effects cannot be properly interpreted if significant interaction is determined. See post hoc comparisons.**
						**Mixed Factorial ANOVA**	**1,30**	**F = 1.680**	**0.050**	**0.205**	**No**	**Sex - NO MAIN EFFECT**
						**Mixed Factorial ANOVA**	**3,30**	**F = 1.435**	**0.050**	**0.252**	**No**	**Sex x EtOH Treatment - NO INTERACTION**
						**Mixed Factorial ANOVA**	**3,90**	**F = 0.596**	**0.050**	**0.619**	**No**	**Trial x Sex - NO INTERACTION**

Rotorod Learning	4	A	38		Assumed N>30	**Mixed Factorial ANOVA**	**9,90**	**F = 8.491**	**0.050**	**< 0.001**	**Yes**	**EtOH Treatment x Trial - INTERACTION: The effect of different levels of Condition depends on what level of Trial is present. There is a statistically significant interaction between EtOH Treatment and Session**
						**Mixed Factorial ANOVA**	**9,90**	**F = 8.652**	**0.050**	**< 0.001**	**Yes**	**Trial x Sex x EtOH Treatment - NO INTERACTION**
						All Pairwise Multiple Comparison Procedures (Holm-Sidak method)	-	t = 0.922	0.009	0.359	No	Baseline vs Intoxication in 72 Hr Air
						All Pairwise Multiple Comparison Procedures (Holm-Sidak method)	-	t = 0.685	0.010	0.495	No	Baseline vs Withdrawal in 72 Hr Air
						All Pairwise Multiple Comparison Procedures (Holm-Sidak method)	-	t = 0.667	0.013	0.506	No	Baseline vs Recovery in 72 Hr Air
						All Pairwise Multiple Comparison Procedures (Holm-Sidak method)	-	t = 0.255	0.017	0.799	No	Intoxication vs Recovery in 72 Hr Air
						All Pairwise Multiple Comparison Procedures (Holm-Sidak method)	-	t = 0.237	0.025	0.813	No	Intoxication vs Withdrawal in 72 Hr Air
						All Pairwise Multiple Comparison Procedures (Holm-Sidak method)	-	t = 0.0178	0.050	0.986	No	Withdrawal vs Recovery in 72 Hr Air
						All Pairwise Multiple Comparison Procedures (Holm-Sidak method)	-	t = 5.213	0.009	< 0.001	Yes	Withdrawal vs Recovery in 72 Hr EtOH
						All Pairwise Multiple Comparison Procedures (Holm-Sidak method)	-	t = 3.958	0.010	< 0.001	Yes	Intoxication vs Recovery in 72 Hr EtOH
						All Pairwise Multiple Comparison Procedures (Holm-Sidak method)	-	t = 2.896	0.013	0.005	Yes	Baseline vs Withdrawal in 72 Hr EtOH
						All Pairwise Multiple Comparison Procedures (Holm-Sidak method)	-	t = 2.318	0.017	0.022	No	Baseline vs Recovery in 72 Hr EtOH
						All Pairwise Multiple Comparison Procedures (Holm-Sidak method)	-	t = 1.640	0.025	0.104	No	Baseline vs Intoxication in 72 Hr EtOH
						All Pairwise Multiple Comparison Procedures (Holm-Sidak method)	-	t = 1.255	0.050	0.212	No	Intoxication vs Withdrawal in 72 Hr EtOH
						All Pairwise Multiple Comparison Procedures (Holm-Sidak method)	-	t = 7.088	0.009	< 0.001	Yes	Baseline vs Withdrawal in 72 Hr EtOH + 30mM EtOH
						All Pairwise Multiple Comparison Procedures (Holm-Sidak method)	-	t = 6.638	0.010	< 0.001	Yes	Withdrawal vs Recovery in 72 Hr EtOH + 30mM EtOH
						All Pairwise Multiple Comparison Procedures (Holm-Sidak method)	-	t = 5.507	0.013	< 0.001	Yes	Intoxication vs Withdrawal in 72 Hr EtOH + 30mM EtOH
						All Pairwise Multiple Comparison Procedures (Holm-Sidak method)	-	t = 2.031	0.017	0.045	No	Baseline vs Intoxication in 72 Hr EtOH + 30mM EtOH
						All Pairwise Multiple Comparison Procedures (Holm-Sidak method)	-	t = 1.581	0.025	0.117	No	Intoxication vs Recovery in 72 Hr EtOH + 30mM EtOH
						All Pairwise Multiple Comparison Procedures (Holm-Sidak method)	-	t = 0.450	0.050	0.654	No	Baseline vs Recovery in 72 Hr EtOH + 30mM EtOH
						All Pairwise Multiple Comparison Procedures (Holm-Sidak method)	-	t = 8.470	0.009	< 0.001	Yes	Withdrawal vs Recovery in 72 Hour Air + 30mM EtOH
						All Pairwise Multiple Comparison Procedures (Holm-Sidak method)	-	t = 8.212	0.010	< 0.001	Yes	Intoxication vs Withdrawal in 72 Hour Air + 30mM EtOH
						All Pairwise Multiple Comparison Procedures (Holm-Sidak method)	-	t = 7.239	0.013	< 0.001	Yes	Baseline vs Withdrawal in 72 Hour Air + 30mM EtOH
						All Pairwise Multiple Comparison Procedures (Holm-Sidak method)	-	t = 1.231	0.017	0.221	No	Baseline vs Recovery in 72 Hour Air + 30mM EtOH
						All Pairwise Multiple Comparison Procedures (Holm-Sidak method)	-	t = 0.973	0.025	0.333	No	Baseline vs Intoxication in 72 Hour Air + 30mM EtOH
						All Pairwise Multiple Comparison Procedures (Holm-Sidak method)	-	t = 0.258	0.050	0.797	No	Intoxication vs Recovery in 72 Hour Air + 30mM EtOH
						All Pairwise Multiple Comparison Procedures (Holm-Sidak method)	-	t = 1.945	0.009	0.056	No	72 Hour EtOH + 30mM EtOH vs 72 Hour EtOH in Baseline
						All Pairwise Multiple Comparison Procedures (Holm-Sidak method)	-	t = 1.732	0.010	0.088	No	72 Hour EtOH + 30mM EtOH vs 72 HourAir + 30mM EtOH in Baseline

All Pairwise Multiple Comparison Procedures (Holm-Sidak method)	-	t = 1.584	0.013	0.118	No	72 Hour EtOH + 30mM EtOH vs 72 Hour Air in Baseline
All Pairwise Multiple Comparison Procedures (Holm-Sidak method)	-	t = 0.360	0.017	0.720	No	72 Hour EtOH vs 72 Hour Air in Baseline
All Pairwise Multiple Comparison Procedures (Holm-Sidak method)	-	t = 0.238	0.025	0.812	No	72 Hour Air + 30mM EtOH vs 72 Hour Air in Baseline
All Pairwise Multiple Comparison Procedures (Holm-Sidak method)	-	t = 0.101	0.050	0.920	No	72 Hour Air + 30mM EtOH vs 72 Hour EtOH in Baseline
All Pairwise Multiple Comparison Procedures (Holm-Sidak method)	-	t = 2.088	0.009	0.040	No	72 Hour Air vs 72 Hour EtOH in Intoxication
All Pairwise Multiple Comparison Procedures (Holm-Sidak method)	-	t = 1.836	0.010	0.071	No	72 Hour Air + 30mM EtOH vs 72 Hour EtOH in Intoxication
All Pairwise Multiple Comparison Procedures (Holm-Sidak method)	-	t = 1.681	0.013	0.097	No	72 Hour EtOH + 30mM EtOH vs 72 Hour EtOH in Intoxication
All Pairwise Multiple Comparison Procedures (Holm-Sidak method)	-	t = 0.407	0.017	0.685	No	72 Hour Air vs 72 Hour EtOH + 30mM EtOH in Intoxication
All Pairwise Multiple Comparison Procedures (Holm-Sidak method)	-	t = 0.251	0.025	0.803	No	72 Hour Air + 30mM EtOHvs 72 Hour EtOH + 30mM EtOH in Intoxication
All Pairwise Multiple Comparison Procedures (Holm-Sidak method)	-	t = 0.133	0.050	0.894	No	72 Hour Air vs 72 Hour Air + 30mM EtOH in Intoxication
All Pairwise Multiple Comparison Procedures (Holm-Sidak method)	**-**	**t = 5.820**	**0.009**	**< 0.001**	**Yes**	**72 Hour Air + 30mM EtOH vs 72 Hour Air in Withdrawal**
All Pairwise Multiple Comparison Procedures (Holm-Sidak method)	**-**	**t = 3.658**	**0.010**	**< 0.001**	**Yes**	**72 Hour EtOH + 30mM EtOH vs 72 Hour Air in Withdrawal**
All Pairwise Multiple Comparison Procedures (Holm-Sidak method)	**-**	**t = 3.204**	**0.013**	**0.002**	**Yes**	**72 Hour Air + 30mM EtOH vs 72 Hour EtOH in Withdrawal**
All Pairwise Multiple Comparison Procedures (Holm-Sidak method)	**-**	**t = 2.775**	**0.017**	**0.007**	**Yes**	**72 Hour EtOH vs 72 Hour Air in Withdrawal**
All Pairwise Multiple Comparison Procedures (Holm-Sidak method)	**-**	**t = 2.371**	**0.025**	**0.020**	**Yes**	**72 Hour Air + 30mM EtOH vs 72 Hour EtOH + 30mM EtOH in Withdrawal**
All Pairwise Multiple Comparison Procedures (Holm-Sidak method)	**-**	**t = 0.883**	**0.050**	**0.380**	**No**	**72 Hour EtOH + 30mM EtOH vs 72 Hour EtOH in Withdrawal**
All Pairwise Multiple Comparison Procedures (Holm-Sidak method)	-	t = 0.831	0.009	0.409	No	72 Hour Air vs 72 Hour EtOH + 30mM EtOH in Recovery
All Pairwise Multiple Comparison Procedures (Holm-Sidak method)	-	t = 0.753	0.010	0.454	No	72 Hour Air vs 72 Hour EtOH in Recovery
All Pairwise Multiple Comparison Procedures (Holm-Sidak method)	-	t = 0.571	0.013	0.570	No	72 Hour Air + 30mM EtOH vs 72 Hour EtOH + 30mM EtOH in Recovery
All Pairwise Multiple Comparison Procedures (Holm-Sidak method)	-	t = 0.497	0.017	0.620	No	72 Hour Air + 30mM EtOH vs 72 Hour EtOH in Recovery
All Pairwise Multiple Comparison Procedures (Holm-Sidak method)	-	t = 0.212	0.025	0.832	No	72 Hour Air + 30mM EtOH vs 72 Hour Air in Recovery
All Pairwise Multiple Comparison Procedures (Holm-Sidak method)	-	t = 0.0780	0.050	0.938	No	72 Hour EtOH vs 72 Hour EtOH + 30mM EtOH in Recovery

**Mixed Factorial ANOVA**	**2,60**	**F = 25.117**	**0.050**	**< 0.001**	**Yes**	**Trial - MAIN EFFECT:The difference in the mean values among the different levels of EtOH Treatment is greater than would be expected by chance after allowing for effects of differences in Frequency.**
**Mixed Factorial ANOVA**	**1,30**	**F = 0.307**	**0.050**	**0.584**	**No**	**Sex - NO MAIN EFFECT**
**Mixed Factorial ANOVA**	**3,30**	**F = 0.2039**	**0.05**	**0.13**	**No**	**EtOH Treatment - NO MAIN EFFECT**
Mixed Factorial ANOVA	3,30	F = 0.932	0.050	0.438	No	Sex x EtOH Treatment - NO INTERACTION
Mixed Factorial ANOVA	2,60	F = 0.790	0.050	0.459	No	Trial x Sex - NO INTERACTION
**Mixed Factorial ANOVA**	**6,60**	**F = 3.802**	**0.050**	**0.003**	**Yes**	**EtOH Treatment x Trial - INTERACTION: The effect of different levels of EtOH Treatment depends on what level of Trial is present.**

						**Mixed Factorial ANOVA**	6,60	F = 2.373	0.050	0.040	Yes	Trial x Sex x EtOH Treatment - INTERACTION: The effect of different levels of EtOH Treatment depends on what level of Trial and Sex is present.
						All Pairwise Multiple Comparison Procedures (Holm-Sidak method)	-	t = 4.995	0.017	< 0.001	Yes	Withdrawal vs Recovery in 72 Hr EtOH
						All Pairwise Multiple Comparison Procedures (Holm-Sidak method)	-	t = 2.553	0.025	0.013	Yes	Baseline vs Recovery in 72 Hr EtOH
						All Pairwise Multiple Comparison Procedures (Holm-Sidak method)	-	t = 2.443	0.050	0.017	Yes	Baseline vs Withdrawal in 72 Hr EtOH
						All Pairwise Multiple Comparison Procedures (Holm-Sidak method)	-	t = 1.376	0.017	0.173	No	Baseline vs Recovery in Air
						All Pairwise Multiple Comparison Procedures (Holm-Sidak method)	-	t = 0.751	0.025	0.455	No	Withdrawal vs Recovery in Air
						All Pairwise Multiple Comparison Procedures (Holm-Sidak method)	-	t = 0.624	0.050	0.534	No	Baseline vs Withdrawal in Air
						All Pairwise Multiple Comparison Procedures (Holm-Sidak method)	-	t = 2.778	0.017	0.007	Yes	Withdrawal vs Recovery in 72 Hr EtOH Withdrawal + 30mM EtOH
						All Pairwise Multiple Comparison Procedures (Holm-Sidak method)	-	t = 1.431	0.025	0.157	No	Baseline vs Recovery in 72 Hr EtOH Withdrawal + 30mM EtOH
						All Pairwise Multiple Comparison Procedures (Holm-Sidak method)	-	t = 1.347	0.050	0.182	No	Baseline vs Withdrawal in 72 Hr EtOH Withdrawal + 30mM EtOH
						All Pairwise Multiple Comparison Procedures (Holm-Sidak method)	-	t = 4.707	0.017	< 0.001	Yes	Withdrawal vs Recovery in 72 Hr Air Withdrawal + 30mM EtOH
						All Pairwise Multiple Comparison Procedures (Holm-Sidak method)	-	t = 4.622	0.025	< 0.001	Yes	Baseline vs Withdrawal in 72 Hr Air Withdrawal + 30mM EtOH
Rotorod Performance	4	B		38		Assumed N≥30	All Pairwise Multiple Comparison Procedures (Holm-Sidak method)	-	t = 0.085	0.050	0.932	No	Baseline vs Recovery in 72 Hr Air Withdrawal + 30mM EtOH
						All Pairwise Multiple Comparison Procedures (Holm-Sidak method)	-	t = 0.717	0.009	0.476	No	72 Hr Air Withdrawal + 30mM EtOH and 72 Hour EtOH Withdrawal + 30mM EtOH in Baseline
						All Pairwise Multiple Comparison Procedures (Holm-Sidak method)	-	t = 0.644	0.010	0.522	No	72 Hr EtOH and 72 Hour EtOH Withdrawal + 30mM EtOH in Baseline
						All Pairwise Multiple Comparison Procedures (Holm-Sidak method)	-	t = 0.615	0.013	0.541	No	72 Hr Air Withdrawal + 30mM EtOH and 72 Hr Air in Baseline
						All Pairwise Multiple Comparison Procedures (Holm-Sidak method)	-	t = 0.535	0.017	0.594	No	72 Hr Air and 72 Hour EtOH in Baseline
						All Pairwise Multiple Comparison Procedures (Holm-Sidak method)	-	t = 0.110	0.025	0.913	No	72 Hr Air Withdrawal + 30mM EtOH and 72 Hour EtOH in Baseline
						All Pairwise Multiple Comparison Procedures (Holm-Sidak method)	-	t = 0.108	0.050	0.914	No	72 Hr Air and 72 Hour EtOH Withdrawal + 30mM EtOH in Baseline
						All Pairwise Multiple Comparison Procedures (Holm-Sidak method)	-	**t = 4.028**	**0.009**	**< 0.001**	**Yes**	**72 Hr Air and 72 Air Withdrawal + 30mM EtOH in Withdrawal**
						All Pairwise Multiple Comparison Procedures (Holm-Sidak method)	-	**t = 2.969**	**0.010**	**0.004**	**Yes**	**72 Hr EtOH Withdrawal + 30mM EtOH and 72 Air Withdrawal + 30mM EtOH in Withdrawal**
						All Pairwise Multiple Comparison Procedures (Holm-Sidak method)	-	t = 2.452	0.013	0.017	No	72 Hr Air and 72 EtOH in Withdrawal
						All Pairwise Multiple Comparison Procedures (Holm-Sidak method)	-	t = 1.716	0.017	0.091	No	72 Hr EtOH and 72 EtOH Withdrawal + 30mM EtOH in Withdrawal
						All Pairwise Multiple Comparison Procedures (Holm-Sidak method)	-	t = 1.328	0.025	0.189	No	72 Hr EtOH and 72 Air Withdrawal + 30mM EtOH in Withdrawal
						All Pairwise Multiple Comparison Procedures (Holm-Sidak method)	-	**t = 1.124**	**0.050**	**0.266**	**No**	**72 Hr Air and 72 EtOH Withdrawal + 30mM EtOH in Withdrawal**
						All Pairwise Multiple Comparison Procedures (Holm-Sidak method)	-	t = 1.559	0.009	0.124	No	72 Hr Air Withdrawal + 30mM EtOH and 72 Hour EtOH in Recovery
						All Pairwise Multiple Comparison Procedures (Holm-Sidak method)	-	t = 1.505	0.010	0.138	No	72 Hr Air Withdrawal + 30mM EtOH and 72 Hour EtOH Withdrawal + 30mM EtOH in Recovery
						All Pairwise Multiple Comparison Procedures (Holm-Sidak method)	-	t = 1.370	0.013	0.176	No	72 Hr Air Withdrawal + 30mM EtOH and 72 Hr Air in Recovery

						All Pairwise Multiple Comparison Procedures (Holm-Sidak method)	-	t = 0.200	0.017	0.842	No	72 Hr Air and 72 Hour EtOH in Recovery
						All Pairwise Multiple Comparison Procedures (Holm-Sidak method)	-	t = 0.143	0.025	0.887	No	72 Hr EtOH Withdrawal + 30mM EtOH and 72 Hour Air in Recovery
						All Pairwise Multiple Comparison Procedures (Holm-Sidak method)	-	t = 0.0573	0.050	0.955	No	72 Hr ETOH and 72 Hour EtOH Withdrawal + 30mM EtOH in Recovery
USVs During Withdrawal	4	C-D	30		Assumed N≥30	**Mixed Factorial ANOVA**	**2,24**	**F = 4.928**	**0.050**	**0.016**	**Yes**	**EtOH Treatment - MAIN EFFECT:The difference in the mean values among the different levels of EtOH Treatment is greater than would be expected by chance after allowing for effects of differences in Frequency and Sex.**
						**Mixed Factorial ANOVA**	**1,24**	**F = 1.022**	**0.050**	**0.322**	**No**	**Sex - NO MAIN EFFECT**
						**Mixed Factorial ANOVA**	**5,120**	**F = 25.942**	**0.050**	**< 0.001**	**Yes**	**Frequency - MAIN EFFECT: The difference in the mean values among the different levels of Frequency is greater than would be expected by chance after allowing for effects of differences in EtOH Treatment and Sex.**
						Mixed Factorial ANOVA	2,24	F = 2.430	0.050	0.109	No	Sex x EtOH Treatment - NO INTERACTION
						Mixed Factorial ANOVA	5,120	F = 0.115	0.050	0.115	No	Frequency x Sex - NO INTERACTION
						**Mixed Factorial ANOVA**	**10,120**	**F = 3.691**	**0.050**	**0.007**	**Yes**	**Frequency x EtOH Treatment - INTERACTION: The effect on different levels of frequency are dependent on EtOH Treatments**
						Mixed Factorial ANOVA	10,120	F = 0.919	0.050	0.469	No	EtOH Treatment x Frequency x Sex- NO INTERACTION
						All Pairwise Multiple Comparison Procedures (Holm-Sidak method)	-	t = 6.150	0.003	< 0.001	Yes	0-20kHz vs 100-120kHz
						All Pairwise Multiple Comparison Procedures (Holm-Sidak method)	-	t = 5.649	0.004	< 0.001	Yes	0-20kHz vs 80-100kHz
						All Pairwise Multiple Comparison Procedures (Holm-Sidak method)	-	t = 5.362	0.004	< 0.001	Yes	0-20kHz vs 60-80kHz
						All Pairwise Multiple Comparison Procedures (Holm-Sidak method)	-	t = 5.061	0.004	< 0.001	Yes	20-40kHz vs 100-120kHz
						All Pairwise Multiple Comparison Procedures (Holm-Sidak method)	-	t = 4.559	0.005	< 0.001	Yes	20-40kHz vs 80-1000kHz
						All Pairwise Multiple Comparison Procedures (Holm-Sidak method)	-	t = 4.272	0.005	< 0.001	Yes	20-40kHz vs 60-80kHz
						All Pairwise Multiple Comparison Procedures (Holm-Sidak method)	-	t = 3.570	0.006	< 0.001	Yes	0-20kHz vs 40-60kHz
						All Pairwise Multiple Comparison Procedures (Holm-Sidak method)	-	t = 2.581	0.006	0.011	No	40-60kHz vs 100-120kHz
						All Pairwise Multiple Comparison Procedures (Holm-Sidak method)	-	t = 2.480	0.007	0.014	No	20-40kHz vs 40-60kHz
						All Pairwise Multiple Comparison Procedures (Holm-Sidak method)	-	t = 2.079	0.009	0.039	No	40-60kHz vs 80-100kHz
						All Pairwise Multiple Comparison Procedures (Holm-Sidak method)	-	t = 1.792	0.010	0.075	No	40-60kHz vs 60-80kHz
						All Pairwise Multiple Comparison Procedures (Holm-Sidak method)	-	t = 1.090	0.013	0.278	No	0-20kHz vs 20-40kHz
						All Pairwise Multiple Comparison Procedures (Holm-Sidak method)	-	t = 0.789	0.017	0.432	No	60-80kHz vs 100-120kHz
						All Pairwise Multiple Comparison Procedures (Holm-Sidak method)	-	t = 0.502	0.025	0.617	No	80-100kHz vs 100-120kHz
						All Pairwise Multiple Comparison Procedures (Holm-Sidak method)	-	t = 0.287	0.050	0.775	No	60-80kHz vs 80-100kHz
						All Pairwise Multiple Comparison Procedures (Holm-Sidak method)	-	t = 3.609	0.017	< 0.001	Yes	72 Hr EtOH vs Air

						**All Pairwise Multiple Comparison Procedures (Holm-Sidak method)**	**-**	**t = 3.061**	**0.025**	**0.003**	**Yes**	**72 Hr EtOH vs 72 Hr EtOH Withdrawal + 30mM EtOH**
						**All Pairwise Multiple Comparison Procedures (Holm-Sidak method)**	**-**	**t = 0.547**	**0.050**	**0.585**	**No**	**72 Hr EtOH Withdrawal + 30mM EtOH vs Air**
	**Figure #**	**Figure Letter**	**Animal #**	**Cell #**	**normality**	**Test Type**	**DF**	**F- / T- / Z-Value**	**Critical Level**	**P-value**	**Significant?**	**Comments**
CNO Change in Current	5	A	5	15	Yes	**Paired t-test**	**14**	**t = 2.066**	**0.050**	**0.058**	**No**	**There is no significant change in current during CNO application compared to baseline**
						Two Way RM ANOVA	13, 260	F = 3.116	0.050	0.061	No	Drug-NO MAIN EFFECT
						**Two Way RM ANOVA**	**10,260**	**F = 17.448**	**0.050**	**< 0.001**	**Yes**	**Stim Number - MAIN EFFECT:Main effects cannot be properly interpreted if significant interaction is determined. This is because the size of a factor's effect depends upon the level of the other factor.**
						Two Way RM ANOVA	20, 260	F = 2.502	0.050	< 0.001	Yes	Drug x Stim Number - INTERACTION: The effect of different levels of drug depends on what level of stim # is present.
						All Pairwise Multiple Comparison Procedures (Bonferroni)		t = 3.514	0.050	0.001	Yes	CNO vs Wash
						All Pairwise Multiple Comparison Procedures (Bonferroni)		t = 1.008	0.050	0.942	No	Baseline vs Wash
						**All Pairwise Multiple Comparison Procedures (Bonferroni)**		**t = 2.506**	**0.050**	**0.038**	**Yes**	**CNO vs Baseline**
						All Pairwise Multiple Comparison Procedures (Bonferroni)		t = 6.952	0.001	<0.001	Yes	10.000 vs. 1.000 in Baseline
						All Pairwise Multiple Comparison Procedures (Bonferroni)		t = 6.909	0.001	<0.001	Yes	10.000 vs. 0.000 in Baseline
						All Pairwise Multiple Comparison Procedures (Bonferroni)		t = 6.865	0.001	<0.001	Yes	9.000 vs. 1.000 in Baseline
						All Pairwise Multiple Comparison Procedures (Bonferroni)		t = 6.851	0.001	<0.001	Yes	7.000 vs. 1.000 in Baseline
						All Pairwise Multiple Comparison Procedures (Bonferroni)		t = 6.822	0.001	<0.001	Yes	8.000 vs. 1.000 in Baseline
						All Pairwise Multiple Comparison Procedures (Bonferroni)		t = 6.822	0.001	<0.001	Yes	9.000 vs. 0.000 in Baseline
						All Pairwise Multiple Comparison Procedures (Bonferroni)		t = 6.807	0.001	<0.001	Yes	7.000 vs. 0.000 in Baseline
						All Pairwise Multiple Comparison Procedures (Bonferroni)		t = 6.778	0.001	<0.001	Yes	8.000 vs. 0.000 in Baseline
						All Pairwise Multiple Comparison Procedures (Bonferroni)		t = 6.357	0.001	<0.001	Yes	6.000 vs. 1.000 in Baseline
						All Pairwise Multiple Comparison Procedures (Bonferroni)		t = 6.314	0.001	<0.001	Yes	6.000 vs. 0.000 in Baseline
						All Pairwise Multiple Comparison Procedures (Bonferroni)		t = 5.298	0.001	<0.001	Yes	10.000 vs. 2.000 in Baseline
						All Pairwise Multiple Comparison Procedures (Bonferroni)		t = 5.211	0.001	<0.001	Yes	9.000 vs. 2.000 in Baseline
						All Pairwise Multiple Comparison Procedures (Bonferroni)		t = 5.196	0.001	<0.001	Yes	7.000 vs. 2.000 in Baseline
						All Pairwise Multiple Comparison Procedures		t = 5.167	0.001	<0.001	Yes	8.000 vs. 2.000 in Baseline
						All Pairwise Multiple Comparison Procedures (Bonferroni)		t = 4.703	0.001	<0.001	Yes	6.000 vs. 2.000 in Baseline
						All Pairwise Multiple Comparison Procedures (Bonferroni)		t = 4.282	0.001	<0.001	Yes	10.000 vs. 3.000 in Baseline
						All Pairwise Multiple Comparison Procedures (Bonferroni)		t = 4.267	0.001	<0.001	Yes	5.000 vs. 1.000 in Baseline
						All Pairwise Multiple Comparison Procedures (Bonferroni)		t = 4.224	0.001	<0.001	Yes	5.000 vs. 0.000 in Baseline

All Pairwise Multiple Comparison Procedures (Bonferroni)		t = 4.195	0.001	<0.001	Yes	9.000 vs. 3.000 in Baseline
All Pairwise Multiple Comparison Procedures (Bonferroni)		t = 4.18	0.001	<0.001	Yes	7.000 vs. 3.000 in Baseline
All Pairwise Multiple Comparison Procedures (Bonferroni)		t = 4.151	0.001	<0.001	Yes	8.000 vs. 3.000 in Baseline
All Pairwise Multiple Comparison Procedures (Bonferroni)		t = 3.875	0.002	<0.001	Yes	10.000 vs. 4.000 in Baseline
All Pairwise Multiple Comparison Procedures (Bonferroni)		t = 3.788	0.002	<0.001	Yes	9.000 vs. 4.000 in Baseline
All Pairwise Multiple Comparison Procedures (Bonferroni)		t = 3.774	0.002	<0.001	Yes	7.000 vs. 4.000 in Baseline
All Pairwise Multiple Comparison Procedures (Bonferroni)		t = 3.745	0.002	<0.001	Yes	8.000 vs. 4.000 in Baseline
All Pairwise Multiple Comparison Procedures (Bonferroni)		t = 3.687	0.002	<0.001	Yes	6.000 vs. 3.000 in Baseline
All Pairwise Multiple Comparison Procedures (Bonferroni)		t = 3.28	0.002	0.001	Yes	6.000 vs. 4.000 in Baseline
All Pairwise Multiple Comparison Procedures (Bonferroni)		t = 3.077	0.002	0.002	No	4.000 vs. 1.000 in Baseline
All Pairwise Multiple Comparison Procedures (Bonferroni)		t = 3.034	0.002	0.003	No	4.000 vs. 0.000 in Baseline
All Pairwise Multiple Comparison Procedures (Bonferroni)		t = 2.685	0.002	0.008	No	10.000 vs. 5.000 in Baseline
All Pairwise Multiple Comparison Procedures (Bonferroni)		t = 2.671	0.002	0.008	No	3.000 vs. 1.000 in Baseline
All Pairwise Multiple Comparison Procedures (Bonferroni)		t = 2.627	0.002	0.009	No	3.000 vs. 0.000 in Baseline
All Pairwise Multiple Comparison Procedures (Bonferroni)		t = 2.613	0.002	0.009	No	5.000 vs. 2.000 in Baseline
All Pairwise Multiple Comparison Procedures (Bonferroni)		t = 2.598	0.002	0.01	No	9.000 vs. 5.000 in Baseline
All Pairwise Multiple Comparison Procedures (Bonferroni)		t = 2.584	0.002	0.01	No	7.000 vs. 5.000 in Baseline
All Pairwise Multiple Comparison Procedures (Bonferroni)		t = 2.555	0.003	0.011	No	8.000 vs. 5.000 in Baseline
All Pairwise Multiple Comparison Procedures (Bonferroni)		t = 2.09	0.003	0.037	No	6.000 vs. 5.000 in Baseline
All Pairwise Multiple Comparison Procedures (Bonferroni)		t = 1.655	0.003	0.099	No	2.000 vs. 1.000 in Baseline
All Pairwise Multiple Comparison Procedures (Bonferroni)		t = 1.611	0.003	0.108	No	2.000 vs. 0.000 in Baseline
All Pairwise Multiple Comparison Procedures (Bonferroni)		t = 1.597	0.003	0.111	No	5.000 vs. 3.000 in Baseline
All Pairwise Multiple Comparison Procedures (Bonferroni)		t = 1.422	0.003	0.156	No	4.000 vs. 2.000 in Baseline
All Pairwise Multiple Comparison Procedures (Bonferroni)		t = 1.19	0.004	0.235	No	5.000 vs. 4.000 in Baseline
All Pairwise Multiple Comparison Procedures (Bonferroni)		t = 1.016	0.004	0.31	No	3.000 vs. 2.000 in Baseline
All Pairwise Multiple Comparison Procedures (Bonferroni)		t = 0.595	0.004	0.552	No	10.000 vs. 6.000 in Baseline
All Pairwise Multiple Comparison Procedures (Bonferroni)		= 0.508	0.005	0.612	No	9.000 vs. 6.000 in Baseline
All Pairwise Multiple Comparison Procedures (Bonferroni)		t = 0.493	0.005	0.622	No	7.000 vs. 6.000 in Baseline
All Pairwise Multiple Comparison Procedures (Bonferroni)		t = 0.464	0.006	0.643	No	8.000 vs. 6.000 in Baseline

All Pairwise Multiple Comparison Procedures (Bonferroni)		t = 0.406	0.006	0.685	No	4.000 vs. 3.000 in Baseline
All Pairwise Multiple Comparison Procedures (Bonferroni)		t = 0.131	0.007	0.896	No	10.000 vs. 8.000 in Baseline
All Pairwise Multiple Comparison Procedures (Bonferroni)		t = 0.102	0.009	0.919	No	10.000 vs. 7.000 in Baseline
All Pairwise Multiple Comparison Procedures (Bonferroni)		t = 0.0871	0.01	0.931	No	10.000 vs. 9.000 in Baseline
All Pairwise Multiple Comparison Procedures (Bonferroni)		t = 0.0435	0.013	0.965	No	9.000 vs. 8.000 in Baseline
All Pairwise Multiple Comparison Procedures (Bonferroni)		t = 0.0435	0.017	0.965	No	0.000 vs. 1.000 in Baseline
All Pairwise Multiple Comparison Procedures (Bonferroni)		t = 0.029	0.025	0.977	No	7.000 vs. 8.000 in Baseline
All Pairwise Multiple Comparison Procedures (Bonferroni)		t = 0.0145	0.05	0.988	No	9.000 vs. 7.000 in Baseline
All Pairwise Multiple Comparison Procedures (Bonferroni)		t=3.643	0.001	<0.001	Yes	10.000 vs. 1.000 in CNO
All Pairwise Multiple Comparison Procedures (Bonferroni)		t=3.6	0.001	<0.001	Yes	6.000 vs. 1.000 in CNO
All Pairwise Multiple Comparison Procedures (Bonferroni)		t=3.382	0.001	<0.001	Yes	10.000 vs. 0.000 in CNO
All Pairwise Multiple Comparison Procedures (Bonferroni)		t=3.338	0.001	<0.001	Yes	8.000 vs. 1.000 in CNO
All Pairwise Multiple Comparison Procedures (Bonferroni)		t=3.338	0.001	<0.001	Yes	6.000 vs. 0.000 in CNO
All Pairwise Multiple Comparison Procedures (Bonferroni)		t=3.208	0.001	0.001	No	9.000 vs. 1.000 in CNO
All Pairwise Multiple Comparison Procedures (Bonferroni)		t=3.092	0.001	0.002	No	7.000 vs. 1.000 in CNO
All Pairwise Multiple Comparison Procedures (Bonferroni)		t=3.077	0.001	0.002	No	8.000 vs. 0.000 in CNO
All Pairwise Multiple Comparison Procedures (Bonferroni)		t=2.946	0.001	0.003	No	9.000 vs. 0.000 in CNO
All Pairwise Multiple Comparison Procedures (Bonferroni)		t=2.83	0.001	0.005	No	7.000 vs. 0.000 in CNO
All Pairwise Multiple Comparison Procedures (Bonferroni)		t=2.729	0.001	0.007	No	5.000 vs. 1.000 in CNO
All Pairwise Multiple Comparison Procedures (Bonferroni)		t=2.526	0.001	0.012	No	10.000 vs. 2.000 in CNO
All Pairwise Multiple Comparison Procedures (Bonferroni)		t=2.482	0.001	0.014	No	6.000 vs. 2.000 in CNO
All Pairwise Multiple Comparison Procedures (Bonferroni)		t=2.467	0.001	0.014	No	5.000 vs. 0.000 in CNO
All Pairwise Multiple Comparison Procedures (Bonferroni)		t=2.221	0.001	0.027	No	8.000 vs. 2.000 in CNO
All Pairwise Multiple Comparison Procedures (Bonferroni)		t=2.09	0.001	0.037	No	9.000 vs. 2.000 in CNO
All Pairwise Multiple Comparison Procedures (Bonferroni)		t=1.988	0.001	0.048	No	10.000 vs. 3.000 in CNO
All Pairwise Multiple Comparison Procedures (Bonferroni)		t=1.974	0.001	0.049	No	7.000 vs. 2.000 in CNO
All Pairwise Multiple Comparison Procedures (Bonferroni)		t=1.945	0.001	0.053	No	6.000 vs. 3.000 in CNO
All Pairwise Multiple Comparison Procedures (Bonferroni)		t=1.916	0.001	0.056	No	4.000 vs. 1.000 in CNO
All Pairwise Multiple Comparison Procedures (Bonferroni)		t=1.727	0.001	0.085	No	10.000 vs. 4.000 in CNO

PC Attached CNO APs	5	B	6	14	Yes	All Pairwise Multiple Comparison Procedures (Bonferroni)		t=1.684	0.002	0.093	No	8.000 vs. 3.000 in CNO
						All Pairwise Multiple Comparison Procedures (Bonferroni)		t=1.684	0.002	0.093	No	6.000 vs. 4.000 in CNO
						All Pairwise Multiple Comparison Procedures (Bonferroni)		t=1.655	0.002	0.099	No	3.000 vs. 1.000 in CNO
						All Pairwise Multiple Comparison Procedures (Bonferroni)		t=1.655	0.002	0.099	No	4.000 vs. 0.000 in CNO
						All Pairwise Multiple Comparison Procedures (Bonferroni)		t=1.611	0.002	0.108	No	5.000 vs. 2.000 in CNO
						All Pairwise Multiple Comparison Procedures (Bonferroni)		t=1.553	0.002	0.121	No	9.000 vs. 3.000 in CNO
						All Pairwise Multiple Comparison Procedures (Bonferroni)		t=1.437	0.002	0.152	No	7.000 vs. 3.000 in CNO
						All Pairwise Multiple Comparison Procedures (Bonferroni)		t=1.422	0.002	0.156	No	8.000 vs. 4.000 in CNO
						All Pairwise Multiple Comparison Procedures (Bonferroni)		t=1.393	0.002	0.165	No	3.000 vs. 0.000 in CNO
						All Pairwise Multiple Comparison Procedures (Bonferroni)		t=1.292	0.002	0.197	No	9.000 vs. 4.000 in CNO
						All Pairwise Multiple Comparison Procedures (Bonferroni)		t=1.176	0.002	0.241	No	7.000 vs. 4.000 in CNO
						All Pairwise Multiple Comparison Procedures (Bonferroni)		t=1.118	0.002	0.265	No	2.000 vs. 1.000 in CNO
						All Pairwise Multiple Comparison Procedures (Bonferroni)		t=1.074	0.002	0.284	No	5.000 vs. 3.000 in CNO
						All Pairwise Multiple Comparison Procedures (Bonferroni)		t=0.914	0.002	0.361	No	10.000 vs. 5.000 in CNO
						All Pairwise Multiple Comparison Procedures (Bonferroni)		t=0.871	0.003	0.385	No	6.000 vs. 5.000 in CNO
						All Pairwise Multiple Comparison Procedures (Bonferroni)		t=0.856	0.003	0.392	No	2.000 vs. 0.000 in CNO
						All Pairwise Multiple Comparison Procedures (Bonferroni)		t=0.813	0.003	0.417	No	5.000 vs. 4.000 in CNO
						All Pairwise Multiple Comparison Procedures (Bonferroni)		t=0.798	0.003	0.425	No	4.000 vs. 2.000 in CNO
						All Pairwise Multiple Comparison Procedures (Bonferroni)		t=0.61	0.003	0.543	No	8.000 vs. 5.000 in CNO
						All Pairwise Multiple Comparison Procedures (Bonferroni)		t=0.552	0.003	0.582	No	10.000 vs. 7.000 in CNO
						All Pairwise Multiple Comparison Procedures (Bonferroni)		t=0.537	0.004	0.592	No	3.000 vs. 2.000 in CNO
						All Pairwise Multiple Comparison Procedures (Bonferroni)		t=0.508	0.004	0.612	No	6.000 vs. 7.000 in CNO
						All Pairwise Multiple Comparison Procedures (Bonferroni)		t=0.479	0.004	0.632	No	9.000 vs. 5.000 in CNO
						All Pairwise Multiple Comparison Procedures (Bonferroni)		t=0.435	0.005	0.664	No	10.000 vs. 9.000 in CNO
						All Pairwise Multiple Comparison Procedures (Bonferroni)		t=0.392	0.005	0.695	No	6.000 vs. 9.000 in CNO
						All Pairwise Multiple Comparison Procedures (Bonferroni)		t=0.363	0.006	0.717	No	7.000 vs. 5.000 in CNO
						All Pairwise Multiple Comparison Procedures (Bonferroni)		t=0.305	0.006	0.761	No	10.000 vs. 8.000 in CNO
						All Pairwise Multiple Comparison Procedures (Bonferroni)		t=0.261	0.007	0.794	No	4.000 vs. 3.000 in CNO
						All Pairwise Multiple Comparison Procedures (Bonferroni)		t=0.261	0.009	0.794	No	6.000 vs. 8.000 in CNO

All Pairwise Multiple Comparison Procedures (Bonferroni)		t=0.261	0.01	0.794	No	0.000 vs. 1.000 in CNO
All Pairwise Multiple Comparison Procedures (Bonferroni)		t=0.247	0.013	0.805	No	8.000 vs. 7.000 in CNO
All Pairwise Multiple Comparison Procedures (Bonferroni)		t=0.131	0.017	0.896	No	8.000 vs. 9.000 in CNO
All Pairwise Multiple Comparison Procedures (Bonferroni)		t=0.116	0.025	0.908	No	9.000 vs. 7.000 in CNO
All Pairwise Multiple Comparison Procedures (Bonferroni)		t=0.0435	0.05	0.965	No	10.000 vs. 6.000 in CNO
All Pairwise Multiple Comparison Procedures (Bonferroni)		t=6.857	0.001	<0.001	Yes	9.000 vs. 0.000 in wash
All Pairwise Multiple Comparison Procedures (Bonferroni)		t=6.729	0.001	<0.001	Yes	9.000 vs. 1.000 in wash
All Pairwise Multiple Comparison Procedures (Bonferroni)		t=6.429	0.001	<0.001	Yes	10.000 vs. 0.000 in wash
All Pairwise Multiple Comparison Procedures (Bonferroni)		t=6.4	0.001	<0.001	Yes	8.000 vs. 0.000 in wash
All Pairwise Multiple Comparison Procedures (Bonferroni)		t=6.371	0.001	<0.001	Yes	7.000 vs. 0.000 in wash
All Pairwise Multiple Comparison Procedures (Bonferroni)		t=6.3	0.001	<0.001	Yes	10.000 vs. 1.000 in wash
All Pairwise Multiple Comparison Procedures (Bonferroni)		t=6.271	0.001	<0.001	Yes	8.000 vs. 1.000 in wash
All Pairwise Multiple Comparison Procedures (Bonferroni)		t=6.243	0.001	<0.001	Yes	7.000 vs. 1.000 in wash
All Pairwise Multiple Comparison Procedures (Bonferroni)		t=5.514	0.001	<0.001	Yes	9.000 vs. 2.000 in wash
All Pairwise Multiple Comparison Procedures (Bonferroni)		t=5.257	0.001	<0.001	Yes	6.000 vs. 0.000 in wash
All Pairwise Multiple Comparison Procedures (Bonferroni)		t=5.129	0.001	<0.001	Yes	9.000 vs. 3.000 in wash
All Pairwise Multiple Comparison Procedures (Bonferroni)		t=5.129	0.001	<0.001	Yes	6.000 vs. 1.000 in wash
All Pairwise Multiple Comparison Procedures (Bonferroni)		t=5.086	0.001	<0.001	Yes	10.000 vs. 2.000 in wash
All Pairwise Multiple Comparison Procedures (Bonferroni)		t=5.057	0.001	<0.001	Yes	8.000 vs. 2.000 in wash
All Pairwise Multiple Comparison Procedures (Bonferroni)		t=5.029	0.001	<0.001	Yes	7.000 vs. 2.000 in wash
All Pairwise Multiple Comparison Procedures (Bonferroni)		t=4.7	0.001	<0.001	Yes	10.000 vs. 3.000 in wash
All Pairwise Multiple Comparison Procedures (Bonferroni)		t=4.671	0.001	<0.001	Yes	8.000 vs. 3.000 in wash
All Pairwise Multiple Comparison Procedures (Bonferroni)		t=4.643	0.001	<0.001	Yes	7.000 vs. 3.000 in wash
All Pairwise Multiple Comparison Procedures (Bonferroni)		t=4.071	0.001	<0.001	Yes	5.000 vs. 0.000 in wash
All Pairwise Multiple Comparison Procedures (Bonferroni)		t=3.943	0.001	<0.001	Yes	5.000 vs. 1.000 in wash
All Pairwise Multiple Comparison Procedures (Bonferroni)		t=3.914	0.001	<0.001	Yes	6.000 vs. 2.000 in wash
All Pairwise Multiple Comparison Procedures (Bonferroni)		t=3.629	0.002	<0.001	Yes	4.000 vs. 0.000 in wash
All Pairwise Multiple Comparison Procedures (Bonferroni)		t=3.529	0.002	<0.001	Yes	6.000 vs. 3.000 in wash
All Pairwise Multiple Comparison Procedures (Bonferroni)		t=3.5	0.002	<0.001	Yes	4.000 vs. 1.000 in wash

All Pairwise Multiple Comparison Procedures (Bonferroni)		t=3.229	0.002	0.001	Yes	9.000 vs. 4.000 in wash
All Pairwise Multiple Comparison Procedures (Bonferroni)		t=2.8	0.002	0.005	No	10.000 vs. 4.000 in wash
All Pairwise Multiple Comparison Procedures (Bonferroni)		t=2.786	0.002	0.005	No	9.000 vs. 5.000 in wash
All Pairwise Multiple Comparison Procedures (Bonferroni)		t=2.771	0.002	0.005	No	8.000 vs. 4.000 in wash
All Pairwise Multiple Comparison Procedures (Bonferroni)		t=2.743	0.002	0.006	No	7.000 vs. 4.000 in wash
All Pairwise Multiple Comparison Procedures (Bonferroni)		t=2.729	0.002	0.006	No	5.000 vs. 2.000 in wash
All Pairwise Multiple Comparison Procedures (Bonferroni)		t=2.357	0.002	0.017	No	10.000 vs. 5.000 in wash
All Pairwise Multiple Comparison Procedures (Bonferroni)		t=2.343	0.002	0.018	No	5.000 vs. 3.000 in wash
All Pairwise Multiple Comparison Procedures (Bonferroni)		t=2.329	0.002	0.019	No	8.000 vs. 5.000 in wash
All Pairwise Multiple Comparison Procedures (Bonferroni)		t=2.3	0.002	0.02	No	7.000 vs. 5.000 in wash
All Pairwise Multiple Comparison Procedures (Bonferroni)		t=2.286	0.002	0.021	No	4.000 vs. 2.000 in wash
All Pairwise Multiple Comparison Procedures (Bonferroni)		t=1.9	0.003	0.054	No	4.000 vs. 3.000 in wash
All Pairwise Multiple Comparison Procedures (Bonferroni)		t=1.729	0.003	0.08	No	3.000 vs. 0.000 in wash
All Pairwise Multiple Comparison Procedures (Bonferroni)		t=1.629	0.003	0.099	No	6.000 vs. 4.000 in wash
All Pairwise Multiple Comparison Procedures (Bonferroni)		t=1.6	0.003	0.105	No	9.000 vs. 6.000 in wash
All Pairwise Multiple Comparison Procedures (Bonferroni)		t=1.6	0.003	0.105	No	3.000 vs. 1.000 in wash
All Pairwise Multiple Comparison Procedures (Bonferroni)		t=1.343	0.003	0.173	No	2.000 vs. 0.000 in wash
All Pairwise Multiple Comparison Procedures (Bonferroni)		t=1.214	0.004	0.218	No	2.000 vs. 1.000 in wash
All Pairwise Multiple Comparison Procedures (Bonferroni)		t=1.186	0.004	0.229	No	6.000 vs. 5.000 in wash
All Pairwise Multiple Comparison Procedures (Bonferroni)		t=1.171	0.004	0.235	No	10.000 vs. 6.000 in wash
All Pairwise Multiple Comparison Procedures (Bonferroni)		t=1.143	0.005	0.246	No	8.000 vs. 6.000 in wash
All Pairwise Multiple Comparison Procedures (Bonferroni)		t=1.114	0.005	0.258	No	7.000 vs. 6.000 in wash
All Pairwise Multiple Comparison Procedures (Bonferroni)		t=0.486	0.006	0.622	No	9.000 vs. 7.000 in wash
All Pairwise Multiple Comparison Procedures (Bonferroni)		t=0.457	0.006	0.643	No	9.000 vs. 8.000 in wash
All Pairwise Multiple Comparison Procedures (Bonferroni)		t=0.443	0.007	0.653	No	5.000 vs. 4.000 in wash
All Pairwise Multiple Comparison Procedures (Bonferroni)		t=0.429	0.009	0.664	No	9.000 vs. 10.000 in wash
All Pairwise Multiple Comparison Procedures (Bonferroni)		t=0.386	0.01	0.695	No	3.000 vs. 2.000 in wash
All Pairwise Multiple Comparison Procedures (Bonferroni)		t=0.129	0.013	0.896	No	1.000 vs. 0.000 in wash
All Pairwise Multiple Comparison Procedures (Bonferroni)		t=0.0571	0.017	0.954	No	10.000 vs. 7.000 in wash

All Pairwise Multiple Comparison Procedures (Bonferroni)		t=0.0286	0.025	0.977	No	10.000 vs. 8.000 in wash
All Pairwise Multiple Comparison Procedures (Bonferroni)		t=0.0286	0.05	0.977	No	8.000 vs. 7.000 in wash
All Pairwise Multiple Comparison Procedures (Bonferroni)		t = 0.703	0.017	0.484	No	Wash x Baseline in Stim 0
All Pairwise Multiple Comparison Procedures (Bonferroni)		t = 0.481	0.025	0.632	No	Wash x CNO in Stim 0
All Pairwise Multiple Comparison Procedures (Bonferroni)		t = 0.222	0.05	0.825	No	CNO x Baseline in Stim 0
All Pairwise Multiple Comparison Procedures (Bonferroni)		t = 0.851	0.017	0.398	No	Wash x Baseline in Stim 1
All Pairwise Multiple Comparison Procedures (Bonferroni)		t = 0.555	0.025	0.581	No	Wash x CNO in Stim 1
All Pairwise Multiple Comparison Procedures (Bonferroni)		t = 0.296	0.05	0.768	No	CNO x Baseline in Stim 1
All Pairwise Multiple Comparison Procedures (Bonferroni)		t = 0.757	0.017	0.515	No	Wash x CNO in Stim 2
All Pairwise Multiple Comparison Procedures (Bonferroni)		t = 0.571	0.025	0.623	No	Wash x Baseline in Stim 2
All Pairwise Multiple Comparison Procedures (Bonferroni)		t = 0.186	0.05	0.873	No	CNO x Baseline in Stim 2
All Pairwise Multiple Comparison Procedures (Bonferroni)		t = 0.567	0.017	0.572	No	Wash x Baseline in Stim 3
All Pairwise Multiple Comparison Procedures (Bonferroni)		t = 0.530	0.025	0.598	No	Wash x CNO in Stim 3
All Pairwise Multiple Comparison Procedures (Bonferroni)		t = 0.037	0.05	0.971	No	CNO x Baseline in Stim 3
All Pairwise Multiple Comparison Procedures (Bonferroni)		t = 1.949	0.017	0.055	No	Wash x CNO in Stim 4
All Pairwise Multiple Comparison Procedures (Bonferroni)		t = 1.258	0.025	0.212	No	Wash x Baseline in Stim 4
All Pairwise Multiple Comparison Procedures (Bonferroni)		t = 0.691	0.05	0.492	No	CNO x Baseline in Stim 4
All Pairwise Multiple Comparison Procedures (Bonferroni)		t = 1.640	0.017	0.105	No	Wash x CNO in Stim 5
All Pairwise Multiple Comparison Procedures (Bonferroni)		t = 1.011	0.025	0.315	No	Baseline x CNO in Stim 5
All Pairwise Multiple Comparison Procedures (Bonferroni)		t = 0.629	0.05	0.531	No	Wash x Baseline in Stim 5
All Pairwise Multiple Comparison Procedures (Bonferroni)		t = 2.047	0.017	0.044	No	CNO x Baseline in Stim 6
All Pairwise Multiple Comparison Procedures (Bonferroni)		t = 1.924	0.025	0.058	No	Wash x CNO in Stim 6
All Pairwise Multiple Comparison Procedures (Bonferroni)		t = 0.123	0.05	0.902	No	Wash x Baseline in Stim 6
Comparison Procedures		t = 3.318	0.017	0.001	Yes	Wash x CNO in Stim 7
All Pairwise Multiple Comparison Procedures		t = 2.898	0.025	0.005	Yes	Baseline x CNO in Stim 7
All Pairwise Multiple Comparison Procedures (Bonferroni)		t = 0.419	0.05	0.676	No	Wash x Baseline in Stim 7
All Pairwise Multiple Comparison Procedures (Bonferroni)		t = 3.133	0.017	0.003	Yes	Wash x CNO in Stim 8
All Pairwise Multiple Comparison Procedures (Bonferroni)		t = 2.664	0.025	0.01	Yes	Baseline x CNO in Stim 8
All Pairwise Multiple Comparison Procedures (Bonferroni)		t = 0.469	0.05	0.641	No	Wash x Baseline in Stim 8

						All Pairwise Multiple Comparison Procedures (Bonferroni)		t = 3.638	0.017	< 0.001	Yes	Wash x CNO in Stim 9
						All Pairwise Multiple Comparison Procedures (Bonferroni)		t = 2.812	0.025	0.006	Yes	Baseline x CNO in Stim 9
						All Pairwise Multiple Comparison Procedures (Bonferroni)		t = 0.826	0.05	0.411	No	Wash x Baseline in Stim 9
						All Pairwise Multiple Comparison Procedures (Bonferroni)		t = 2.898	0.017	0.005	Yes	Wash x CNO in Stim 10
						All Pairwise Multiple Comparison Procedures (Bonferroni)		t = 2.516	0.025	0.014	Yes	Baseline x CNO in Stim 10
						All Pairwise Multiple Comparison Procedures (Bonferroni)		t = 0.443	0.05	0.703	No	Wash x Baseline in Stim 10
DREADD Rotorod Learning	5	C	40		Assumed N≥30	**Mixed Factorial ANOVA**	**3,102**	**F = 6.188**	**0.05**	**0.002**	**Yes**	**Trial - MAIN EFFECT:The difference in the mean values among the different levels of Trial is greater than would be expected by chance after allowing for effects of differences in Condition.**
						**Mixed Factorial ANOVA**	**1,34**	**F = 0.055**	**0.05**	**0.816**	**No**	**Sex - NO MAIN EFFECT**
						**Mixed Factorial ANOVA**	**2,34**	**F = 2.264**	**0.05**	**0.119**	**No**	**Condition-NO MAIN EFFECT**
						Mixed Factorial ANOVA	3,102	F = 0.558	0.05	0.602	No	Trial x Sex - NO INTERACTION
						**Mixed Factorial ANOVA**	**6,102**	**F = 3.176**	**0.05**	**0.013**	**Yes**	**Trial x Condition - INTERACTION: The differences between Trail are effected by the level of Condition**
						Mixed Factorial ANOVA	2,34	F = 0.027	0.05	0.973	No	Sex x Condition - NO INTERACTION
						Mixed Factorial ANOVA	6,102	F = 1.034	0.05	0.057	No	Trial x Sex x Condition - NO INTERACTION
						All Pairwise Multiple Comparison Procedures (Holm-Sidak method)		t = 3.408	0.009	< 0.001	Yes	Recovery vs Withdrawal in EtOH DREADD +
						All Pairwise Multiple Comparison Procedures (Holm-Sidak method)		t = 3.209	0.01	0.002	Yes	Baseline vs Withdrawal in EtOH DREADD +
						All Pairwise Multiple Comparison Procedures (Holm-Sidak method)		t = 2.092	0.013	0.039	No	Intoxication vs Withdrawal in EtOH DREADD +
						All Pairwise Multiple Comparison Procedures (Holm-Sidak method)		t = 1.316	0.017	0.191	No	Recovery vs Intoxication in EtOH DREADD +
						All Pairwise Multiple Comparison Procedures (Holm-Sidak method)		t = 1.118	0.025	0.266	No	Baseline vs Intoxication in EtOH DREADD +
						All Pairwise Multiple Comparison Procedures (Holm-Sidak method)		t = 0.199	0.05	0.843	No	Recovery vs Baseline in EtOH DREADD +
						All Pairwise Multiple Comparison Procedures (Holm-Sidak method)		t = 3.688	0.009	< 0.001	Yes	Baseline vs Withdrawal in EtOH DREADD -
						All Pairwise Multiple Comparison Procedures (Holm-Sidak method)		t = 2.731	0.01	0.007	Yes	Baseline vs Intoxication in EtOH DREADD -
						All Pairwise Multiple Comparison Procedures (Holm-Sidak method)		t = 2.438	0.013	0.016	No	Recovery vs Withdrawal in EtOH DREADD -
						All Pairwise Multiple Comparison Procedures (Holm-Sidak method)		t = 1.481	0.017	0.141	No	Recovery vs Intoxication in EtOH DREADD -
						All Pairwise Multiple Comparison Procedures (Holm-Sidak method)		t = 1.250	0.025	0.214	No	Baseline vs Recovery in EtOH DREADD -
						All Pairwise Multiple Comparison Procedures (Holm-Sidak method)		t = 0.957	0.05	0.341	No	Intoxication vs Withdrawal in EtOH DREADD -
						All Pairwise Multiple Comparison Procedures (Holm-Sidak method)		t = 0.773	0.009	0.441	No	Recovery vs Withdrawal in Air DREADD
						All Pairwise Multiple Comparison Procedures (Holm-Sidak method)		t = 0.527	0.01	0.599	No	Baseline vs Withdrawal in Air DREADD
						All Pairwise Multiple Comparison Procedures (Holm-Sidak method)		t = 0.410	0.013	0.683	No	Intoxication vs Withdrawal in Air DREADD
						All Pairwise Multiple Comparison Procedures (Holm-Sidak method)		t = 0.363	0.017	0.717	No	Withdrawal vs Intoxication in Air DREADD

						All Pairwise Multiple Comparison Procedures (Holm-Sidak method)		t = 0.246	0.025	0.806	No	Recovery vs Baseline in Air DREADD
						All Pairwise Multiple Comparison Procedures (Holm-Sidak method)		t = 0.117	0.05	0.907	No	Recovery vs Baseline in Air DREADD
						All Pairwise Multiple Comparison Procedures (Holm-Sidak method)		t = 2.372	0.017	0.02	No	Air DREADD vs EtOH DREADD - in Intoxication
						All Pairwise Multiple Comparison Procedures (Holm-Sidak method)		t = 1.178	0.025	0.242	No	EtOH DREADD + vs EtOH DREADD - in Intoxication
						All Pairwise Multiple Comparison Procedures (Holm-Sidak method)		t = 1.012	0.05	0.314	No	Air DREADD vs EtOH DREADD + in Intoxication
						**All Pairwise Multiple Comparison Procedures (Holm-Sidak method)**		**t = 3.422**	**0.017**	**<0.001**	**Yes**	**EtOH DREADD - vs Air DREADD in Withdrawal**
						**All Pairwise Multiple Comparison Procedures (Holm-Sidak method)**		**t = 3.019**	**0.025**	**0.003**	**Yes**	**EtOH DREADD + vs Air DREADD in Withdrawal**
						All Pairwise Multiple Comparison Procedures (Holm-Sidak method)		t = 0.349	0.05	0.728	No	EtOH DREADD - vs EtOH DREADD + in Withdrawal
						All Pairwise Multiple Comparison Procedures (Holm-Sidak method)		t = 1.057	0.017	0.293	No	EtOH DREADD - vs EtOH DREADD + in Recovery
						All Pairwise Multiple Comparison Procedures (Holm-Sidak method)		t = 0.907	0.025	0.367	No	EtOH DREADD - vs Air DREADD in Recovery
						All Pairwise Multiple Comparison Procedures (Holm-Sidak method)		t = 0.314	0.05	0.754	No	Air DREADD vs EtOH DREADD + in Recovery
						Air DREADD + and Air DREADD - Comparison						
						Two Way RM ANOVA	3,36	F = 0.0265	0.05	0.002	No	Trial - NO MAIN EFFECT
						Two Way RM ANOVA	1,36	F = 0.198	0.05	0.816	No	Condition-NO MAIN EFFECT
						**Two Way RM ANOVA**	**3,36**	**F = 0.154**	**0.05**	**0.119**	**No**	**Condition x Trial-NO INTERACTION**
						**Mixed Factorial ANOVA**	**2,68**	**F = 12.070**	**0.05**	**<0.001**	**Yes**	**Trial - MAIN EFFECT:The difference in the mean values among the different levels of Trial is greater than would be expected by chance after allowing for effects of differences in Condition.**
						**Mixed Factorial ANOVA**	**1,34**	**F = 0.062**	**0.05**	**0.804**	**No**	**Sex - NO MAIN EFFECT**
						**Mixed Factorial ANOVA**	**2,34**	**F = 0.902**	**0.05**	**0.415**	**No**	**Condition-NO MAIN EFFECT**
						**Mixed Factorial ANOVA**	**2,68**	**F = 0.038**	**0.05**	**0.92**	**No**	**Trial x Sex - NO INTERACTION**
						**Mixed Factorial ANOVA**	**2,68**	**F = 3.347**	**0.05**	**0.028**	**Yes**	**Trial x Condition - The differences between Trials is effected by the level of condition**
						**Mixed Factorial ANOVA**	**2,34**	**F = 0.525**	**0.05**	**0.596**	**No**	**Sex x Condition - NO INTERACTION**
						**Mixed Factorial ANOVA**	**2,68**	**F = 1.018**	**0.05**	**0.056**	**No**	**Trial x Sex x Condition - NO INTERACTION**
						All Pairwise Multiple Comparison Procedures (Holm-Sidak method)		t = 3.672	0.017	<0.001	Yes	Recovery vs Withdrawal in EtOH DREADD +
						All Pairwise Multiple Comparison Procedures (Holm-Sidak method)		t = 2.352	0.025	0.021	No	Baseline vs Withdrawal in EtOH DREADD +
						All Pairwise Multiple Comparison Procedures (Holm-Sidak method)		t = 1.320	0.05	0.191	No	Recovery vs Baseline in EtOH DREADD +
						All Pairwise Multiple Comparison Procedures (Holm-Sidak method)		t = 3.868	0.017	< 0.001	Yes	Baseline vs Withdrawal in EtOH DREADD -
DREADD Rotorod Performance	5	D	40		Assumed N≥30	All Pairwise Multiple Comparison Procedures (Holm-Sidak method)		t = 3.338	0.025	0.001	Yes	Recovery vs Withdrawal in EtOH DREADD -
						All Pairwise Multiple Comparison Procedures (Holm-Sidak method)		t = 0.530	0.05	0.598	No	Baseline vs Recovery in EtOH DREADD -
						All Pairwise Multiple Comparison Procedures (Holm-Sidak method)		t = 0.925	0.017	0.358	No	Baseline vs Withdrawal in Air DREADD
						All Pairwise Multiple Comparison Procedures (Holm-Sidak method)		t = 0.891	0.025	0.376	No	Recovery vs Baseline in EtOH DREADD
						All Pairwise Multiple Comparison Procedures (Holm-Sidak method)		t = 0.0339	0.05	0.973	No	Recovery vs Withdrawal in Air DREADD

						**All Pairwise Multiple Comparison Procedures (Holm-Sidak method)**		**t = 3.091**	**0.017**	**0.003**	**Yes**	**Air DREADD vs EtOH DREADD - in Withdrawal**
						**All Pairwise Multiple Comparison Procedures (Holm-Sidak method)**		**t = 1.633**	**0.025**	**0.106**	**No**	**Air DREADD vs EtOH DREADD + in Withdrawal**
						All Pairwise Multiple Comparison Procedures (Holm-Sidak method)		t = 1.262	0.05	0.21	No	EtOH DREADD + vs EtOH DREADD - in Withdrawal
						All Pairwise Multiple Comparison Procedures (Holm-Sidak method)		t = 1.875	0.017	0.064	No	Air DREADD vs EtOH DREADD + in Recovery
						All Pairwise Multiple Comparison Procedures (Holm-Sidak method)		t = 1.541	0.025	0.127	No	EtOH DREADD - vs EtOH DREADD + in Recovery
						All Pairwise Multiple Comparison Procedures (Holm-Sidak method)		t = 0.0958	0.05	0.924	No	Air DREADD vs EtOH DREADD - in Recovery
						Air DREADD + and Air DREADD - Comparison						
						Two Way RM ANOVA	2	F = 0.702	0.05	0.502	No	Trial - NO MAIN EFFECT
						Two Way RM ANOVA	1	F = 1.016	0.05	0.327	No	Condition-NO MAIN EFFECT
						**Two Way RM ANOVA**	**2**	**F = 0.617**	**0.05**	**0.545**	**No**	**Condition x Trial-NO INTERACTION**
						**Mixed Factorial ANOVA**	**5,115**	**F = 10.019**	**0.05**	**< 0.001**	**Yes**	**Frequency - MAIN EFFECT: The difference in the mean values among the different levels of frequency is greater than would be expected by chance after allowing for effects of differences in Condition and sex.**
						**Mixed Factorial ANOVA**	**1,23**	**F = 0.000**	**0.05**	**0.989**	**No**	**Sex - NO MAIN EFFECT**
						**Mixed Factorial ANOVA**	**3,23**	**F= 1.480**	**0.05**	**0.246**	**No**	**Condition - NO MAIN EFFECT**
						**Mixed Factorial ANOVA**	**5,115**	**F = 0.418**	**0.05**	**0.692**	**No**	**Frequency x Sex - NO INTERACTION**
						**Mixed Factorial ANOVA**	**15,115**	**F = 0.725**	**0.05**	**0.653**	**No**	**Frequency x Condition - NO INTERACTION**
						**Mixed Factorial ANOVA**	**3,23**	**F = 0.515**	**0.05**	**0.676**	**No**	**Sex x Condition - NO INTERACTION**
						**Mixed Factorial ANOVA**	**15,115**	**F = 0.338**	**0.05**	**0.934**	**No**	**Frequency x Sex x Condition - NO INTERACTION**
						All Pairwise Multiple Comparison Procedures (Holm-Sidak method)		t=4.184	0.003	<0.001	Yes	20-40 vs. 100-120
						All Pairwise Multiple Comparison Procedures (Holm-Sidak method)		t=3.874	0.004	<0.001	Yes	20-40 vs. 80-100
						All Pairwise Multiple Comparison Procedures (Holm-Sidak method)		t=3.118	0.004	0.002	Yes	0-20 vs. 100-120
						All Pairwise Multiple Comparison Procedures (Holm-Sidak method)		t=2.873	0.004	0.005	No	40-60 vs. 100-120
						All Pairwise Multiple Comparison Procedures (Holm-Sidak method)		t=2.808	0.005	0.006	No	0-20 vs. 80-100
						All Pairwise Multiple Comparison Procedures (Holm-Sidak method)		t=2.563	0.005	0.012	No	40-60 vs. 80-100
DREADD Withdrawal USVs	5	E-F		31		Assumed N≥30	All Pairwise Multiple Comparison Procedures (Holm-Sidak method)		t=2.176	0.006	0.032	No	20-40 vs. 60-80
						All Pairwise Multiple Comparison Procedures (Holm-Sidak method)		t=2.008	0.006	0.047	No	60-80 vs. 100-120
						All Pairwise Multiple Comparison Procedures (Holm-Sidak method)		t=1.698	0.007	0.092	No	60-80 vs. 80-100
						All Pairwise Multiple Comparison Procedures (Holm-Sidak method)		t=1.311	0.009	0.192	No	20-40 vs. 40-60
						All Pairwise Multiple Comparison Procedures (Holm-Sidak method)		t=1.11	0.01	0.269	No	0-20 vs. 60-80
						All Pairwise Multiple Comparison Procedures (Holm-		t=1.065	0.013	0.289	No	20-40 vs. 0-20
						All Pairwise Multiple Comparison Procedures (Holm-		t=0.865	0.017	0.389	No	40-60 vs. 60-80
						All Pairwise Multiple Comparison Procedures (Holm-		t=0.31	0.025	0.757	No	80-100 vs. 100-120
						All Pairwise Multiple Comparison Procedures (Holm-Sidak method)		t=0.245	0.05	0.807	No	0-20 vs. 40-60
						All Pairwise Multiple Comparison Procedures (Holm-Sidak method)		t=0.474	0.05	0.998	No	EtOH - vs EtOH +

						All Pairwise Multiple Comparison Procedures (Holm-Sidak method)		t=0.602	0.05	0.992	No	EtOH + vs Air +
						All Pairwise Multiple Comparison Procedures (Holm-Sidak method)		t=-1.598	0.05	0.547	No	EtOH + vs Air -
						All Pairwise Multiple Comparison Procedures (Holm-Sidak method)		t=0.088	0.05	1.000	No	EtOH - vs Air +
						**All Pairwise Multiple Comparison Procedures (Holm-Sidak method)**		**t=-1.943**	**0.05**	**0.329**	**No**	**EtOH - vs Air -**
						All Pairwise Multiple Comparison Procedures (Holm-Sidak method)		t=-2.136	0.05	0.235	No	Air + vs Air -
	**Figure #**	**Figure Letter**	**Animal #**	**Cell #**	**normality**	**Test Type**	**DF**	**F- / T- / Z-Value**	**Critical Level**	**P-value**	**Significant?**	**Comments**
5uM C6 sIPSC Freq	6	B	**5**	**13**	**Yes**	**One Sample t-test**	**5**	**t = 0.439**	**0.050**	**0.679**	**No**	**Cerebellum Baseline vs 5uM C6 Freq**
						**One Sample t-test**	**6**	**t= -0.332**	**0.050**	**0.751**	**No**	**Hippocampus Baseline vs 5uM C6 Freq**
5uM C6 Amplitude Change	6	C	5	13	Yes	**One Sample t-test**	**5**	**t = -3.791**	**0.050**	**0.013**	**Yes**	**Cerebellum Baseline vs 5uM C6 Amplitude**
						**One Sample t-test**	**6**	**t = 0.412**	**0.050**	**0.695**	**No**	**Hippocampus Baseline vs 5uM C6 Amplitude**
5uM C6 Rise Time Change	6	D	5	13	Yes	**One Sample t-test**	**5**	**t = -0.218**	**0.050**	**0.836**	**No**	**Cerebellum Baseline vs 5uM C6 Rise Time**
						**One Sample t-test**	**6**	**t= -0.989**	**0.050**	**0.361**	**No**	**Hippocampus Baseline vs 5uM C6 Rise Time**
5uM C6 Decay Time Change	6	E	5	13	Yes	**One Sample t-test**	**5**	**t = 0.0853**	**0.050**	**0.935**	**No**	**Cerebellum Baseline vs 5uM C6 Decay Time**
						**One Sample t-test**	**6**	**t= -0.366**	**0.050**	**0.727**	**No**	**Hippocampus Baseline vs 5uM C6 Decay Time**
Cerebellar Drug Induced Current	6	F	11	25	Yes	**One Sample t-test**	**9**	**t = 1.482**	**0.050**	**0.173**	**No**	**1.5uM C6 vs Baseline Holding Current**
						**One Sample t-test**	**8**	**t = 3.380**	**0.050**	**0.010**	**Yes**	**3uM C6 vs Baseline Holding Current**
						**One Sample t-test**	**6**	**t = 3.628**	**0.050**	**0.015**	**Yes**	**5uM C6 vs Baseline Holding Current**
						**One Sample t-test**	**25**	**t = -8.746**	**0.050**	**< 0.001**	**Yes**	**10uM Gabazine vs Baseline Holding Current**
C6 Tonic Potentiation	6	G	11	23	No	**One Sample t-test**	**7**	**t =0.352**	**0.05**	**0.735**	**No**	**1.5uM C6**
						**Mann-Whitney Rank Sum**	**8,8**	**T=92**	**0.05**	**0.01**	**Yes**	**1.5uM C6**
					Yes	**One Sample t-test**	**6**	**t = -2.655**	**0.05**	**0.038**	**Yes**	**3uM C6**
					Yes	**One Sample t-test**	**6**	**t = -2.560**	**0.05**	**0.043**	**Yes**	**5uM C6**
Hippocampal Drug Induced Current	6	H	2	7	Yes	**One Sample t-test**	**7**	**t = 0.396**	**0.050**	**0.705**	**No**	**Baseline vs 5uM C6 Holding Current**
						**One Sample t-test**	**7**	**t= 0.311**	**0.050**	**0.768**	**No**	**Baseline vs 10uM Gabazine Holding Current**
	**Figure #**	**Figure Letter**	**Animal #**	**Cell #**	**normality**	**Test Type**	**DF**	**F- / T- / Z-Value**	**Critical Level**	**P-value**	**Significant?**	**Comments**
						**Mixed Factorial ANOVA**	**3,72**	**F = 17.654**	**0.05**	**< 0.001**	**Yes**	**Trial - MAIN EFFECT:The difference in the mean values among the different**
						**Mixed Factorial ANOVA**	**1,24**	**F = 0.011**	**0.05**	**0.917**	**No**	**Sex - NO MAIN EFFECT**
						**Mixed Factorial ANOVA**	**3,24**	**F = 5.158**	**0.05**	**0.007**	**Yes**	**Condition- MAIN EFFECT:The difference in the mean values among the different levels of Condition is greater than would be expected by chance after allowing for effects of differences in Trial and Sex**
						**Mixed Factorial ANOVA**	**3,72**	**F = 1.000**	**0.05**	**0.382**	**No**	**Trial x Sex - NO INTERACTION**
						**Mixed Factorial ANOVA**	**9,72**	**F = 4.532**	**0.05**	**< 0.001**	**Yes**	**Trial x Condition - INTERACTION: The differences between Trail are effected by the level of Condition**
						**Mixed Factorial ANOVA**	**3,24**	**F = 1.737**	**0.05**	**0.186**	**No**	**Sex x Condition - NO INTERACTION**
						**Mixed Factorial ANOVA**	**9,72**	**F = 1.166**	**0.05**	**0.338**	**No**	**Trial x Sex x Condition - NO INTERACTION**
						All Pairwise Multiple Comparison Procedures (Holm-Sidak method)		t = 1.379	0.009	0.172	No	Intoxication vs Baselinel in Air
						All Pairwise Multiple Comparison Procedures (Holm-Sidak method)		t = 1.085	0.01	0.281	No	Baseline vs Withdrawal in Air
						All Pairwise Multiple Comparison Procedures (Holm-Sidak method)		t =0.943	0.013	0.348	No	Recovery vs Baseline in Air
						All Pairwise Multiple Comparison Procedures (Holm-Sidak method)		t = 0.436	0.017	0.664	No	Recovery vs Intoxication in Air

Compoud 6 Rotorod Learning	7	A		32		Assumed N≥30	All Pairwise Multiple Comparison Procedures (Holm-Sidak method)		t = 0.294	0.025	0.77	No	Intoxication vs Withdrawal in Air
							All Pairwise Multiple Comparison Procedures (Holm-Sidak method)		t = 0.142	0.05	0.887	No	Recovery vs Withdrawal in Air
							All Pairwise Multiple Comparison Procedures (Holm-Sidak method)		t = 5.794	0.009	< 0.001	Yes	Recovery vs Withdrawal in EtOH
							All Pairwise Multiple Comparison Procedures (Holm-Sidak method)		t = 4.163	0.01	< 0.001	Yes	Recovery vs Intoxication in EtOH
							All Pairwise Multiple Comparison Procedures (Holm-Sidak method)		t = 3.266	0.013	0.002	Yes	Baseline vs Withdrawal in EtOH
							All Pairwise Multiple Comparison Procedures (Holm-Sidak method)		t = 2.527	0.017	0.013	Yes	Baseline vs Recovery in EtOH
							All Pairwise Multiple Comparison Procedures (Holm-Sidak method)		t = 1.635	0.025	0.106	No	Baseline vs Intoxicaition in EtOH
							All Pairwise Multiple Comparison Procedures (Holm-Sidak method)		t = 1.631	0.05	0.107	No	Intoxication vs Withdrawal in EtOH
							All Pairwise Multiple Comparison Procedures (Holm-Sidak method)		t = 4.334	0.009	< 0.001	Yes	Recovery vs Baseline in Air + C6
							All Pairwise Multiple Comparison Procedures (Holm-Sidak method)		t = 3.694	0.01	< 0.001	Yes	Recovery vs Withdrawal in Air + C6
							All Pairwise Multiple Comparison Procedures (Holm-Sidak method)		t = 2.560	0.013	0.012	Yes	Baseline vs Intoxication in Air + C6
							All Pairwise Multiple Comparison Procedures (Holm-Sidak method)		t = 1.920	0.017	0.058	No	Intoxication vs Withdrawal in Air + C6
							All Pairwise Multiple Comparison Procedures (Holm-Sidak method)		t = 1.774	0.025	0.08	No	Recovery vs Intoxicaiton in Air + C6
							All Pairwise Multiple Comparison Procedures (Holm-Sidak method)		t = 0.640	0.05	0.524	No	Baseline vs Withdrawal in Air + C6
							All Pairwise Multiple Comparison Procedures (Holm-Sidak method)		t = 4.691	0.009	< 0.001	Yes	Withdrawal vs Recovery in EtOH + C6
							All Pairwise Multiple Comparison Procedures (Holm-Sidak method)		t = 3.706	0.01	< 0.001	Yes	Baseline vs Withdrawal in EtOH + C6
							All Pairwise Multiple Comparison Procedures (Holm-Sidak method)		t = 2.716	0.013	0.008	Yes	Recovery vs Intoxciation in EtOH + C6
							All Pairwise Multiple Comparison Procedures (Holm-Sidak method)		t = 1.975	0.017	0.052	No	Withdrawal vs Intoxciation in EtOH + C6
							All Pairwise Multiple Comparison Procedures (Holm-Sidak method)		t = 1.731	0.025	0.087	No	Baseline vs Intoxciation in EtOH + C6
							All Pairwise Multiple Comparison Procedures (Holm-Sidak method)		t = 0.985	0.05	0.327	No	Baseline vs Recovery in EtOH + C6
							All Pairwise Multiple Comparison Procedures (Holm-Sidak method)		t = 3.487	0.009	<0.001	Yes	Air + C6 vs EtOH + C6 in Intoxication
							All Pairwise Multiple Comparison Procedures (Holm-Sidak method)		t = 3.476	0.01	<0.001	Yes	Air + C6 vs EtOH in Intoxication
							All Pairwise Multiple Comparison Procedures (Holm-Sidak method)		t = 2.543	0.013	0.013	No	Air vs EtOH + C6 in Intoxication
							All Pairwise Multiple Comparison Procedures (Holm-Sidak method)		t = 2.449	0.017	0.016	Yes	Air vs EtOH in Intoxication
							All Pairwise Multiple Comparison Procedures (Holm-Sidak method)		t = 1.355	0.025	0.179	No	Air + C6 vs Air in Intoxication
							All Pairwise Multiple Comparison Procedures (Holm-		t = 0.422	0.05	0.674	No	EtOH + C6 vs EtOH in Intoxication
							**All Pairwise Multiple Comparison Procedures (Holm-Sidak method)**		**t = 4.131**	**0.009**	**< 0.001**	**Yes**	**Air vs EtOH + C6 in Withdrawal**
							**All Pairwise Multiple Comparison Procedures (Holm-Sidak method)**		**t = 3.536**	**0.01**	**< 0.001**	**Yes**	**Air vs EtOH in Withdrawal**
							All Pairwise Multiple Comparison Procedures (Holm-Sidak method)		t =3.531	0.013	< 0.001	Yes	Air + C6 vs EtOH + C6 in Withdrawal

						All Pairwise Multiple Comparison Procedures (Holm-Sidak method)		t = 2.880	0.017	0.005	Yes	Air + C6 vs EtOH in Withdrawal
						All Pairwise Multiple Comparison Procedures (Holm-Sidak method)		t = 1.068	0.025	0.288	No	EtOH vs EtOH + C6 in Withdrawal
						All Pairwise Multiple Comparison Procedures (Holm-Sidak method)		t = 0.183	0.05	0.855	No	Air vs Air + C6in Withdrawal
						All Pairwise Multiple Comparison Procedures (Holm-Sidak method)		t = 3.273	0.009	0.002	Yes	Air + C6 vs Air in Recovery
						All Pairwise Multiple Comparison Procedures (Holm-Sidak method)		t = 2.721	0.01	0.008	Yes	Air + C6 vs EtOH+ C6 in Recovery
						All Pairwise Multiple Comparison Procedures (Holm-Sidak method)		t = 2.185	0.013	0.034	No	Air + C6 vs EtOH in Recovery
						All Pairwise Multiple Comparison Procedures (Holm-Sidak method)		t = 1.287	0.017	0.202	No	Air vs EtOH in Recovery
						All Pairwise Multiple Comparison Procedures (Holm-Sidak method)		t = 0.883	0.025	0.38	No	EtOH vs EtOH + C6 in Recovery
						All Pairwise Multiple Comparison Procedures (Holm-Sidak method)		t =0.231	0.05	0.818	No	Air vs EtOH + C6 in Recovery
C6 Rotorod Performance	7	B		32		Assumed N≥30	**Mixed Factorial ANOVA**	**2,48**	**F = 40.214**	**0.05**	**< 0.001**	**Yes**	**Trial - MAIN EFFECT:The difference in the mean values among the different levels of Trial is greater than would be expected by chance after allowing for effects of differences in Condition and sex.**
						**Mixed Factorial ANOVA**	**1,24**	**F = 3.279**	**0.05**	**0.083**	**No**	**Sex - NO MAIN EFFECT**
						**Mixed Factorial ANOVA**	**3,24**	**F = 3.251**	**0.05**	**0.039**	**Yes**	**Condition-MAIN EFFECT: The differences in the mean values among the different levels of condition is greater than would be expected by chance after allowing for effects of trial and sex.**
						**Mixed Factorial ANOVA**	**2,48**	**F = 2.111**	**0.05**	**0.132**	**No**	**Trial x Sex - NO INTERACTION**
						**Mixed Factorial ANOVA**	**6,48**	**F = 5.549**	**0.05**	**< 0.001**	**Yes**	**Trial x Condition - INTERACTION: The differences in trial are effected by condition**
						**Mixed Factorial ANOVA**	**3,24**	**F = 1.321**	**0.05**	**0.291**	**No**	**Sex x Condition - NO INTERACTION**
						**Mixed Factorial ANOVA**	**6,48**	**F = 1.262**	**0.05**	**0.292**	**No**	**Trial x Sex x Condition - NO INTERACTION**
						All Pairwise Multiple Comparison Procedures (Holm-Sidak method)		t = 2.330	0.017	0.023	No	Recovery vs Baseline in Air
						All Pairwise Multiple Comparison Procedures (Holm-Sidak method)		t = 1.481	0.025	0.144	No	Baseline vs Withdrawal in Air
						All Pairwise Multiple Comparison Procedures (Holm-Sidak method)		t = 0.849	0.05	0.399	No	Recovery vs Withdrawal in Air
						All Pairwise Multiple Comparison Procedures (Holm-Sidak method)		t = 5.871	0.017	< 0.001	Yes	Recovery vs Withdrawal in EtOH
						All Pairwise Multiple Comparison Procedures (Holm-Sidak method)		t = 3.048	0.025	0.004	Yes	Recovery vs Baseline in EtOH
						All Pairwise Multiple Comparison Procedures (Holm-Sidak method)		t = 2.823	0.05	0.007	Yes	Baseline vs Withdrawal in EtOH
						All Pairwise Multiple Comparison Procedures (Holm-Sidak method)		t = 5.179	0.017	<0.001	Yes	Recovery vs Withdrawal in Air + C6
						All Pairwise Multiple Comparison Procedures (Holm-Sidak method)		t = 3.238	0.025	0.002	Yes	Baseline vs Withdrawal in Air + C6
						All Pairwise Multiple Comparison Procedures (Holm-Sidak method)		t = 1.941	0.05	0.057	No	Baseline vs Recovery in Air + C6
						All Pairwise Multiple Comparison Procedures (Holm-Sidak method)		t = 5.200	0.017	<0.001	Yes	Withdrawal vs Recovery in EtOH + C6
						All Pairwise Multiple Comparison Procedures (Holm-Sidak method)		t = 4.578	0.025	<0.001	Yes	Baseline vs Withdrawal in EtOH + C6
						All Pairwise Multiple Comparison Procedures (Holm-Sidak method)		t = 0.622	0.05	0.536	No	Recovery vs Baseline in EtOH + C6
						**All Pairwise Multiple Comparison Procedures (Holm-Sidak method)**		**t = 5.307**	**0.009**	**<0.001**	**Yes**	**Air vs EtOH + C6 in Withdrawal**

						All Pairwise Multiple Comparison Procedures (Holm-Sidak method)		t = 4.065	0.01	<0.001	Yes	Air vs Air + C6 in Withdrawal
						**All Pairwise Multiple Comparison Procedures (Holm-Sidak method)**		**t = 3.569**	**0.013**	**<0.001**	**Yes**	**Air vs EtOH in Withdrawal**
						All Pairwise Multiple Comparison Procedures (Holm-Sidak method)		t = 2.216	0.017	0.03	No	EtOH vs EtOH + C6 in Withdrawal
						All Pairwise Multiple Comparison Procedures (Holm-Sidak method)		t = 1.111	0.025	0.27	No	EtOH + C6 vs Air - in Withdrawal
						All Pairwise Multiple Comparison Procedures (Holm-Sidak method)		t = 0.974	0.05	0.333	No	EtOH vs Air + C6 in Withdrawal
						All Pairwise Multiple Comparison Procedures (Holm-Sidak method)		t = 1.612	0.009	0.111	No	EtOH vs EtOH + C6 in Recovery
						All Pairwise Multiple Comparison Procedures (Holm-Sidak method)		t = 1.096	0.01	0.277	No	Air vs EtOH + C6 in Recovery
						All Pairwise Multiple Comparison Procedures (Holm-Sidak method)		t = 1.094	0.013	0.278	No	Air + C6 vs EtOH + C6 in Recovery
						All Pairwise Multiple Comparison Procedures (Holm-Sidak method)		t = 0.595	0.017	0.554	No	Air vs EtOH in Recovery
						All Pairwise Multiple Comparison Procedures (Holm-Sidak method)		t = 0.389	0.025	0.698	No	EtOH vs Air + C6 in Recovery
						All Pairwise Multiple Comparison Procedures (Holm-Sidak method)		t = 0.126	0.05	0.9	No	Air + C6 vs Air in Recovery
Compound 6 Withdrawal USVs	7	C-D	31		Assumed N≥30	**Mixed Factorial ANOVA**	**5,115**	**F = 16.378**	**0.05**	**< 0.001**	**Yes**	**Frequency - MAIN EFFECT: The difference in the mean values among the different levels of frequency is greater than would be expected by chance after allowing for effects of differences in Condition and sex.**
						**Mixed Factorial ANOVA**	**1,23**	**F = 6.818**	**0.05**	**0.016**	**Yes**	**Sex - MAIN EFFECT: The difference in the mean values among the different levels of sex is greater than would be expected by chance after allowing for effects of differences in Condition and frequency**
						**Mixed Factorial ANOVA**	**3,23**	**F= 2.308**	**0.05**	**0.103**	**No**	**Condition - NO MAIN EFFECT**
						**Mixed Factorial ANOVA**	**5,115**	**F = 0.564**	**0.05**	**0.604**	**No**	**Frequency x Sex - NO INTERACTION**
						**Mixed Factorial ANOVA**	**15,115**	**F = 2.815**	**0.05**	**0.013**	**Yes**	**Frequency x Condition - INTERACTION: Differences in Frequency are effected by the level of condition**
						**Mixed Factorial ANOVA**	**3,23**	**F = 2.043**	**0.05**	**0.136**	**No**	**Sex x Condition - NO INTERACTION**
						**Mixed Factorial ANOVA**	**15,115**	**F = 1.063**	**0.05**	**0.4**	**No**	**Frequency x Sex x Condition - NO INTERACTION**
						All Pairwise Multiple Comparison Procedures (Holm-Sidak method)		t=4.003	0.003	<0.001	Yes	0-20 vs. 80-100
						All Pairwise Multiple Comparison Procedures (Holm-Sidak method)		t=3.935	0.004	<0.001	Yes	20-40 vs. 80-100
						All Pairwise Multiple Comparison Procedures (Holm-Sidak method)		t=3.910	0.004	<0.001	Yes	0-20 vs. 100-120
						All Pairwise Multiple Comparison Procedures (Holm-Sidak method)		t=3.842	0.004	<0.001	Yes	20-40 vs. 100-120
						All Pairwise Multiple Comparison Procedures (Holm-Sidak method)		t=3.555	0.005	<0.001	Yes	0-20 vs. 60-80
						All Pairwise Multiple Comparison Procedures (Holm-Sidak method)		t=3.487	0.005	<0.001	Yes	20-40 vs. 60-80
						All Pairwise Multiple Comparison Procedures (Holm-Sidak method)		t=2.984	0.006	0.003	Yes	0-20 vs. 40-60
						All Pairwise Multiple Comparison Procedures (Holm-Sidak method)		t=2.915	0.006	0.004	Yes	20-40 vs. 40-60
						All Pairwise Multiple Comparison Procedures (Holm-Sidak method)		t=1.020	0.007	0.309	No	40-60 vs. 80-100

						All Pairwise Multiple Comparison Procedures (Holm-Sidak method)		t=0.926	0.009	0.356	No	40-60 vs. 100-120
						All Pairwise Multiple Comparison Procedures (Holm-Sidak method)		t=0.571	0.01	0.569	No	40-60 vs. 60-80
						All Pairwise Multiple Comparison Procedures (Holm-Sidak method)		t=0.449	0.013	0.654	No	60-80 vs. 80-100
						All Pairwise Multiple Comparison Procedures (Holm-Sidak method)		t=0.355	0.017	0.723	No	60-80 vs. 100-120
						All Pairwise Multiple Comparison Procedures (Holm-Sidak method)		t=0.0.933	0.025	0.926	No	100-120 vs. 80-100
						All Pairwise Multiple Comparison Procedures (Holm-Sidak method)		t=0.0682	0.05	0.946	No	0-20 vs. 20-40
						**All Pairwise Multiple Comparison Procedures (Holm-Sidak method)**		**t=3.275**	**0.009**	**0.001**	**Yes**	**Air + C6 vs Air**
						All Pairwise Multiple Comparison Procedures (Holm-Sidak method)		t=2.963	0.01	0.004	Yes	EtOH vs Air
						All Pairwise Multiple Comparison Procedures (Holm-Sidak method)		t=1.915	0.013	0.057	No	Air+C6 vs EtOH+C6
						**All Pairwise Multiple Comparison Procedures (Holm-Sidak method)**		**t=1.495**	**0.017**	**0.137**	**No**	**EtOH+C6 vs Air**
						All Pairwise Multiple Comparison Procedures (Holm-Sidak method)		t=1.193	0.025	0.234	No	EtOH vs EtOH +C6
						All Pairwise Multiple Comparison Procedures (Holm-Sidak method)		t=1.035	0.05	0.302	No	Air + C6 vs EtOH
